# Supplementary material for: Health impacts and environmental footprints of diets that meet the Eatwell Guide recommendations: analyses of multiple UK studies
Source: BMJ Open. 2020 Aug 26;10(8):e037554. doi: 10.1136/bmjopen-2020-037554 (PMC7451532; doi:10.1136/bmjopen-2020-037554)
Supplement: Supplementary data [file bmjopen-2020-037554supp001.pdf]

Appendix

To: ***HEALTH IMPACTS AND ENVIRONMENTAL FOOTPRINTS OF DIETS THAT MEET THE EATWELL GUIDE RECOMMENDATIONS: ANALYSES OF MULTIPLE UK STUDIES***

Appendix 1 - Basic study descriptions for each cohort

Million Women Study

We used data from 464,078 participants of the Million Women Study. Participants were recruited from women (mean age 56 years) invited to the National Health service (NHS) breast cancer screening programme in England and Scotland between 1996 and 2001 [1]. Dietary intake was collected after an average of 3.3 years post recruitment using 130 semi-quantitative questions that were validated against a 7-day diet diary [2]. Nutrients were estimated by multiplying the frequency of consumption by portion size and nutrient composition of that item [3].

Total mortality was determined using death records obtained through linkage to centrally held NHS records. The Million Women Study protocol was approved by the Oxford and Anglia Multi-Centre Research Ethics committee. All participants provided written informed consent.

EPIC Oxford

We used data from 40,030 men and women (mean age 43 years) recruited throughout the UK (between 1993-2001) into the European Prospective Investigation into Cancer and Nutrition (EPIC)-Oxford cohort [4]. Dietary intake was collected using a validated 130-item semi-quantitative Food frequency questionnaire [5]. To estimate nutrient intakes, we multiplied frequencies of consumption by portion size and nutrient composition.

Total mortality was ascertained using death record linkage with the NHS Central register.

The EPIC-Oxford study protocol was approved by a Multi-Centre Research Ethics Committee (Scotland A Research Ethics Committee). All participants provided written informed consent.

UK Biobank

We used data from 53,614 middle-aged adults (mean age 56 years) participating in the UK Biobank who were recruited across the UK between 2006-2010 [6]. These were a subsample, that had completed a minimum of three 24-hour dietary recall questionnaires (the Oxford WebQ) [7]. Food and beverage frequency data, standard portion sizes and nutrient composition by item were multiplied to obtain nutrient intakes per day [8].

We linked participant data to the NHS Central register to obtain mortality information.

The UK Biobank was approved by the National Information Governance Board for Health and Social Care and the NHS North West Multi-Centre Research Ethics Committee. All participants provided written informed consent.

References

1. Green, J., et al., *Cohort profile: the million women study.* International journal of epidemiology, 2018. **48**(1): p. 28-29e.
2. Roddam, A.W., et al., *Reproducibility of a short semi-quantitative food group questionnaire and its performance in estimating nutrient intake compared with a 7-day diet diary in the Million Women Study.* Public Health Nutr, 2005. **8**(2): p. 201-13.
3. Key, T.J., et al., *Foods, macronutrients and breast cancer risk in postmenopausal women: a large UK cohort.* Int J Epidemiol, 2018: p. dyy238-dyy238.
4. Davey, G.K., et al., *EPIC–Oxford:lifestyle characteristics and nutrient intakes in a cohort of 33 883 meat-eaters and 31 546 non meat-eaters in the UK.* Public Health Nutrition, 2007. **6**(3): p. 259-268.
5. Bingham, S.A., et al., *Validation of dietary assessment methods in the UK arm of EPIC using weighed records, and 24-hour urinary nitrogen and potassium and serum vitamin C and carotenoids as biomarkers.* Int J Epidemiol, 1997. **26 Suppl 1**: p. S137-51.
6. Collins, R., *What makes UK Biobank special?* Lancet, 2012. **379**(9822): p. 1173-1174.
7. Greenwood, D.C., et al., *Validation of the Oxford WebQ online 24-hour dietary questionnaire using biomarkers.* Am J Epidemiol, 2019.
8. Bradbury, K.E., et al., *Dietary assessment in UK Biobank: an evaluation of the performance of the touchscreen dietary questionnaire.* J Nutr Sci, 2018. **7**: p. e6.

Appendix 2 – Eatwell Guide recommendations by age and sex

	5-6 years		7-10 years		11-14 years		15-18 years		19-64 years		65-74 years		75+ years	
Food group	Male	Female	Male	Female	Male	Female	Male	Female	Male	Female	Male	Female	Male	Female
Fruit & Vegetables (g/day)	400*	400g*	400g*	400g*	400g*	400g*	400g*	400g*	400g*	400g*	400g*	400g*	400g*	400g*
Oily Fish (g/day)	20g	20g	20g	20g	20g	20g	20g	20g	20g	20g	20g	20g	20g	20g
Other Fish (g/day)	20g	20g	20g	20g	20g	20g	20g	20g	20g	20g	20g	20g	20g	20g
Red and processed meat (g/day)	70	70	70	70	70	70	70	70	70	70	70	70	70	70
Fibre (g/day)	20**	20**	20**	20**	25**	25**	30**	30**	30**	30**	30**	30**	30**	30**
Salt (g/day)	3**	3**	5**	5**	6	6	6	6	6	6	6	6	6	6
Free Sugar (g/day)	19	19	24	24	30	30	30	30	30	30	30	30	30	30
Saturated fat (g/day)	18**	17 **	22**	20	30	20	30	20	30	20	30	20	30	20
Total fat (g/day)	58**	54**	71**	66**	97**	78**	97**	78**	97**	78**	91**	74**	89**	72**

**30g of dried fruit, max 150ml fruit juice or smoothie, and max 80g beans considered as one portion*

***Figures from PHE Government Dietary Recommendations document, derived from SACN. All other figures from the UK Eatwell Guide.*

Dietary recommendations of the Eatwell Guide

	Dietary recommendation and constraints
**Nutrients**	
Energy	2250 kcal (9414 MJ)*
Carbohydrates	≥50% of food energy
Free sugars	≤5% food energy
Fat	≤35% food energy
Saturated fat	≤11% food energy
Protein	≥14.5 & ≤15.5% of energy
Salt	≤ 6g/2363 mg/d sodium
Fibre (AOAC)	≥30g/d
**Foods**	

Fruits and vegetables†	≥5 portions a day
Fish	≥ 2 portions (2*140g) a week, one of which should be oily
Red and processed meat	≤70g/day

Table adapted from Public Health England (2016), Table 2.
**Energy intake recommendation assumes mixed population average.*
†Fruit and vegetable intake includes a maximum of: 1 portion of juice (from fruit / vegetable juice or that in a smoothie); 1 portion of beans; (portion sizes: 30g dried fruit; combined total of 150ml of fruit and / or vegetable juice and / or smoothie; 80g all other fruits & vegetables).

Appendix 3 – Cohort specific adjustments and cut-off values

Million Women Study

Associations were stratified by region and adjusted for smoking (never smoked, past smoker, current smoker <10 cigarettes smoked per day, current smoker 10-19 cigarettes smoked per day, current smoker ≥20 cigarettes smoked per day, current smoker and unknown number of cigarettes smoked per day), deprivation (tertiles), alcohol (0, 1-6, 7-14, ≥15 drinks per week), height cm (<155 ,155-164, ≥165), BMI kg/m^2 (<20, 20-24, 25-29, ≥30), strenuous exercise (<1 per week,≥1 per week), Hormone replacement therapy (HRT) use (never, past, current), educational attainment (none, technical/secondary/tertiary), self-reported hypertension (yes, no), and energy intake (quintiles).

EPIC Oxford

Associations were stratified by sex, region, and method of recruitment and adjusted for smoking (never smoked, past smoker, current smoker <10 cigarettes smoked per day, current smoker 10-19 cigarettes smoked per day, current smoker ≥20 cigarettes smoked per day, current smoker and unknown number of cigarettes smoked per day), deprivation (tertiles, unknown), alcohol intake (<1, 1-7 (ref.), 8-15, ≥16 grams per day, unknown), height cm (sex-specific tertiles, unknown), BMI kg/m^2(<20, 20-24, 25-29, ≥30), physical activity (inactive, low, moderate, high activity, unknown), HRT use ever (yes, no, unknown), educational attainment (national examination at age 16, national examination at ages 17-18, vocational qualification, , degree, unknown), self-reported high blood pressure (no, yes, unknown), and energy intake (sex-specific quintiles)

UK Biobank

Associations were stratified by sex and region and adjusted for smoking (never smoked, past smoker, current smoker <10 cigarettes smoked per day, current smoker 10-19 cigarettes smoked per day, current smoker ≥20 cigarettes smoked per day, current smoker and unknown number of cigarettes smoked per day), deprivation (tertiles, unknown), alcohol intake (none, <1, 1-<10, 10-<20, ≥20 grams per day, unknown), height cm (sex-specific tertiles, unknown), BMI kg/m^2 (<20, 20-24, 25-29, ≥30), physical activity (<10 excess METs, 10-<50 excess METs, ≥50 excess METs, unknown), qualification (national examination at age 16, national examination at ages 17-18, vocational qualification, college or university degree, other, unknown), HRT (never, past, current, unknown), self-reported high blood pressure (no, yes, unknown), and energy intake (sex-specific quintiles)

Appendix 4 – Results sensitivity analysis mortality RR

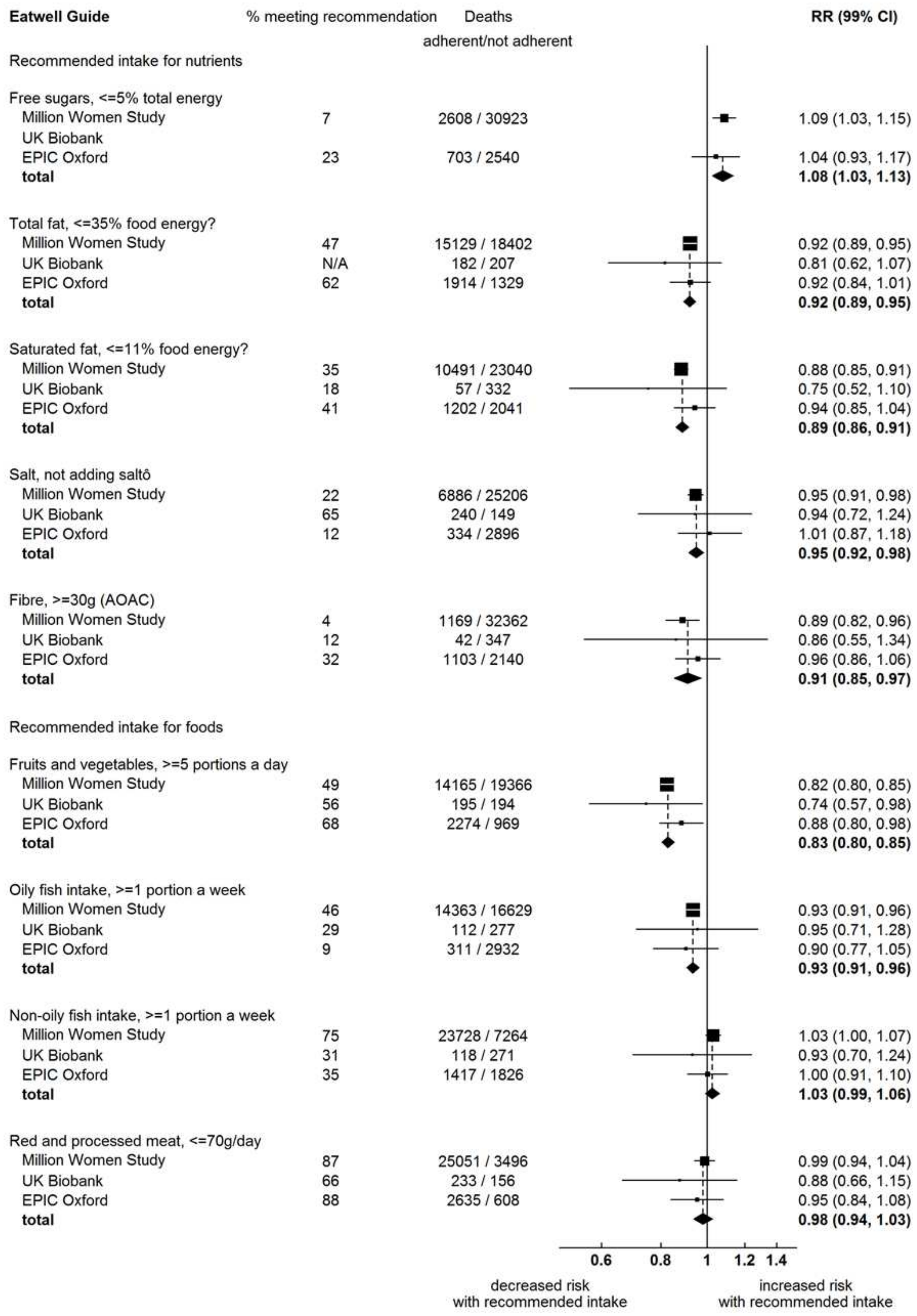

Figure S 1 Risk ratios for the association between some recommendations of the Eatwell Guide and total mortality without adjustment for smoking

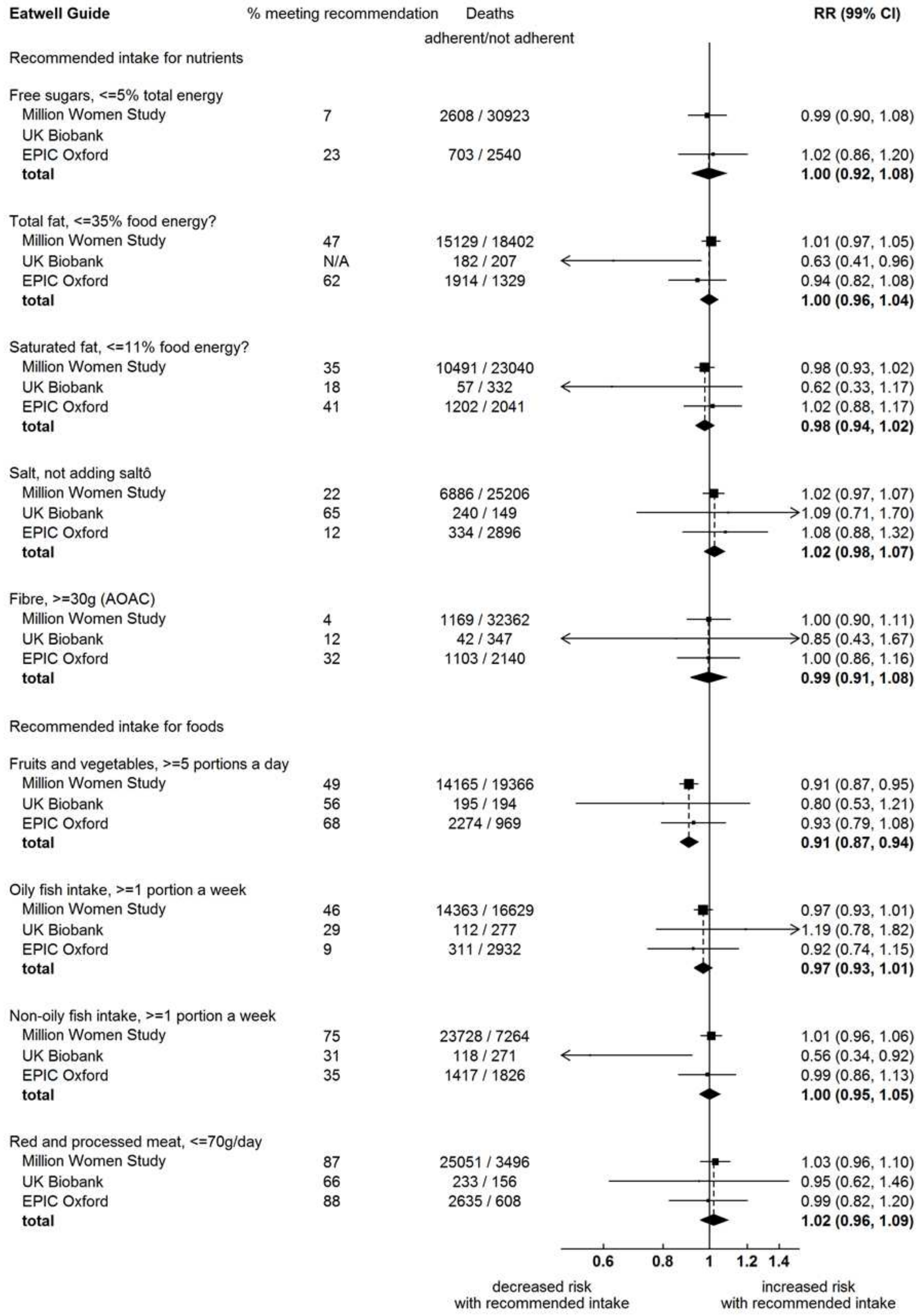

Figure S 2 Risk ratios for the association between some recommendations of the Eatwell Guide and total mortality <u>in never smokers</u>

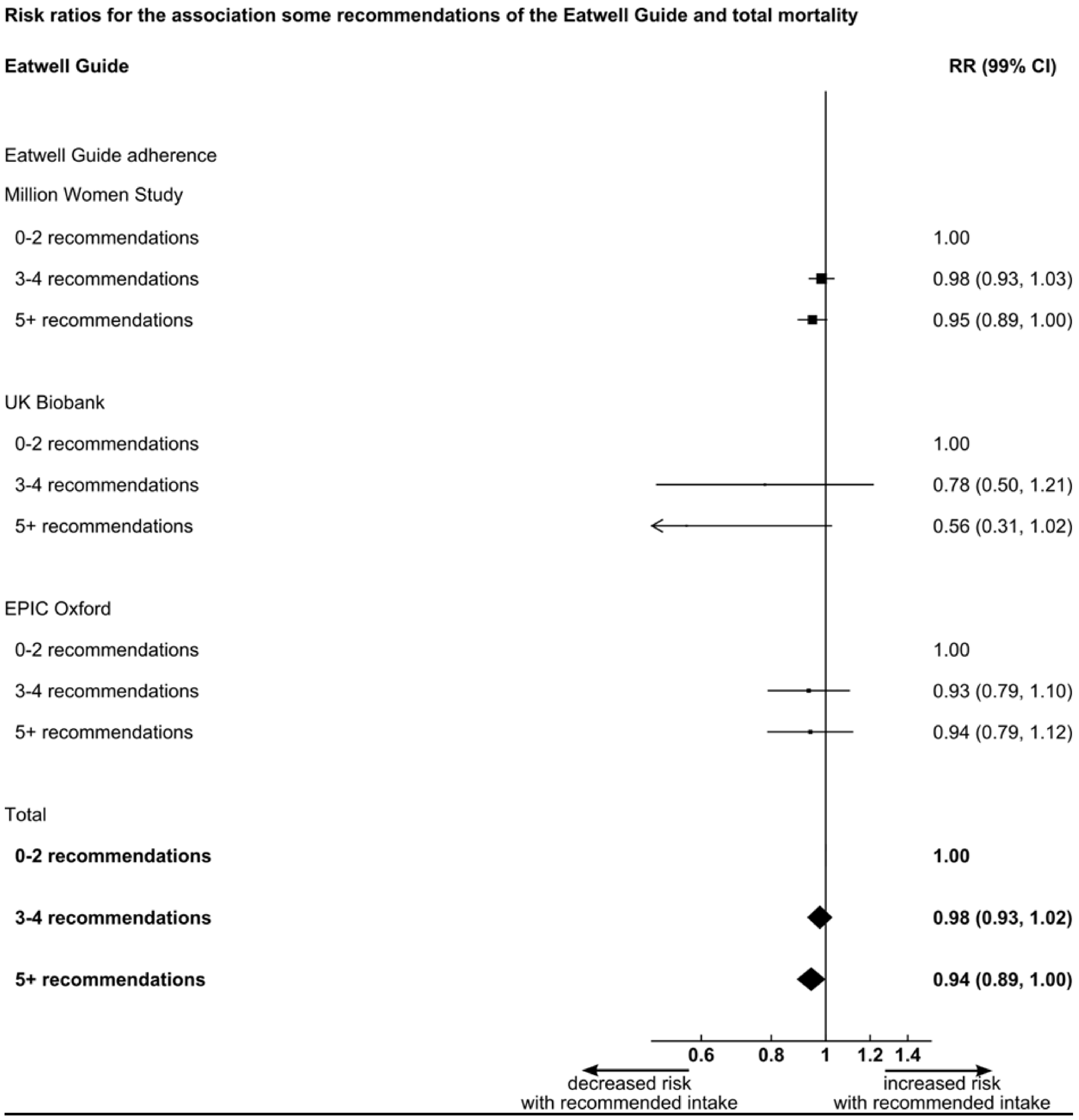

Figure S 3 Risk ratios for the association between the Eatwell Guide three level score and total mortality in never smokers

Appendix 5 – Detailed GHG emission and WFP calculations

Data from NDNS were matched to food-specific GHG emissions and blue water footprint estimates from previous studies using 173 SubFoodGroups available in the NDNS dataset. Where SubFoodGroups contained a number of foods with differing environmental footprints, weighted means according to consumption were used, and similarly where SubFoodGroups were composite foods including many ingredients (e.g. meat pies) recipe data were used to estimate the mean proportions of ingredients contained within the composite food. Recipe data were obtained from Sainsbury's (www.sainsbury.co.uk). Composite food and weighting data are available on request.

A number of additional SubFoodGroup categories were created in order to distinguish between categories where environmental footprints would be expected to differ greatly. These were:

- SubFoodGroup **6A** (wheat based breakfast cereals), **6B** (chocolate breakfast cereals), **6C** (oat based breakfast cereals), **6D** (maize based breakfast cereals), and **6E** (rice based breakfast cereals) were added in MainFoodGroup 6 (other breakfast cereals)
- SubFoodGroup **5A** (wheat based high fibre breakfast cereals), **5B** (chocolate high fibre breakfast cereals), **5C** (oat based high fibre breakfast cereals), and **5D** (rice based high fibre breakfast cereals) were added in MainFoodGroup 5 (high fibre breakfast cereals) and SubFoodGroup 5R was renamed (other high fibre breakfast cereals)
- SubFoodGroup **7C** (biscuits chocolate retail) was added in MainFoodGroup 7 (biscuits).
- SubFoodGroup **8F** (cakes chocolate) was added in MainFoodGroup 8 (buns cakes pastries and fruit pies)
- SubFoodGroups **13C** (non-dairy cream), **13D** (almond milk), **13E** (soya milk), **13F** (other non-dairy milk) and **13G** (chocolate milk) were added in MainFoodGroup 13 (other milk and cream) – Elmlea is not included in non dairy cream as it contains buttermilk
- SubFoodGroup **14C** (dairy free cheese) was added in MainFoodGroup 14 (cheese)
- SubFoodGroup **15E** (non dairy desserts) was added in MainFoodGroup 15 (yoghurt fromage frais and dairy desserts)
- SubFoodGroup **19B** (less than 1% dairy low fat spread) was added in MainFoodGroup 19 (low fat spread)
- SubFoodGroup **20A** (block margarine) was included in the dairy-free categories
- SubFoodGroup **20C** (other cooking fats and oils not PUFA) was renamed (other vegetable fats and oils) and a new SubFoodGroup **20B** (animal fats) was added in MainFoodGroup 20 (other margarine fats and oils)
- SubFoodGroup **21C** (less than 1% dairy reduced fat spread) was added in MainFoodGroup 21 (reduced fat spread)
- SubFoodGroup 37C (baked beans with sausages), moved to SubFoodGroup **25A** (manufactured pork products including ready meals) and categorised into MainFoodGroup 25 (pork products).
- SubFoodGroup **53A** (non dairy ice cream) was added in MainFoodGroup 53 (ice cream)

Codes 13A (infant formula), 54B (evening primrose oil and other plant oils), 54D (folic acid), 54E (iron only or with vitamin C), 54F (calcium only or with vitamin D), 54G (vitamins (two or

more including multivitamins), no minerals), 54H (minerals (two or more including multimins), no vitamins), 54I (vitamins and minerals (including multivits and minerals)), 54J (non-nutrient supplements including herbal), 54K (other nutrient supplements), 54L (vitamin C only), 54M (other single vitamins or minerals), 54M (cod liver oil and other fish oils), 54P (multivitamins and/or minerals with omega 3),

SubFood GroupCode	Food Group Name	Mean Greenhouse Gas emissions (kg) per kg food	Mean blue water footprint (litres) per kg food
1C	Pizza	3.51	369
1D	Pasta manufactured products and ready meals	1.00	0
1E	Other pasta including homemade dishes	1.00	0
1F	Rice manufactured products and ready meals	3.13	1071
1G	Other rice including homemade dishes	3.13	1071
1R	Other cereals	1.18	103
2R	White bread (not high fibre, not multiseed)	0.97	0
3R	Wholemeal bread	0.97	0
4R	Other bread	0.97	0
5A	Wheat based high fibre breakfast cereals	1.40	229
5B	Chocolate high fibre breakfast cereals	2.68	62
5C	Oat based high fibre breakfast cereals	1.41	109
5R	Other high fibre breakfast cereals	1.27	2
6A	Wheat based breakfast cereals	1.40	0
6B	Chocolate breakfast cereals	3.03	779
6C	Oat based breakfast cereals	1.41	109
6D	Maize based breakfast cereals	2.64	124
6E	Rice based breakfast cereals	2.85	1009
6R	Other breakfast cereals (not high fibre)	1.27	2
7A	Biscuits manufactured / retail	1.80	143
7B	Biscuits homemade	2.30	201
7C	Biscuits chocolate retail	8.14	135
8B	Fruit pies manufactured	0.95	86
8C	Fruit pies homemade	1.11	91
8D	Buns cakes and pastries manufactured	1.08	66
8E	Buns cakes and pastries homemade	3.31	34
8F	Cakes chocolate	3.11	126
9C	Cereal based milk puddings manufactured	2.00	170
9D	Cereal based milk puddings homemade	2.00	170

9E	Sponge puddings manufactured	1.79	17
9F	Sponge puddings homemade	1.79	17
9G	Other cereal based puddings manufactured	4.00	26
9H	Other cereal based puddings homemade	0.08	1
10R	Whole milk	1.53	28
11R	Semi skimmed milk	1.53	24
12R	Skimmed milk	1.53	24
13B	Cream (including imitation cream)	4.89	44
13C	Non dairy cream alternative	2.64	6
13D	Almond milk	0.99	73
13E	Soya milk	0.88	2
13F	Other non-dairy milk	2.65	57
13G	Chocolate milk	1.53	24
13R	Other milk	1.80	109
14A	Cottage cheese	15.00	132
14B	Cheddar cheese	8.87	132
14C	Dairy free cheese alternative	1.76	4
14R	Other cheese	8.87	59
15B	Yoghurt	2.00	31
15C	Fromage frais and dairy desserts	2.00	27
15D	Dairy desserts homemade	1.32	81
15E	Non dairy desserts	2.05	94
16C	Manufactured egg products including ready meals	3.51	4
16D	Other eggs and egg dishes including homemade	4.70	39
17R	Butter	9.00	194
18B	Polyunsaturated oils	3.59	235
19A	Polyunsaturated low fat spread	4.19	127
19B	Less than 1% dairy low fat spread	4.19	144
19R	Low fat spread not polyunsaturated	3.95	127
20A	Block margarine	4.19	144
20B	Animal fats	14.31	162
20C	Other vegetable fats and oils	4.65	853
21A	Reduced fat spread (polyunsaturated)	4.35	146
21B	Reduced fat spread (not polyunsaturated)	4.35	146
21C	Less than 1% dairy reduced fat spread	4.19	144

22A	Ready meals / meal centres based on bacon and ham	7.47	304
22B	Other bacon and ham including homemade dishes	10.70	321
23A	Manufactured beef products including ready meals	10.40	127
23B	Other beef and veal including homemade recipe dishes	16.50	205
24A	Manufactured lamb products including ready meals	30.48	275
24B	Other lamb including homemade recipe dishes	50.00	446
25A	Manufactured pork products including ready meals	8.85	321
25B	Other pork including homemade recipe dishes	10.00	293
26A	Manufactured coated chicken / turkey products	3.12	80
27A	Manufactured chicken products including ready meals	3.50	38
27B	Other chicken / turkey including homemade recipe dishes	3.50	38
28R	Liver and dishes	8.85	59
29R	Burgers and kebabs purchased	34.80	252
30A	Ready meals based on sausages	4.80	139
30B	Other sausages including homemade dishes	8.85	321
31A	Manufactured meat pies and pastries	8.63	209
31B	Homemade meat pies and pastries	13.96	251
32A	Other meat products manufactured including ready meals	11.50	594
32B	Other meat including homemade recipe dishes	2.84	37
33R	White fish coated or fried	3.36	0
34C	Manufactured white fish products including ready meals	4.55	0
34D	Other white fish including homemade dishes	4.55	0
34E	Manufactured shellfish products including ready meals	24.00	142
34F	Other shellfish including homemade dishes	24.00	142
34G	Manufactured canned tuna products including ready meals	4.55	0
34H	Other canned tuna including homemade dishes	4.55	0

35A	Manufactured oily fish products including ready meals	4.55	256
35B	Other oily fish including homemade dishes	4.55	256
36A	Carrots raw	1.28	0
36B	Salad and other raw vegetables	0.68	16
36C	Tomatoes raw	0.96	36
37A	Peas not raw	2.55	13
37B	Green beans not raw	0.50	40
37C	Baked beans	2.15	428
37D	Leafy green vegetables not raw	0.73	23
37E	Carrots not raw	1.28	0
37F	Tomatoes not raw	0.96	36
37I	Beans and pulses including ready meals and homemade dishes	1.51	21
37K	Meat alternatives including ready meals and homemade dishes	3.60	200
37L	Other manufactured vegetable products including ready meals	1.60	7
37M	Other vegetables including homemade dishes	0.58	39
38A	Chips purchased including take away	1.45	30
38C	Other manufactured potato products fried / baked	1.46	17
38D	Other fried / roast potatoes including homemade dishes	3.08	17
39A	Other potato products and dishes manufactured	1.20	46
39B	Other potatoes including homemade dishes	1.20	19
40A	Apples and pears not canned	0.70	52
40B	Citrus fruit not canned	0.40	93
40C	Bananas	0.90	49
40D	Canned fruit in juice	1.32	218
40E	Canned fruit in syrup	1.32	218
40R	Other fruit not canned	1.63	82
41A	Sugar	0.32	1
41B	Preserves	2.96	206
41R	Sweet spreads fillings and icing	7.14	269
42R	Crisps and savoury snacks	2.47	92
43R	Sugar confectionery	0.32	1
44R	Chocolate confectionery	1.07	78

45R	Fruit juice	1.01	157
47A	Liqueurs	1.00	1
47B	Spirits	1.00	1
48A	Wine	1.00	1
48B	Fortified wine	1.00	1
48C	Low alcohol and alcohol free wine	1.00	1
49A	Beers and lagers	3.80	14
49B	Low alcohol and alcohol free beer and lager	3.80	14
49C	Cider and perry	0.08	1
49D	Low alcohol and alcohol free cider and perry	3.80	1
49E	Alcoholic soft drinks	0.80	28
50A	Beverages dry weight	1.80	119
50C	Soup manufactured / retail	1.25	27
50D	Soup homemade	0.47	6
50E	Nutrition powders and drinks	0.00	0
50R	Savoury sauces pickles gravies and condiments	1.54	27
51A	Coffee (made up weight)	0.79	1955
51B	Tea (made up weight)	0.33	221
51C	Herbal tea (made up weight)	0.40	1
51D	Bottled water still or carbonated	0.40	1
51R	Tap water only	1.00	1
52A	Commercial toddlers drinks	0.00	0
52R	Commercial toddlers foods	0.00	0
53A	Non dairy ice cream	2.05	94
53R	Ice cream	3.82	44
55R	Artificial sweeteners	3.20	487
56R	Nuts and seeds	1.57	1415
57A	Soft drinks not low calorie concentrated	0.40	1
57B	Soft drinks not low calorie carbonated	0.40	1
57C	Soft drinks not low calorie rtd still	0.40	1
58A	Soft drinks low calorie concentrated	0.40	1
58B	Soft drinks low calorie carbonated	0.40	1
58C	Soft drinks low calorie rtd still	0.40	1
59R	Brown granary and wheatgerm bread	0.97	0
60R	1% fat milk	1.53	23
61R	Smoothies 100% fruit and / or juice	1.05	54

Appendix 6 - References for GHG and Blue WF figures

Amienyo D, Gujba H, Stichnothe H and Azapagic A (2013). Life cycle environmental impacts of carbonated soft drinks. *International Journal of Life Cycle Assessment* 18(1): 77-92.

Azapagic A, Bore J, Cheserek B, Kamunya S and Elbehri A (2016). The global warming potential of production and consumption of Kenyan tea. *Journal of Cleaner Production* 112(5): 4031-4040.

Bartzas G, Vamvuka D and Kommitsas K (2017). Comparative life cycle assessment of pistachio, almond and apple production. *Information Processing in Agriculture* 4(3): 188-198.

Bronmer E, Stratmann B and Quack D (2011). Environmental impacts of different methods of coffee preparation. *International Journal of Consumer Studies* 35(2): 212-220.

Buschspies B, Tolle SJ and Jungbluth N (2011). *Life Cycle Assessment of High-Sea Fish and Salmon Aquaculture.* ESU Services, Uster.

Carbon Trust (2017). *Report to Marlow Foods on Product Carbon Footprint Certification.*

Cichorowski G, Joa B, Hottenroth H and Schmidt M (2015). Scenario analysis of life cycle greenhouse gas emissions of Darjeeling tea. *International Journal of Life Cycle Assessment* 20(4): 426-439.

Clune S, Crossin E and Verghese K (2017). Systematic review of greenhouse gas emissions for different fresh food categories. *Journal of Cleaner Production* 140(2): 766-783.

Djekic I, Miocinovic J, Tomasevic I, Smigic N and Tomic N (2014). Environmental life-cycle assessment of various dairy products. *Journal of Cleaner Production* 68: 64-72.

Doublet G and Jungbluth N (2010). *Life Cycle Assessment of Drinking Darjeeling Tea.* ESU-Services, Uster.

Espinoza-Orias N, Stichnothe H and Azapagic A (2011). The carbon footprint of bread. *International Journal of Life Cycle Assessment* 16(4): 351-365.

Fantin V, Buttol P, Pergreffi R and Masoni P (2012). Life cycle assessment of Italian high quality milk production. A comparison with an EPD study. *Journal of Cleaner Production* 28: 150-159.

Finnegan W, Goggins J, Clifford E and Zhan X (2017). Global warming potential associated with dairy products in the Republic of Ireland. *Journal of Cleaner Production* 163: 262-273.

Frankowska A, Jeswani HK and Azapagic A (2019). Environmental impacts of vegetables consumption in the UK. *Science of the Total Environment* 682: 80-105.

Gonzalez-Garcia S, Gomez-Fernandez Z, Dias AC, Feijoo G, Moreira MT and Arroja L (2014). Life Cycle Assessment of broiler chicken production: a Portuguese case study. *Journal of Cleaner Production* 74: 125-134.

Huerta AR, Guereca LP and Lozano MSR (2016). Environmental impact of beef production in Mexico through life cycle assessment. *Resources, Conservation and Recycling* 109: 44-53.

Humbert S, Loerencik Y, Rossi V, Margni M and Jolliet O (2009). Life cycle assessment of spray dried soluble coffee and comparison with alternatives (drip filter and capsule espresso). *Journal of Cleaner Production* 17(15): 1351-1358.

Iriarte A, Almeida MG and Villalobos P (2014). Carbon footprint of premium quality export bananas: case study in Ecuador, the world's largest exporter. *Science of the Total Environment* 472: 1082-1088.

Jeswani HK, Burkinshaw R and Azapagic A (2015). Environmental sustainability issues in the food-energy-water nexus: breakfast cereals and snacks. *Sustainable Production and Consumption* 2: 17-28.

Jungbluth N (2006). *Comparison of the Environmental Impact of Tap Water vs. Bottled Mineral Water.* Swiss Gas and Water Association, Uster.

Kendall A, Marvinney E, Brodt S and Zhu W (2015). Life cycle-based assessment of energy use and greenhouse gas emissions in almond production, part I: analytical framework and baseline results. *Journal of Industrial Ecology* 19(6): 1008-1018.

Konstantas A, Stamford L and Azapagic A (2019). Economic sustainability of food supply chains: life cycle costs and value added in the confectionery and frozen desserts sectors. *Science of the Total Environment* 670: 902-914.

Konstantas A, Jeswani HK, Stamford L and Azapagic A (2018). Environmental impacts of chocolate production and consumption in the UK. *Food Research International* 106: 1012-1025.

Kristensen T, Soegaard K, Eriksen J and Mogensen L (2015). Carbon footprint of cheese produced on milk from Holstein and Jersey cows fed hay differing in herb content. *Journal of Cleaner Production* 101: 229-237.

Leinonen I, Williams AG, Wiseman J, Guy J and Kyriazakis I (2012). Predicting the environmental impacts of chicken systems in the United Kingdom through a life cycle assessment: Broiler production systems. *Poultry Science* 91(1): 8-25.

McCarty JA, Sandefur HN, Matlock M, Thoma G and Kim D (2014). Life cycle assessment of greenhouse gas emissions associated with production and consumption of peanut butter in the US. *Transactions of the ASABE* 57(6): 1741-1750.

Mekonnen MM and Hoekstra AY (2011). The green, blue and grey water footprint of crops and derived crop products. *Hydrology and Earth System Sciences* 15(5): 1577-1600.

Mekonnen MM and Hoekstra AY (2012). A global assessment of the water footprint of farm animal products. *Ecosystems* 15(3): 401-415.

Nguyen TLT, Hermansen JE and Mogensen E (2010). Environmental consequences of different beef production systems in the EU. *Journal of Cleaner Production* 18(8): 756-766.

Nilsson K, Flysjo A, Davis J, Sim S, Unger N and Bell S (2010). Comparative life cycle assessment of margarine and butter consumed in the UK, Germany and France. *International Journal of Life Cycle Assessment* 15(9): 916-926.

Nilsson K, Sund V and Floren B (2011). *The Environmental Impact of the Consumption of Sweets, Crisps and Soft Drinks.* Nordic Council of Ministers, Copenhagen.

Noya I, Aldea X, Gasol CM, Gonzalez-Garcia S, Amores MJ, Colon J, Ponsa S, Roman I, Rubio MA, Casas E, Moreira MT and Boschmonart-Rives J (2016). Carbon and water footprint of pork supply chain in Catalonia: From feed to final products. *Journal of Environmental Management* 171: 133-143.

Opio C, Gerber P, Mottet A, Falcucci A, Tempio G, MacLeod M, Vellinga T, Henderson B and Steinfeld H (2013). *Greenhouse gas emissions from ruminant supply chains – A global life cycle assessment*. Food and Agriculture Organization of the United Nations (FAO), Rome.

Pahlow M, van Oel PR, Mekonnen MM and Hoekstra AY (2015). Increasing pressure on freshwater resources due to terrestrial feed ingredients for aquaculture production. *Science of the Total Environment* 536: 847-857.

Poore J and Nemecek T (2018). Reducing food's environmental impacts through producers and consumers. *Science* 360: 987-992.

Prudencio da Silva V, van der Werf HMG, Soares SR and Corson MS (2014). Environmental impacts of French and Brazilian broiler chicken scenarios: An LCA approach. *Journal of Environmental Management* 133: 222-231.

Recanti F, Marveggio D and Dotelli G (2018). From beans to bar: a life cycle assessment towards sustainable chocolate supply chain. *Science of the Total Environment* 613-614: 1013-1023.

Recchia L, Cappelli A, Cini E, Pegna FG and Boncinelli P (2019). Environmental sustainability of pasta production chains: an integrated approach for comparing local and global chains. *Resources* 8(1): 56.

Scarborough P, Appleby PN, Mizdrak A, Briggs ADM, Travis RC, Bradbury KE and Key TJ (2014). Dietary greenhouse gas emissions of meat-eaters, fish-eaters, vegetarians and vegans in the UK. *Climatic Change* 125(2): 179-192.

Seabra JEA, Macedo IC, Chum HL, Faroni CE and Sarto CA (2011). Life cycle assessment of Brazilian sugarcane products: GHG emissions and energy use. *Biofuels, Bioproducts and Biorefining* 5(5): 519-532.

Sheane R, Lewis K, Hall P, Holmes-Ling P, Kerr A, Stewart K and Webb D (2011). *Identifying Opportunities to Reduce the Carbon Footprint Associated with the Scottish Dairy Chain – Main Report.* Edinburgh: Scottish Government.

Svanes E and Aronsson AKS (2013). Carbon footprint of a Cavendish banana supply chain. *International Journal of Life Cycle Assessment* 18(8): 1450-1464.

Wiedemann S, McGahan E, Murphy C, Yan M-J, Henry B, Thoma G and Ledgard S (2015). Environmental impacts and resource use of Australian beef and lamb exported to the USA determined using life cycle assessment. *Journal of Cleaner Production* 94: 67-75.

Ziegler F, Winther U, Skontorp Hognes E, Emmanuelsson A, Sund V, Ellingsen H (2012). The carbon footprint of Norwegian seafood products on the global seafood market. *Journal of Industrial Ecology* 17(1): 103-116

Appendix 7A – Calculation of weighted GHG and water footprint of the food group aggregate "fruit and vegetables" – based on proportional supply by crop and country of origin (FAOStat 2013 data)

Crops	% of total supply	GHG per kg	Blue WF per kg
Tomatoes	13.4	0.96	36
Bananas	7.8	1.07	49
Grapes**	6.3	1.34	298
Apples	6.1	0.75	77
Onions, dry	6.0	0.76	36
Carrots and turnips	5.8	1.28	0
Oranges	5.0	0.4	93
Cauliflowers and broccoli	2.5	1.03	53
Cabbages and other brassicas	2.5	0.67	7
Lettuce and chicory	2.3	1.59	41
Tangerines, mandarins, clementines, satsumas	2.0	0.4	93
Mushrooms and truffles	1.6	0.76	2
Chillies and peppers, green	1.6	0.76	2
Cucumbers and gherkins	1.5	0.2	12
Olives**	1.4	1.34	298
Pineapples**	1.4	1.34	298
Maize, green	1.3	0.76	2
Peas, green	1.3	2.55	23
Pears**	1.3	0.75	77
Melons, other (inc.cantaloupes)**	1.1	1.34	298
Strawberries	1.0	1.7	28
Lemons and limes	0.84	1.01	157
Plums and sloes	0.82	1.7	28
Pumpkins, squash and gourds	0.75	0.5	55
Peaches and nectarines**	0.64	1.34	298
Grapefruit (inc. pomelos)	0.60	0.4	93
Watermelons**	0.53	1.34	298
Apricots**	0.46	1.34	298
Sweet potatoes	0.46	0.5	55
Leeks, other alliaceous vegetables	0.42	0.71	15
Mangoes, mangosteens, guavas**	0.41	1.34	298
Beans, green	0.36	0.5	6
Avocados**	0.25	1.34	298
Onions, shallots, green*	0.24	0.76	36

* all other crops contribute less than 0.2% to total UK supply and were disregarded for calculations of weighted GHGe and WF of the fruit and vegetables aggregate.

** Classified as "other fruit" or "other vegetables" - without specific WFs

Appendix 7B - Proportions of main UK imported foods from different countries

For each food group, countries of origin were selected based on a database adapted from FAO Food Balance Sheet data (https://iopscience.iop.org/article/10.1088/1748-9326/9/3/034015/meta). All countries providing at least 10% of the total availability for that food group were included in environmental footprinting, and for the remainder of supply global average figures were applied. Where country-specific footprint data were not available, footprints from the most similar country or a global average were applied.

Food	Country	Proportion of UK consumption
ALMONDS	USA	0.72
	Australia	0.1
	Spain	0.14
	Global	0.04
APPLE JUICE	France	0.14
	South Africa	0.1
	UK	0.36
	Global	0.4
APPLES	France	0.14
	South Africa	0.1
	UK	0.36
	Global	0.4
ASPARAGUS	Mexico	0.11
	Peru	0.46
	Spain	0.12
	UK	0.26
	Global	0.05
AVOCADO	Chile	0.22
	Israel	0.16
	Peru	0.21
	South Africa	0.27
	Global	0.14
BANANAS	Colombia	0.23

	Costa Rica	0.18
	Dominican Republic	0.18
	Ecuador	0.14
	Global	0.27
BARLEY	UK	0.81
	Global	0.19
BEEF	UK	0.76
	Ireland	0.17
	Global	0.07
BLUEBERRIES	Netherlands	0.34
	Poland	0.3
	Global	0.45
CAULIFLOWER/BROCCOLI	UK	0.47
	Spain	0.43
	Global	0.1
CHICKPEAS	Argentina	0.11
	Australia	0.21
	Canada	0.14
	Mexico	0.12
	Global	0.42
COFFEE	Brazil	0.26
	Colombia	0.1
	Indonesia	0.13
	Vietnam	0.25
	Global	0.26
COCOA BUTTER	Ghana	0.27
	Cote d'Ivoire	0.44
	Nigeria	0.1
	Global	0.19

COCOA PASTE	Ghana	0.27
	Cote d'Ivoire	0.44
	Nigeria	0.1
	Global	0.19
COCONUTS	Indonesia	0.24
	Malaysia	0.11
	Philippines	0.48
	Global	0.17
CUCUMBERS	Netherlands	0.34
	Spain	0.33
	UK	0.3
	Global	0.03
GARLIC	China	0.41
	Spain	0.52
	Global	0.07
GRAPES	Chile	0.1
	France	0.1
	Italy	0.15
	Spain	0.1
	Turkey	0.18
	Global	0.37
GREEN BEANS	Egypt	0.15
	Kenya	0.32
	UK	0.32
	Global	0.21
GROUNDNUTS	USA	0.26
	Nicaragua	0.13
	China	0.14
	Argentina	0.33
	Global	0.14

HAZELNUTS	Georgia	0.18
	Italy	0.1
	Turkey	0.69
	Global	0.03
LEMON JUICE	Argentina	0.16
	Brazil	0.13
	Spain	0.47
	Global	0.24
LEMONS	Argentina	0.16
	Brazil	0.13
	Spain	0.47
	Global	0.24
LENTILS	Canada	0.63
	Turkey	0.26
	Global	0.11
LETTUCE	Spain	0.49
	UK	0.4
	Global	0.11
MAIZE	France	0.27
	Ukraine	0.18
	Global	0.55
MILLET	Russia	0.32
	France	0.24
	Ukraine	0.13
	India	0.1
	Global	0.21
NUTS, OTHER	China	0.13
	Turkey	0.3
	USA	0.12
	Global	0.45

OLIVE OIL	Italy	0.18
	Spain	0.59
	Global	0.23
ONIONS	Netherlands	0.23
	Spain	0.16
	UK	0.44
	Global	0.17
ORANGE JUICE	Brazil	0.3
	South Africa	0.13
	Spain	0.28
	USA	0.1
	Global	0.19
ORANGES	Brazil	0.3
	South Africa	0.13
	Spain	0.28
	USA	0.1
	Global	0.19
PALM OIL	Indonesia	0.43
	Malaysia	0.28
	Papua New Guinea	0.2
	Global	0.09
PEACHES	Italy	0.14
	Spain	0.73
	Global	0.13
PEARS	Netherlands	0.32
	South Africa	0.17
	UK	0.13
	Global	0.38
PEPPERS AND CHILLIES	Netherlands	0.38
	Spain	0.34

	UK	0.11
	Global	0.17
PINEAPPLES	Costa Rica	0.7
	Thailand	0.14
	Global	0.16
POTATOES	UK	0.72
	Netherlands	0.11
	Global	0.17
PRUNES (PLUMS)	Chile	0.24
	Spain	0.16
	UK	0.11
	USA	0.14
	Global	0.35
RAISINS	Turkey	0.3
	USA	0.3
	South Africa	0.1
	Chile	0.1
	Global	0.2
RICE (WHITE)	India	0.26
	Spain	0.18
	Italy	0.13
	Pakistan	0.11
	Global	0.32
RICE (BROWN)	India	0.26
	Spain	0.18
	Italy	0.13
	Pakistan	0.11
	Global	0.32
SOYA PROTEIN	Brazil	0.44
	Argentina	0.31

	USA	0.16
	Global	0.09
SOYBEANS	Argentina	0.31
	Brazil	0.44
	USA	0.16
	Global	0.09
SOYBEANS FOR MILK	Brazil	0.44
	Argentina	0.31
	USA	0.16
	Global	0.09
SPINACH	Italy	0.11
	Spain	0.7
	Global	0.19
SUGAR	UK	0.38
	Global	0.62
SUNFLOWER OIL	Argentina	0.12
	France	0.14
	Ukraine	0.31
	Global	0.43
SUNFLOWER SEEDS	Argentina	0.12
	France	0.14
	Ukraine	0.31
	Global	0.43
TEA	India	0.16
	Indonesia	0.15
	Kenya	0.39
	Global	0.3
TOMATO PASTE	Italy	0.39
	Spain	0.2
	Portugal	0.13

	Global	0.28
TOMATOES	Italy	0.39
	Spain	0.2
	Portugal	0.13
	Global	0.28
WHEAT FLOUR	UK	0.67
	Global	0.33

Appendix 8 – Differences in EWG dietary guidelines adherence

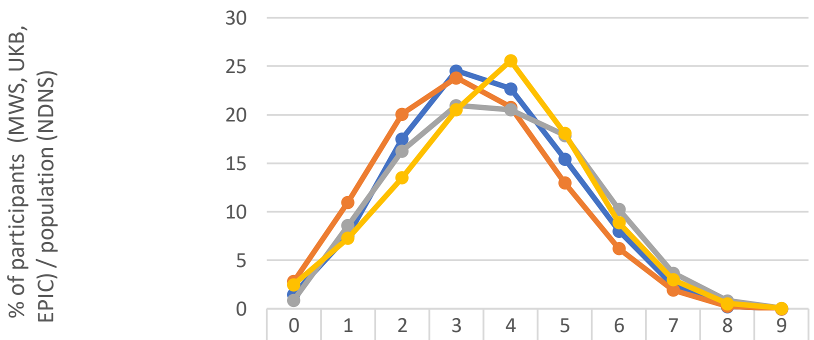

	0	1	2	3	4	5	6	7	8	9
Million Women Study	1.48	7.56	17.54	24.55	22.71	15.44	8.04	2.40	0.26	0.01
UK Biobank	2.84	11.00	20.09	23.83	20.80	13.00	6.23	1.93	0.27	NA
EPIC Oxford	0.89	8.58	16.28	20.97	20.54	17.88	10.25	3.69	0.85	0.08
NDNS	2.50	7.28	13.52	20.52	25.58	18.08	8.90	3.01	0.54	0.08
