# Supplementary material for: Health impacts and environmental footprints of diets that meet the Eatwell Guide recommendations: analyses of multiple UK studies
Source: BMJ Open. 2020 Aug 26;10(8):e037554. doi: 10.1136/bmjopen-2020-037554 (PMC7451532; doi:10.1136/bmjopen-2020-037554)
Supplement: Author's manuscript [file bmjopen-2020-037554.draft_revisions.pdf]

BMJ Open

BMJ Open is committed to open peer review. As part of this commitment we make the peer review history of every article we publish publicly available.

When an article is published we post the peer reviewers' comments and the authors' responses online. We also post the versions of the paper that were used during peer review. These are the versions that the peer review comments apply to.

The versions of the paper that follow are the versions that were submitted during the peer review process. They are not the versions of record or the final published versions. They should not be cited or distributed as the published version of this manuscript.

BMJ Open is an open access journal and the full, final, typeset and author-corrected version of record of the manuscript is available on our site with no access controls, subscription charges or pay-per-view fees (http://bmjopen.bmj.com).

If you have any questions on BMJ Open's open peer review process please email info.bmjopen@bmj.com

BMJ Open

SUSTAINABLE FOOD SYSTEMS & DIETS IN THE UK - The Eatwell Guide as a blueprint for healthy and sustainable diets in the UK

Journal:	*BMJ Open*
Manuscript ID	bmjopen-2020-037554
Article Type:	Original research
Date Submitted by the Author:	12-Feb-2020

Complete List of Authors:	Scheelbeek, Pauline; London School of Hygiene and Tropical Medicine Faculty of Epidemiology and Population Health, Population Health<br>Green, Rosemary; London School of Hygiene and Tropical Medicine, Population health<br>Papier, Keren; University of Oxford Nuffield Department of Population Health, Cancer Epidemiology Unit<br>Knuppel, Anika ; University of Oxford Nuffield Department of Population Health, Cancer Epidemiology Unit<br>Alae-Carew, Carmelia; London School of Hygiene and Tropical Medicine, Epidemiology & Biostatistics<br>Balkwill, Angela; Oxford University<br>Key, Timothy; University of Oxford, Cancer Epidemiology Unit, Nuffield Department of Population Health<br>Beral, Valerie; Oxford University, Nuffield Department of Population Health<br>Dangour, Alan; London School of Hygiene & Tropical Medicine, Nutrition and Public Health Intervention Research

Keywords:	EPIDEMIOLOGY, NUTRITION & DIETETICS, PREVENTIVE MEDICINE, PUBLIC HEALTH

SCHOLARONE™
Manuscripts

I, the Submitting Author has the right to grant and does grant on behalf of all authors of the Work (as defined in the below author licence), an exclusive licence and/or a non-exclusive licence for contributions from authors who are: i) UK Crown employees; ii) where BMJ has agreed a CC-BY licence shall apply, and/or iii) in accordance with the terms applicable for US Federal Government officers or employees acting as part of their official duties; on a worldwide, perpetual, irrevocable, royalty-free basis to BMJ Publishing Group Ltd ("BMJ") its licensees and where the relevant Journal is co-owned by BMJ to the co-owners of the Journal, to publish the Work in this journal and any other BMJ products and to exploit all rights, as set out in our licence.

The Submitting Author accepts and understands that any supply made under these terms is made by BMJ to the Submitting Author unless you are acting as an employee on behalf of your employer or a postgraduate student of an affiliated institution which is paying any applicable article publishing charge ("APC") for Open Access articles. Where the Submitting Author wishes to make the Work available on an Open Access basis (and intends to pay the relevant APC), the terms of reuse of such Open Access shall be governed by a Creative Commons licence – details of these licences and which Creative Commons licence will apply to this Work are set out in our licence referred to above.

Other than as permitted in any relevant BMJ Author's Self Archiving Policies, I confirm this Work has not been accepted for publication elsewhere, is not being considered for publication elsewhere and does not duplicate material already published. I confirm all authors consent to publication of this Work and authorise the granting of this licence.

For peer review only

SUSTAINABLE FOOD SYSTEMS & DIETS IN THE UK

The Eatwell Guide as a blueprint for healthy and sustainable diets in the UK

**Pauline F.D. Scheelbeek – Centre on Climate Change and Planetary Health, London School of Hygiene & Tropical Medicine, London UK*
Rosemary Green – Centre on Climate Change and Planetary Health, London School of Hygiene & Tropical Medicine, London UK
Keren Papier –Nuffield Department of Population Health, University of Oxford, Oxford, UK
Anika Knuppel – Nuffield Department of Population Health, University of Oxford, Oxford, UK
Carmelia Alae-Carew – Department of Population Health, London School of Hygiene & Tropical Medicine, London UK
Angela Balkwill - Nuffield Department of Population Health, University of Oxford, Oxford, UK
Tim Key - Nuffield Department of Population Health, University of Oxford, Oxford, UK
Valerie Beral - Nuffield Department of Population Health, University of Oxford, Oxford, UK
Alan D. Dangour – Centre on Climate Change and Planetary Health, London School of Hygiene & Tropical Medicine, London UK

**Corresponding author – Keppel Street, London WC1E 7HD, United Kingdom - pauline.scheelbeek@lshtm.ac.uk - +44.207.612.7914*

***Keywords:** sustainable food systems; Eatwell Guide; nutritional health; dietary footprint; environmental footprint*

Word count (excluding title page, references, figures): 2294

Abstract

Background – Dietary guidelines recognise the role of diets for supporting population health and increasingly include considerations for environmental sustainability. In the UK, the "Eatwell Guide" (EWG) recommendations provide guidance on how to achieve diets with a balance of healthy and sustainable food. Our study explores the health impacts and environmental consequences of adherence to EWG recommendations in the UK.

Methods – We analysed data from EPIC-Oxford, UK Biobank, the Million Women Study and the nationally-representative National Diet and Nutrition Survey (NDNS). Dietary intakes were dichotomized to reflect individual adherence to nine EWG recommendations and participants were assigned to "very low" (0-2 recommendations), "low" (3-4 recommendations), or "intermediate to high" (5-9 recommendations) EWG-adherence groups. We used multivariable-adjusted Cox proportional hazards regression models to assess associations between different levels of EWG adherence and risk of total mortality. Environmental footprints of individual food items were estimated based on previously published data for greenhouse gas emissions and blue water footprints, and matched to individual diets in the NDNS database using proportional weighting according to the major countries of origin of UK-consumed foods. Average environmental footprints according to EWG adherence categories were calculated.

Results – Less than 0.1% of the NDNS sample adhere to nine EWG recommendations and 30.6% adhere to at least five recommendations. Compared to "very low" adherence to EWG recommendations, "intermediate to high adherence" was associated with 7% reduced risk of mortality and -1.6kg CO_2eq/day (or 30%) lower dietary greenhouse gas emissions. Dietary water footprints were similar across EWG adherence groups.

For peer review only - http://bmjopen.bmj.com/site/about/guidelines.xhtml

Discussion – The health and environmental benefits of greater adherence to EWG recommendations support increased government efforts to encourage improved diets in the UK that are essential for the health of people and the planet in the Anthropocene epoch.

Strengths and limitations of this study

- This is the first study (in a UK context) using empirical data to study health impacts and environmental consequences of sustainable diets. Besides greenhouse gas emissions, water footprints of sustainable diets were assessed.
- Data quality is high: the study uses the largest and best health datasets available, addressing an important question that is highly relevant for UK policy.
- The study methods and results could be used in contexts outside the UK: the provided methods can be replicated in other settings and the EatWell Guide dietary recommendations share many features of healthy lower environmental impact diets. Therefore our findings have wider applicability.
- Despite several sensitivity analyses, there might be residual confounding (i.e. unmeasured differences between people who eat different diets) distorting our findings.
- Although the evidence base and quality of methods and metrics for environmental footprints are rapidly improving, uncertainty about the exact measurements of water footprints and greenhouse gas emissions of food items and diets in general remain.

Background

Diets are likely to play a crucial role in the Anthropocene in supporting population health and safeguarding environmental sustainability of future generations. Current diets are associated with a high burden of disease: globally ~1.9 billion adults are overweight or obese, 462 million are underweight [1], and over 30% of the world's population suffers from deficiencies of essential nutrients [2]. The food system that produces these diets is also responsible for 21-37% of global greenhouse emissions [3] and agriculture alone accounts for ~70% of fresh water withdrawal [4]. There is an urgent need for significant transformations of the food system to produce diets that address both health and environmental concerns, and evidence on the recommended composition of these diets is expanding rapidly [5]. While the specific composition of such diets has been shown to vary considerably culturally and regionally compared to existing consumption patterns, these diets typically have substantially greater fruit, vegetable and legume content as well as moderate content of animal sourced foods (e.g.[5-7]).

Governments are increasingly including both health and environmental considerations in their recommended dietary guidelines. In the UK, Public Health England produced the "Eatwell Guide" (EWG) as a "policy tool to define government recommendations on eating healthily and achieving a balanced diet" [8]. From a health perspective, the EWG promotes, for example, fruit, vegetable and fibre consumption, whilst recommending a limited consumption of sugar and processed meats [9]:Adhering to these individual guidelines has been associated with several health benefits including improved cardiovascular health [10] and reduced cancer risk[11, 12]. From an environmental perspective the EWG mentions the importance of a "balance of healthier and more sustainable food" , while providing information about protein alternatives, such as beans, peas and lentils that typically have a lower environmental footprint than animal source food protein sources[12-15]. Compared to current diets, the EWG recommendations are designed to have on average lower environmental footprints (GHG emissions, water and land use requirements) [16], but no analysis has specifically addressed the environmental consequences of shifts from current to EWG-adherent diets.

In this report we use high-quality data from three major UK cohort studies to assess the health impacts associated with adherence to EWG dietary guidelines in the UK and using nationally-

For peer review only

representative dietary intake data we estimate the environmental footprints of UK diets with varying degrees of adherence to EGW recommendations.

Methods

Datasets

We used four high-quality data sources in this paper (detailed description of each database provided in Appendix 1). The databases from EPIC-Oxford (EPIC-Ox) [17], UK Biobank (UKB) [18] and the Million Women Study (MWS) [19] contain comprehensive health information, linked death registration data, as well as dietary intake data. These three datasets were used to estimate the effects on health of adherence to EWG recommendations. Details on the specific datasets have been published elsewhere, however in short: Participants of the ongoing MWS were recruited from women invited for breast cancer screening in England and Scotland between 1996 and 2001. Dietary intake was collected using semi-quantitative questions and total mortality was determined using death records. We used data from 464,078 participants of the MWS database. In the ongoing EPIC-Ox study – that involves both male and female participants - dietary intake was collected using a food-frequency questionnaire, whilst total mortality was ascertained using death record linkage. We used data from 40,030 men and women of the EPIC-Ox database. For the UKB study, middle-aged adults were recruited between 2006-2010. A sub-sample completed a minimum of three 24-hour dietary recall questionnaires. Participant data can be linked to the NHS Central register to obtain mortality information. We used data from 53,614 participants of the UKB study. Finally, the National Diet and Nutrition Survey (NDNS) [20] contains nationally representative detailed dietary intake data that were used to analyse the diet-related environmental footprint of NDNS participants with different levels of EWG adherence. We excluded children <5 years of age from the NDNS data, as the EWG recommendations are not applicable to this age group.

Eatwell Guide dietary recommendations

Dietary intakes reported in each of the four databases were compared to recommended intakes by the EWG and dichotomized (yes/no) to reflect individual adherence to EWG recommendations (recommendations by age and sex provided in Appendix 2). Nine food and nutrient groups with recommended levels of consumption specified in the EWG were considered: fruit and vegetables; oily fish; other fish; red and processed meat; total fibre; total salt; free sugars; saturated fatty acids; and total fat. Two further EWG recommendations on protein and carbohydrates were excluded as significant heterogeneity across foodstuffs limits conversion from % of food energy intake to grams per day (see also [21]). Participants were grouped into three categories of adherence based on the number of dietary recommendations met (total = 9): very low adherence [score 0-2]; low adherence [score 3-4]; and intermediate to high adherence [score 5-9].

Health impacts

We used multivariable-adjusted Cox proportional hazards regression models to assess associations between adherence to the EWG dietary guidelines and risk of total mortality, ascertained through death registries using participant data of EPIC-Oxford (EPIC-Ox), the Million Women Study (MWS), and a subset of UK Biobank with detailed dietary data (UKB).

For peer review only - http://bmjopen.bmj.com/site/about/guidelines.xhtml

The mean follow-up time was 21.0 years in EPIC Ox, 10.5 years in MWS and 3.9 years in UKB.

Participants in each database were excluded from the analysis sample if: (1) they had prevalent and/or unknown status of malignant cancer, diabetes or cardiovascular disease (data based on self-report and health record data) or rated their overall health as either poor or fair at recruitment; (2) they had energy intakes outside the ranges 2,093-14,654 kJ for women and 3,349-16,747 kJ for men, and did not report: a change in diet because of illness (MWS), not eating or drinking normally because of illness or fasting (UKB), because of stomach problems, bowel problems or diabetes (EPIC-Ox) and in UKB had completed a minimum of three WebQ questionnaires; (3) they were lost to follow-up during the first 5 years of follow-up (MWS and EPIC-Ox only), and; (4) their smoking status was unknown.

Associations were stratified by sex, region and method of recruitment (in addition to the general recruitment strategy, specific underrepresented groups were targeted for recruitment by leaflets – which could have introduced selection bias), where appropriate. All analyses were adjusted for smoking, deprivation, alcohol consumption, height, body mass index (BMI), exercise levels, hormone replacement therapy use, education, high blood pressure or hypertension and energy intake (Appendix 3 for details). We performed a set of seven sensitivity analyses, comparing the above model with a) an unadjusted model, models without adjustment for b) energy, c) height, d) BMI or e) smoking, f) a model mutually adjusting for all other eight food groups, and g) a model excluding smokers (Appendix 4).

Environmental footprints

We used data from NDNS waves 5-9 (2012-2017) to map the environmental footprints of diets in the UK. The database comprises detailed dietary data for 5,747 individuals aged five years and over, grouped into 158 distinct food group aggregates. Data collection methods are described in detail elsewhere [22]. We used the Food and Agriculture Organization (FAO) bilateral trade database [23] to estimate the mean proportion of each food group imported from outside the UK. The trade database includes bilateral data on exports and imports of all food and agricultural products reported by all the countries in the world

Greenhouse Gas Emissions - Emissions of GHGs across the life cycle (kg CO2e/kg food) for the 158 distinct food group aggregates were derived from published data (Appendix 5&6). A weighted average of GHG emissions was calculated based on consumption of individual foods within each food group and proportion of supply from different countries. For foods entirely or more than 90% produced in the UK, UK-specific data were used. A weighted average for GHG emissions was applied for imported foods based on the proportion of total supply from various countries (Appendix 7).

Water footprints (WFs) - The blue (ground and surface water) WF (L/g food) of crop and livestock products were derived from published data for 1995-2005 from the Water Footprint Network (WFN, [24]) (Appendix 5&6). For foods entirely or more than 90% produced in the UK, UK-specific WFN values were used. Imported food groups were assigned weights proportional to percentage of overall supply of each major exporting country to the UK, multiplied by WFN estimates for that particular country and food group (Appendix 7).

The estimated GHG emissions and WFs associated with each food group were used to quantify total environmental footprints associated with the daily diet of each participant in the NDNS database. We compared GHG emissions and WFs of diets of those adhering and those

For peer review only

not adhering to each EWG dietary guidelines, and estimated the mean change in environmental footprint that would occur if individuals shifted from low to intermediate/high adherence to the EWG guidelines.

Results

EWG-adherence

Less than 0.1% of the NDNS sample (0.078%) adhered to all nine EWG recommendations (Figure 1A), with the largest proportion of the population (44%) adhering to 3-4 guidelines. The most commonly unmet recommendations included those on consumption of dietary fibre and oily fish (7.2% and 16.8% adherence respectively), while more than 50% of the population met total and saturated fat, salt and red and processed meat recommendations (Figure 1B). Adherence to the EWG recommendations in EPIC-Ox, MWS and UKB showed a similar pattern to that in the NDNS data set (Appendix 8).

Figure 1: Adherence to the Eatwell Guide recommendations by the UK population - based on data from wave 5-9 of the National Dietary and Nutrition Survey (NDNS): A) total number of recommendations met by % of UK population; B) adherence to specific recommendations

Health effects of adherence to EWG recommendations

Compared with those who had a very low adherence to the EWG, individuals with intermediate to high adherence had a 7% [95% CI: 3 to 10%] reduced risk of total mortality (Figure 2). Sensitivity analysis identified smoking as an important confounder, but no other significant and consistent deviations in mortality outcomes compared to the main model.

Figure 2: Forest plot showing the study specific (MWS, UKB & EPIC-Ox) and pooled mortality risk ratios comparing very poor adherence to EWG rocmmendations (score 0-2) with poor adherence (score 3-4) and intermediate to high adherence (score 5-9).

Adherence to the recommendation on fruit and vegetable consumption was independently associated with the largest reduction in total mortality risk: a reduction of 10% [RR: 0.90; 95% CI: 0.88 – 0.93] (Figure 3; attenuated to 9% in models adjusting for all other EWG recommendations see Appendix 4). Meeting the recommendations on saturated fat and oily fish consumption showed smaller health benefits with 5% and 3% reductions in mortality respectively (both attenuated to 3% in models adjusting for all other EWG recommendations see [Appendix 4]). There was no consistent evidence of an effect on mortality risk associated with adherence to other EWG recommendations (Figure 3 & Appendix 4).

*Figure 3: Mortality risk ratios for the association between adhering to specific EWG recommendations and total mortality. * Recommendation was based on food energy and was therefore adapted to ≥47% of total energy [25]. *Adapted to*

For peer review only

<=33% of total energy [25]. †Adapted to <=10% of total energy [25]. ‡Information on salt intake was ascertained from the variable 'Never adding salt to food at the table or cooking' in the MWS and in the EPIC-Oxf study; and from the variable 'Not reporting having added salt to food (excluding during cooking)' in any of the WEBQs included in the UKB. §Fibre intake in the study was

		Dietary Recommendation							
		Fruit & vegetables		Oily fish		Non-oily fish		Red & processed meat	
Metric	Unit	*Meeting recommendation*	*not meeting recommendation*	*Meeting recommendation*	*not meeting recommendation*	*Meeting recommendation*	*not meeting recommendation*	*Meeting recommendation*	*not meeting recommendation*
**Weighted average consumption**	***g/day (SE)***	561 (6.47)	218 (2.00)	40.3 (1.23)	1.14 (0.08)	39.7 (0.85)	3.61 (0.13)	31.8 (0.50)	113 (1.30)

determined using the Englyst method and the recommendation was therefore adapted to >=22.6g/d of Englyst fibre.

Environmental footprints of diets

Individuals with intermediate to high adherence to EWG recommendations showed a reduction in average dietary GHG footprints - compared to those with low and very low EWG-adherence - of 12% and 30% respectively: an average of 3.8kg CO_2eq/day (95% CI: 3.7 to 3.9kg CO_2eq /day) , (4.3kg CO_2eq/day [95% CI: 4.1 to 4.4kg CO_2eq /day] and 5.4 kg CO_2eq/day [95% CI: 5.2 to 5.6kg CO_2eq /day] for intermediate to high (score 5-9), low (score 3-4) and very low (score 0-2) EWG adherence respectively) Dietary blue WFs were similar across adherence groups (Figure 4): 637kg CO2eq /day [95% CI 590 to 683], 590kg CO2eq /day [95% CI 558 to 622] and 612kg CO2eq /day [95% CI 571 to 654] respectively for very low, low and intermediate to high adherence to the EWG recommendations. GHG emissions and WFs changed marginally when adjusting for dietary energy intake (Appendix 9).

Figure 4: Average daily greenhouse gas emissions in kg CO_2eq and average daily dietary water footprints comparing diets with very low (score 0-2), low (score 3-4) and intermediate to high adherence (score 5-9) to the Eatwell Guide dietary guidelines.

Mean difference in consumption (in g per day) of foods between EWP adherent and non-adherent individuals was large (Table 1). Associated differences in dietary GHG emissions were small for fruit and vegetables, oily fish and non-oily fish consumption, and adherence to the recommendation on red and processed meat was associate with lower GHG emissions (-1.48kg CO2eq/day; 95% CI -1.79 to -1.18) (Table 1). Differences in blue water footprints were small for oily fish and non-oily fish consumption, and adherence to the fruit and vegetable recommendation was associated with a larger blue water footprint (+28.5 litres per person per day; 95% CI: 17.4 to 39.8) while adherence to the red and processed meat recommendation was associated with a lower blue water footprint (-22.5 litres per person per day; 95% CI: -22.7 to -22.3).

For peer review only

**Difference in average consumption**	**g/day**	**+343**	**+39.2**	**+36.1**	**-81.2**
**Mean difference in GHGe achieved by switching to meeting guideline**	**kg CO_2eq/day (95% CI)**	0.34 (0.29 to 0.38)	0.18 (0.04 to 0.31)	0.34 (0.23 to 0.45)	-1.48 (-1.79 to -1.18)
**Mean difference in blue WF achieved by switching to meeting guideline**	**l/day (95% CI)**	28.5 (17.4 to 39.8)	10.0 (9.37 to 10.7)	8.23 (7.69 to 8.77)	-22.5 (-22.7 to -22.3)

Table 1: Mean per-capita change in environmental footprints from switching from non-adherence to adherence to food-based EWG recommendations (*from current level of adherence to adherence by all)*

Discussion

Adherence to the Eatwell Guide is currently low among the UK population. Our analysis of three large UK cohort studies suggests that greater adherence is associated with population health benefits, and using data from the nationally-representative National Diet and Nutrition Survey data, we demonstrate that increased EGW adherence is associated with a lower environmental footprint in terms of GHG emissions, although not water use. Adherence to some EWG recommendations would increase environmental footprints in some instances. Taken together these findings suggest broad benefits to public health and the environment of adherence to the EWG and provides evidence to support strengthened national action to improve diets in the UK for the benefit of people and the planet.

Our findings support earlier analysis [16] that UK diets fully compliant with the EWG have lower environmental footprints. Previous studies of the sustainability of UK diets have found that considerable co-benefits to environment and health could be achieved by meeting WHO dietary guidelines [26, 27]; increasing adherence to the EAT Lancet diet [28]; and following a predominantly plant-based diet [12, 15, 29, 30]. While our analysis confirms that reducing consumption of red and processed meat is paramount for lowering environmental footprints of diets, population health benefits showed to be strongly associated with the recommended consumption of fruit and vegetables.

The estimated 7% reduction in mortality and 30% reduction in emissions (or an average absolute reduction of 0.58 tonne GHGe per person per year) through better adherence to the EWG-guidelines is similar in magnitude as compared to other population-level interventions aiming multiple benefits for health and the environment. For example, a study evaluating a future scenario of increased active travel and lower-emission motor vehicles in London [31] estimated a 0.72 tonne reduction in per person GHGe as compared to the business-as-usual scenario, as well as a 10-19% reduction in years of life lost from ischaemic heart disease. A dietary modelling study from the Netherlands estimated impact on GHGe (4-11%) from substituting 35g/d of meat with vegetables, fruit, nuts, seeds, pasta, rice, couscous or fish [32].

A major strength of this study is its use of four large, high-quality data sources for the UK. A number of sensitivity analyses were conducted to test the robustness of the findings to

For peer review only

different assumptions about the causal relationships between variables, and ranges of environmental footprints were used to construct confidence intervals for those relationships. A further strength is the use of empirical rather than modelled diets for the study. Nevertheless, the analyses also contains several weaknesses: among these was the simplification that all diets that met a certain number of recommendations were equally healthy (or unhealthy) regardless of which recommendations were being met, and the assumption that lower consumption of one food group or nutrient could not be compensated by higher consumption of other foods. Low inter-individual variance in diets associated with high adherence to some recommendations combined with relatively low overall intake (for example red and processed meat) may also have resulted in low power to detect diet-health associations [33]. As for all studies measuring dietary intake, methods could be subject to measurement error; in the three datasets considered in this study, however, dietary intake data were collected using different methods, reducing the likelihood of type I errors across all included studies. Data on greenhouse gas emissions were obtained from diverse sources with used different methods and time periods. Data on water footprints were obtained from a single source, but this source used average crop water requirements and yields from the years 1996-2005, and these values may therefore have changed by the time of the UK dietary survey ~15 years later, resulting in some inaccuracies of food water footprints. We attempted to select data on greenhouse gas emissions from surveys with years corresponding to the years of the NDNS, but this was not always possible and therefore the same inaccuracies may affect the greenhouse gas footprints of the diets. Finally, due to data limitations it was not possible to assess both health and environmental footprints of diets within single datasets.

The EWG dietary recommendations are associated with better health outcomes and lower GHG emissions but are substantially different from the “planetary health diet” recently recommended [5], particularly in terms of red and processed meat consumption. Our analysis suggests that considerable dietary shifts are still required in UK dietary habits to meet the EWG recommendations and that additional substantial changes would be needed to meet the more stringent planetary health diet recommendations. A major determinant of such shifts will be food prices [34, 35] and recent analysis has demonstrated that affordability of such diets may vary substantially [36]. Furthermore, it should be noted that an increasing proportion of plant-based foods - that is supplied to the UK - is imported from abroad; shifts in diets towards such foods – and no change in trading strategy - would further increase reliance on foreign production for resilient supply of plant-based foods. Moreover, an increasingly large proportion of these plant-based food imports originates from countries that are highly-vulnerable to climate change (e.g. countries that are predicted to be highly water deficient by 2030). [23]. Care should be taken to avoid that dietary shifts towards EWG-adherence (and hence more plant-based diets) would result in substantial virtual water trade – away from water scarce countries - to supply the UK markets.

A - carefully considered - nationwide shift towards adherence of the EWG will provide an essential step towards sustainable and healthy diets in the UK. Health services including family doctors [37] must play an active role in promoting adherence to the EWG recommendations to their patients and thereby contribute directly to population health and environmental sustainability.

Funding & Role of Funder

This work was supported by the Wellcome Trust, Our Planet Our Health Programme (Sustainable and Health Food Systems - SHEFS [grant number 205200/Z/16/Z] & Livestock, Environment and People - LEAP [grant number 205212/Z/16/Z]); Cancer Research UK [grant

For peer review only - http://bmjopen.bmj.com/site/about/guidelines.xhtml

numbers C8211/A19170; C570/A11692] and the UK Medical Research Council [grant numbers MR/M012190/1; MR/K02700X/1].

The views expressed in this publication are those of the authors and not necessarily those of the Public Health England or the Wellcome Trust. The final version of the report and ultimate decision to submit for publication was determined by the authors.

Conflict of Interest Declaration

The authors declare no conflict of interest

Authors' contributions:

PS - literature search, study design, data analysis, data interpretation, manuscript writing

RG - study design, data analysis, data interpretation, manuscript writing

KP - study design, data analysis, data interpretation, manuscript writing

AK - study design, data analysis, data interpretation, manuscript writing

CA - data analysis, commenting on manuscript

AB - data analysis, commenting on manuscript

TK - study design, commenting on manuscript

VB - study design, commenting on manuscript

AD – study design, commenting on manuscript

Patients and Public Involvement

No patients or members or public were involved in this manuscript, but data used is representative for the general UK public.

Conflict of interest statements

We have no conflicts of interest to declare.

Transparency declaration

The lead author affirms that this manuscript is an honest, accurate, and transparent account of the study being reported; that no important aspects of the study have been omitted; and

For peer review only

that any discrepancies from the study as planned (and, if relevant, registered) have been explained.

Acknowledgements

The authors thank the women who have participated in the Million Women Study as well as the staff from the participating NHS breast screening centres. We also thank NHS Digital in England and the Information Services Division, NHS Scotland for linkage to data on cancers and deaths, and Public Health England for data based on information collected and quality assured by the Public Health England National Cancer Registration and Analysis Service, for which access was facilitated by the Office for Data Release. Data for this study include information collected and provided by the Office for National Statistics. Those who carried out the original collection and analysis of the data bear no responsibility for their further analysis or interpretation.

Data access policies for the Million Women Study are available via the study website [http://www.millionwomenstudy.org/]

We thank all participants in the EPIC-Oxford cohort for their invaluable contribution.

This research has been conducted using the UK Biobank Resource under application number 24494. All bona fide researchers can apply to use the UK Biobank resource for health related research that is in the public interest [https://www.ukbiobank.ac.uk/register-apply/]. We thank all participants, researchers and support staff who make the study possible.

We thank Paul Appleby and Aurora Perez-Cornago (Cancer Epidemiology Unit, Nuffield Department of Population Health, University of Oxford) for their valuable contribution to data preparation.

For peer review only

References

1. World Health Organization. *Malnutrition Fact Sheet - Key Facts*. 2018 February 2018 [cited 2019 November]; Available from: https://www.who.int/news-room/fact-sheets/detail/malnutrition.
2. World Health Organization. *WHO Nutrition Topics - Micronutrient Deficiencies*. 2019 [cited 2019 November]; Available from: https://www.who.int/nutrition/topics/ida/en/.
3. Mbow, H.-O.P., et al., *Special Report on climate change, desertification, land degradation, sustainable land management, food security, and greenhouse gas fluxes in terrestrial ecosystems (SR2).* 2017.
4. Food and Agriculte Organization of the United Nations, *Water for Sustainable Food and Agriculture - A report produced for the G20 Presidency of Germany*. 2017, FAO: Rome.
5. Willett, W., et al., *Food in the Anthropocene: the EAT–Lancet Commission on healthy diets from sustainable food systems.* The Lancet, 2019. **393**(10170): p. 447-492.
6. Fischer, C.G. and T. Garnett, *Plates, pyramids, and planets: developments in national healthy and sustainable dietary guidelines: a state of play assessment*. 2016: Food and Agriculture Organization of the United Nations.
7. Brazil, M.o.H.o., *Dietary guidelines for the Brazilian population*. 2014, Ministry of Health of Brazil Brasília-DF (Brazil).
8. Public Health England. *The Eatwell Guide - Helping you eat a healthy, balanced diet*. 2018.
9. Aune, D., et al., *Fruit and vegetable intake and the risk of cardiovascular disease, total cancer and all-cause mortality—a systematic review and dose-response meta-analysis of prospective studies.* International journal of epidemiology, 2017. **46**(3): p. 1029-1056.
10. Levy, L. and A. Tedstone. *UK dietary policy for the prevention of cardiovascular disease*. in *Healthcare*. 2017. Multidisciplinary Digital Publishing Institute.
11. Cobiac, L.J., et al., *The Eatwell guide: modelling the health implications of incorporating new sugar and fibre guidelines.* PLoS One, 2016. **11**(12).
12. Aston, L.M., J.N. Smith, and J.W. Powles, *Impact of a reduced red and processed meat dietary pattern on disease risks and greenhouse gas emissions in the UK: a modelling study.* BMJ open, 2012. **2**(5): p. e001072.
13. Harris, F., et al., *The Water Footprint of Diets: A Global Systematic Review and Meta-analysis.* Advances in Nutrition, 2019.
14. Poore, J. and T. Nemecek, *Reducing food's environmental impacts through producers and consumers.* Science, 2018. **360**(6392): p. 987-992.
15. Scarborough, P., et al., *Dietary greenhouse gas emissions of meat-eaters, fish-eaters, vegetarians and vegans in the UK.* Climatic change, 2014. **125**(2): p. 179-192.
16. Trust, C., *The Eatwell Guide: A More Sustainable Diet*. 2016, The Carbon Trust London, UK.
17. Davey, G.K., et al., *EPIC–Oxford:lifestyle characteristics and nutrient intakes in a cohort of 33 883 meat-eaters and 31 546 non meat-eaters in the UK.* Public Health Nutrition, 2007. **6**(3): p. 259-268.
18. Collins, R., *What makes UK Biobank special?* Lancet, 2012. **379**(9822): p. 1173-1174.
19. Green, J., et al., *Cohort profile: the million women study.* International journal of epidemiology, 2018. **48**(1): p. 28-29e.
20. Agency, P.H.E.t.U.F.S., *National Diet and Nutrition Survey (NDNS) wave 5-9 (2012-2017)*. 2019.
21. Scarborough, P., et al., *Eatwell Guide: modelling the dietary and cost implications of incorporating new sugar and fibre guidelines.* BMJ open, 2016. **6**(12): p. e013182.
22. Public Health England & the UK Food Standards Agency, *Appendix A: Dietary data collection and editing. In: Results from Years 7-8 (combined) of the Rolling Programme (2014/15 – 2015/16)*. 2018.
23. Food and Agriculte Organization of the United Nations, *FAOSTAT Statistical Database.* 2019: Rome.

For peer review only - http://bmjopen.bmj.com/site/about/guidelines.xhtml

24. Mekonnen, M.M. and A.Y. Hoekstra, *The green, blue and grey water footprint of crops and derived crop products.* Hydrology and Earth System Sciences, 2011. **15**(5): p. 1577-1600.
25. Department of Health GB, *41 Dietary reference values for food energy and nutrients for the United Kingdom : Report of the Panel on Dietary Reference Values of the Committee on Medical Aspects of Food Policy*. 1991, London: H.M.S.O.
26. Milner, J., et al., *Health effects of adopting low greenhouse gas emission diets in the UK.* BMJ open, 2015. **5**(4): p. e007364.
27. Green, R., et al., *The potential to reduce greenhouse gas emissions in the UK through healthy and realistic dietary change.* Climatic Change, 2015. **129**(1-2): p. 253-265.
28. Knuppel, A., et al., *EAT-Lancet score and major health outcomes: the EPIC-Oxford study.* The Lancet, 2019.
29. Cobiac, L.J. and P. Scarborough, *Modelling the health co-benefits of sustainable diets in the UK, France, Finland, Italy and Sweden.* European journal of clinical nutrition, 2019. **73**(4): p. 624.
30. Scarborough, P., et al., *Modelling the health impact of environmentally sustainable dietary scenarios in the UK.* European journal of clinical nutrition, 2012. **66**(6): p. 710.
31. Woodcock, J., et al., *Public health benefits of strategies to reduce greenhouse-gas emissions: urban land transport.* The Lancet, 2009. **374**(9705): p. 1930-1943.
32. Biesbroek, S., et al., *Reducing our environmental footprint and improving our health: greenhouse gas emission and land use of usual diet and mortality in EPIC-NL: a prospective cohort study.* Environmental Health, 2014. **13**(1): p. 27.
33. Schwingshackl, L., et al., *Food groups and risk of all-cause mortality: a systematic review and meta-analysis of prospective studies.* The American Journal of Clinical Nutrition, 2017. **105**(6): p. 1462-1473.
34. Green, R., et al., *The effect of rising food prices on food consumption: systematic review with meta-regression.* Bmj, 2013. **346**: p. f3703.
35. Scheelbeek, P.F., et al., *Potential impact on prevalence of obesity in the UK of a 20% price increase in high sugar snacks: modelling study.* bmj, 2019. **366**: p. l4786.
36. Hirvonen, K., et al., *Affordability of the EAT–Lancet reference diet: a global analysis.* The Lancet Global Health, 2019.
37. WONCA Working Party on the Environment, Planetary Health Alliance, and Clinicians for Planetary Health Working Group, *Declaration Calling for Family Doctors of the World to Act on Planetary Health*. 2019.

For peer review only

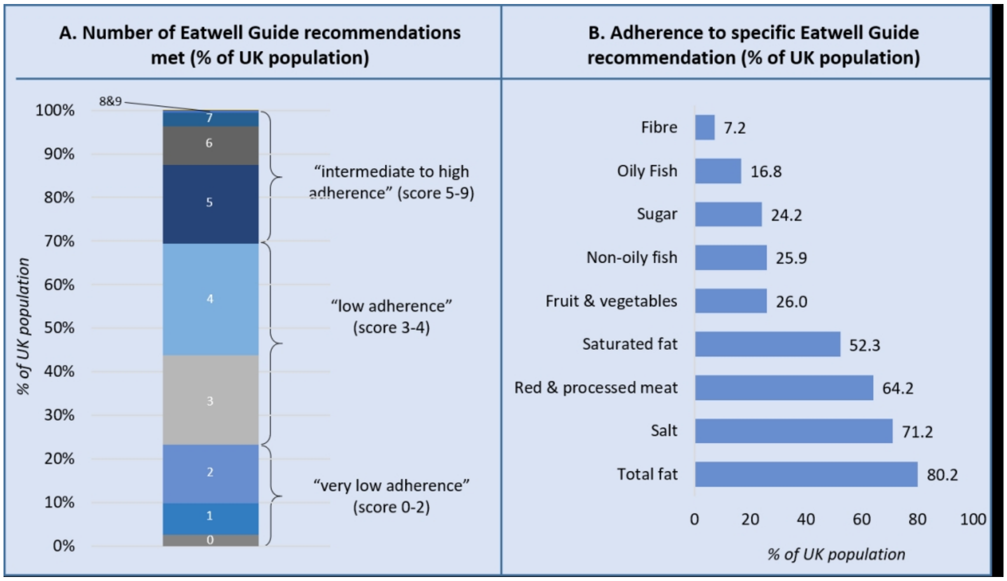

Figure 1: Adherence to the Eatwell Guide recommendations by the UK population - based on data from wave 5-9 of the National Dietary and Nutrition Survey (NDNS): A) total number of recommendations met by % of UK population; B) adherence to specific recommendations

236x135mm (150 x 150 DPI)

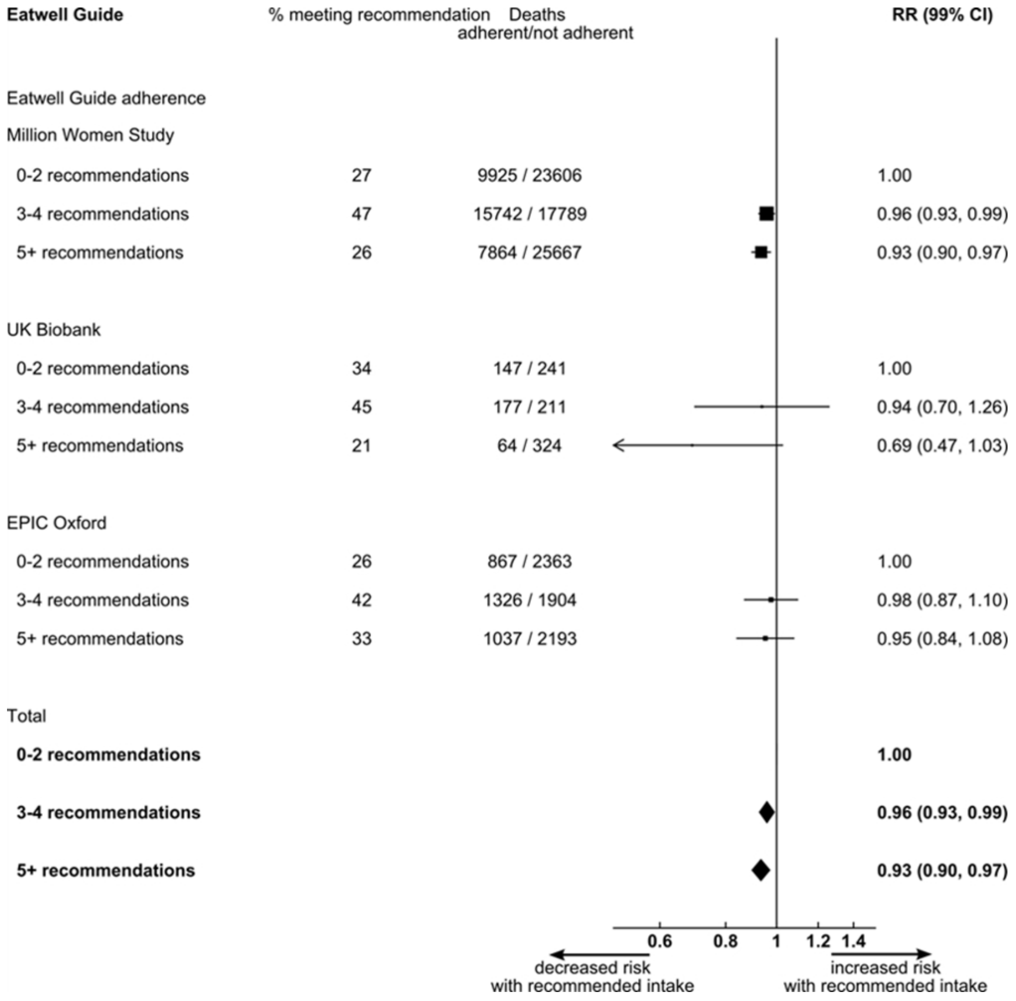

Figure 2: Forest plot showing the study specific (MWS, UKB & EPIC-Ox) and pooled mortality risk ratios comparing very poor adherence to EWG rocmmendations (score 0-2) with poor adherence (score 3-4) and intermediate to high adherence (score 5-9).

153x151mm (149 x 149 DPI)

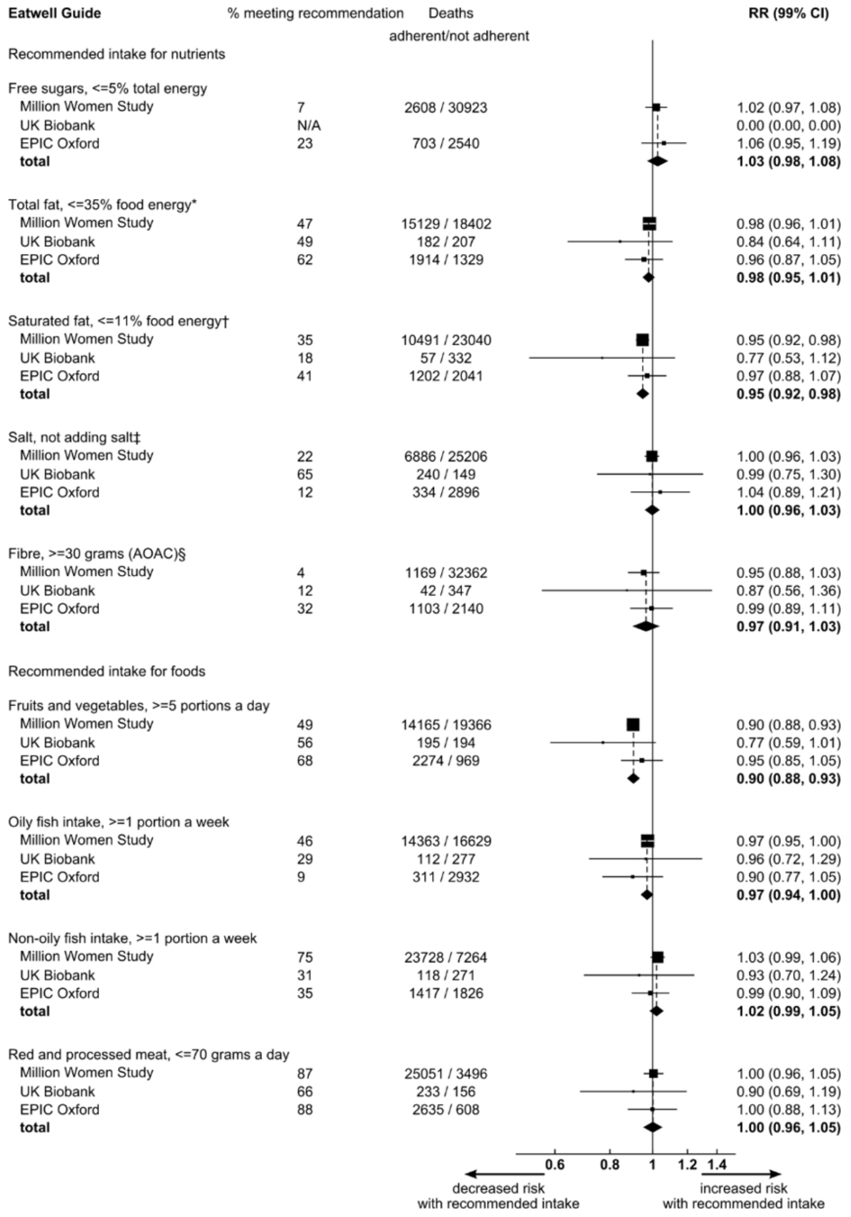

Figure 3: Mortality risk ratios for the association between adhering to specific EWG recommendations and total mortality. * Recommendation was based on food energy and was therefore adapted to ≥47% of total energy [21]. *Adapted to <=33% of total energy [21]. †Adapted to <=10% of total energy [21]. ‡Information on salt intake was ascertained from the variable 'Never adding salt to food at the table or cooking' in the MWS and in the EPIC-Oxf study; and from the variable 'Not reporting having added salt to food (excluding during cooking)' in any of the WEBQs included in the UKB. §Fibre intake in the study was determined using the Englyst method and the recommendation was therefore adapted to >=22.6g/d of Englyst fibre.

143x206mm (150 x 150 DPI)

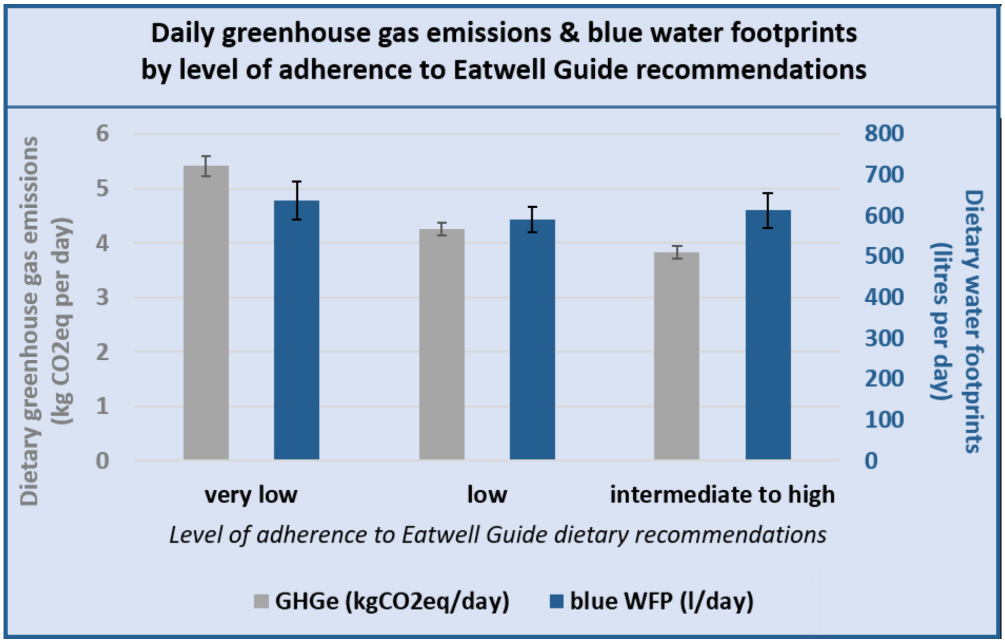

Figure 4: Average daily greenhouse gas emissions in kg CO_2eq and average daily dietary water footprints comparing diets with very low (score 0-2), low (score 3-4) and intermediate to high adherence (score 5-9) to the Eatwell Guide dietary guidelines.

160x101mm (150 x 150 DPI)

Appendixes

To: ***Sustainable Food Systems and Diets in the UK: The Eatwell Guide as a blueprint for healthy and sustainable diets in the UK.***

For peer review only

Appendix 1 - Basic study descriptions for each cohort

Million Women Study

We used data from 464,078 participants of the Million Women Study. Participants were recruited from women (mean age 56 years) invited to the National Health service (NHS) breast cancer screening programme in England and Scotland between 1996 and 2001 [1]. Dietary intake was collected after an average of 3.3 years post recruitment using 130 semi-quantitative questions that were validated against a 7-day diet diary [2]. Nutrients were estimated by multiplying the frequency of consumption by portion size and nutrient composition of that item [3].

Total mortality was determined using death records obtained through linkage to centrally held NHS records. The Million Women Study protocol was approved by the Oxford and Anglia Multi-Centre Research Ethics committee. All participants provided written informed consent.

EPIC Oxford

We used data from 40,030 men and women (mean age 43 years) recruited throughout the UK (between 1993-2001) into the European Prospective Investigation into Cancer and Nutrition (EPIC)-Oxford cohort [4]. Dietary intake was collected using a validated 130-item semi-quantitative Food frequency questionnaire [5]. To estimate nutrient intakes, we multiplied frequencies of consumption by portion size and nutrient composition.

Total mortality was ascertained using death record linkage with the NHS Central register.

The EPIC-Oxford study protocol was approved by a Multi-Centre Research Ethics Committee (Scotland A Research Ethics Committee). All participants provided written informed consent.

UK Biobank

We used data from 53,614 middle-aged adults (mean age 56 years) participating in the UK Biobank who were recruited across the UK between 2006-2010 [6]. These were a subsample, that had completed a minimum of three 24-hour dietary recall questionnaires (the Oxford WebQ) [7]. Food and beverage frequency data, standard portion sizes and nutrient composition by item were multiplied to obtain nutrient intakes per day [8].

We linked participant data to the NHS Central register to obtain mortality information.

The UK Biobank was approved by the National Information Governance Board for Health and Social Care and the NHS North West Multi-Centre Research Ethics Committee. All participants provided written informed consent.

For peer review only

References

1. Green, J., et al., *Cohort profile: the million women study.* International journal of epidemiology, 2018. **48**(1): p. 28-29e.
2. Roddam, A.W., et al., *Reproducibility of a short semi-quantitative food group questionnaire and its performance in estimating nutrient intake compared with a 7-day diet diary in the Million Women Study.* Public Health Nutr, 2005. **8**(2): p. 201-13.
3. Key, T.J., et al., *Foods, macronutrients and breast cancer risk in postmenopausal women: a large UK cohort.* Int J Epidemiol, 2018: p. dyy238-dyy238.
4. Davey, G.K., et al., *EPIC–Oxford:lifestyle characteristics and nutrient intakes in a cohort of 33 883 meat-eaters and 31 546 non meat-eaters in the UK.* Public Health Nutrition, 2007. **6**(3): p. 259-268.
5. Bingham, S.A., et al., *Validation of dietary assessment methods in the UK arm of EPIC using weighed records, and 24-hour urinary nitrogen and potassium and serum vitamin C and carotenoids as biomarkers.* Int J Epidemiol, 1997. **26 Suppl 1**: p. S137-51.
6. Collins, R., *What makes UK Biobank special?* Lancet, 2012. **379**(9822): p. 1173-1174.
7. Greenwood, D.C., et al., *Validation of the Oxford WebQ online 24-hour dietary questionnaire using biomarkers.* Am J Epidemiol, 2019.
8. Bradbury, K.E., et al., *Dietary assessment in UK Biobank: an evaluation of the performance of the touchscreen dietary questionnaire.* J Nutr Sci, 2018. **7**: p. e6.

For peer review only

Appendix 2 – Eatwell Guide recommendations by age and sex

	5-6 years		7-10 years		11-14 years		15-18 years		19-64 years		65-74 years		75+ years	
**Food group**	Male	Female	Male	Female	Male	Female	Male	Female	Male	Female	Male	Female	Male	Female
Fruit & Vegetables (g/day)	400*	400g*	400g*	400g*	400g*	400g*	400g*	400g*	400g*	400g*	400g*	400g*	400g*	400g*
Oily Fish (g/day)	20g	20g	20g	20g	20g	20g	20g	20g	20g	20g	20g	20g	20g	20g
Other Fish (g/day)	20g	20g	20g	20g	20g	20g	20g	20g	20g	20g	20g	20g	20g	20g
Red and processed meat (g/day)	70	70	70	70	70	70	70	70	70	70	70	70	70	70
Fibre (g/day)	20**	20**	20**	20**	25**	25**	30**	30**	30**	30**	30**	30**	30**	30**
Salt (g/day)	3**	3**	5**	5**	6	6	6	6	6	6	6	6	6	6
Free Sugar (g/day)	19	19	24	24	30	30	30	30	30	30	30	30	30	30
Saturated fat (g/day)	18**	17 **	22**	20	30	20	30	20	30	20	30	20	30	20
Total fat (g/day)	58**	54**	71**	66**	97**	78**	97**	78**	97**	78**	91**	74**	89**	72**

**30g of dried fruit, max 150ml fruit juice or smoothie, and max 80g beans considered as one portion*

***Figures from PHE Government Dietary Recommendations document, derived from SACN. All other figures from the UK Eatwell Guide.*

Dietary recommendations of the Eatwell Guide

	Dietary recommendation and constraints
**Nutrients**	
Energy	2250 kcal (9414 MJ)*
Carbohydrates	≥50% of food energy
Free sugars	≤5% food energy
Fat	≤35% food energy
Saturated fat	≤11% food energy
Protein	≥14.5 & ≤15.5% of energy
Salt	≤ 6g/2363 mg/d sodium
Fibre (AOAC)	≥30g/d
**Foods**	
Fruits and vegetables†	≥5 portions a day
Fish	≥ 2 portions (2*140g) a week, one of which should be oily
Red and processed meat	≤70g/day

Table adapted from Public Health England (2016), Table 2.
**Energy intake recommendation assumes mixed population average.*
†Fruit and vegetable intake includes a maximum of: 1 portion of juice (from fruit / vegetable juice or that in a smoothie); 1 portion of beans; (portion sizes: 30g dried fruit; combined total of 150ml of fruit and / or vegetable juice and / or smoothie; 80g all other fruits & vegetables).

For peer review only

Appendix 3 – Cohort specific adjustments and cut-off values

Million Women Study

Associations were stratified by region and adjusted for smoking (never smoked, past smoker, current smoker <10 cigarettes smoked per day, current smoker 10-19 cigarettes smoked per day, current smoker ≥20 cigarettes smoked per day, current smoker and unknown number of cigarettes smoked per day), deprivation (tertiles), alcohol (0, 1-6, 7-14, ≥15 drinks per week), height cm (<155 ,155-164, ≥165), BMI kg/m^2 (<20, 20-24, 25-29, ≥30), strenuous exercise (<1 per week,≥1 per week), Hormone replacement therapy (HRT) use (never, past, current), educational attainment (none, technical/secondary/tertiary), self-reported hypertension (yes, no), and energy intake (quintiles).

EPIC Oxford

Associations were stratified by sex, region, and method of recruitment and adjusted for smoking (never smoked, past smoker, current smoker <10 cigarettes smoked per day, current smoker 10-19 cigarettes smoked per day, current smoker ≥20 cigarettes smoked per day, current smoker and unknown number of cigarettes smoked per day), deprivation (tertiles, unknown), alcohol intake (<1, 1-7 (ref.), 8-15, ≥16 grams per day, unknown), height cm (sex-specific tertiles, unknown), BMI kg/m^2(<20, 20-24, 25-29, ≥30), physical activity (inactive, low, moderate, high activity, unknown), HRT use ever (yes, no, unknown), educational attainment (national examination at age 16, national examination at ages 17-18, vocational qualification, , degree, unknown), self-reported high blood pressure (no, yes, unknown), and energy intake (sex-specific quintiles)

UK Biobank

Associations were stratified by sex and region and adjusted for smoking (never smoked, past smoker, current smoker <10 cigarettes smoked per day, current smoker 10-19 cigarettes smoked per day, current smoker ≥20 cigarettes smoked per day, current smoker and unknown number of cigarettes smoked per day), deprivation (tertiles, unknown), alcohol intake (none, <1, 1-<10, 10-<20, ≥20 grams per day, unknown), height cm (sex-specific tertiles, unknown), BMI kg/m^2 (<20, 20-24, 25-29, ≥30), physical activity (<10 excess METs, 10-<50 excess METs, ≥50 excess METs, unknown), qualification (national examination at age 16, national examination at ages 17-18, vocational qualification, college or university degree, other, unknown), HRT (never, past, current, unknown), self-reported high blood pressure (no, yes, unknown), and energy intake (sex-specific quintiles)

For peer review only

Appendix 4 – Results sensitivity analysis mortality RR

Eatwell Guide	% meeting recommendation	Deaths adherent/not adherent	RR (99% CI)
Recommended intake for nutrients			
Free sugars, <=5% total energy			
Million Women Study	7	2608 / 30923	1.09 (1.03, 1.15)
UK Biobank			
EPIC Oxford	23	703 / 2540	1.04 (0.93, 1.17)
**total**			**1.08 (1.03, 1.13)**
Total fat, <=35% food energy?			
Million Women Study	47	15129 / 18402	0.92 (0.89, 0.95)
UK Biobank	N/A	182 / 207	0.81 (0.62, 1.07)
EPIC Oxford	62	1914 / 1329	0.92 (0.84, 1.01)
**total**			**0.92 (0.89, 0.95)**
Saturated fat, <=11% food energy?			
Million Women Study	35	10491 / 23040	0.88 (0.85, 0.91)
UK Biobank	18	57 / 332	0.75 (0.52, 1.10)
EPIC Oxford	41	1202 / 2041	0.94 (0.85, 1.04)
**total**			**0.89 (0.86, 0.91)**
Salt, not adding saltô			
Million Women Study	22	6886 / 25206	0.95 (0.91, 0.98)
UK Biobank	65	240 / 149	0.94 (0.72, 1.24)
EPIC Oxford	12	334 / 2896	1.01 (0.87, 1.18)
**total**			**0.95 (0.92, 0.98)**
Fibre, >=30g (AOAC)			
Million Women Study	4	1169 / 32362	0.89 (0.82, 0.96)
UK Biobank	12	42 / 347	0.86 (0.55, 1.34)
EPIC Oxford	32	1103 / 2140	0.96 (0.86, 1.06)
**total**			**0.91 (0.85, 0.97)**
Recommended intake for foods			
Fruits and vegetables, >=5 portions a day			
Million Women Study	49	14165 / 19366	0.82 (0.80, 0.85)
UK Biobank	56	195 / 194	0.74 (0.57, 0.98)
EPIC Oxford	68	2274 / 969	0.88 (0.80, 0.98)
**total**			**0.83 (0.80, 0.85)**
Oily fish intake, >=1 portion a week			
Million Women Study	46	14363 / 16629	0.93 (0.91, 0.96)
UK Biobank	29	112 / 277	0.95 (0.71, 1.28)
EPIC Oxford	9	311 / 2932	0.90 (0.77, 1.05)
**total**			**0.93 (0.91, 0.96)**
Non-oily fish intake, >=1 portion a week			
Million Women Study	75	23728 / 7264	1.03 (1.00, 1.07)
UK Biobank	31	118 / 271	0.93 (0.70, 1.24)
EPIC Oxford	35	1417 / 1826	1.00 (0.91, 1.10)
**total**			**1.03 (0.99, 1.06)**
Red and processed meat, <=70g/day			
Million Women Study	87	25051 / 3496	0.99 (0.94, 1.04)
UK Biobank	66	233 / 156	0.88 (0.66, 1.15)
EPIC Oxford	88	2635 / 608	0.95 (0.84, 1.08)
**total**			**0.98 (0.94, 1.03)**

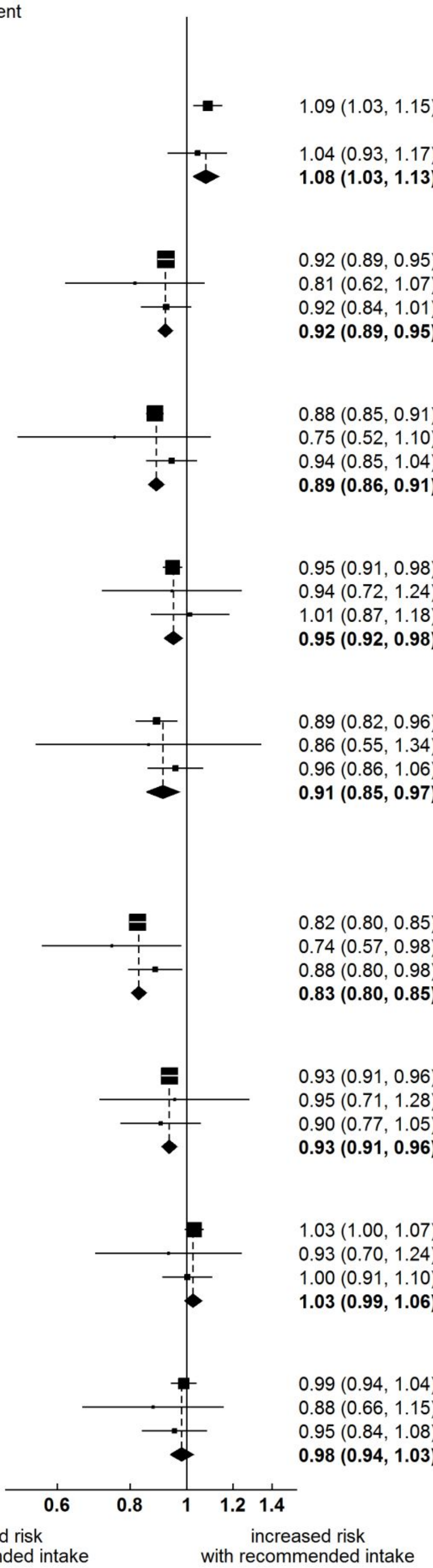

Figure S 1 Risk ratios for the association between some recommendations of the Eatwell Guide and total mortality <u>without adjustment for smoking</u>

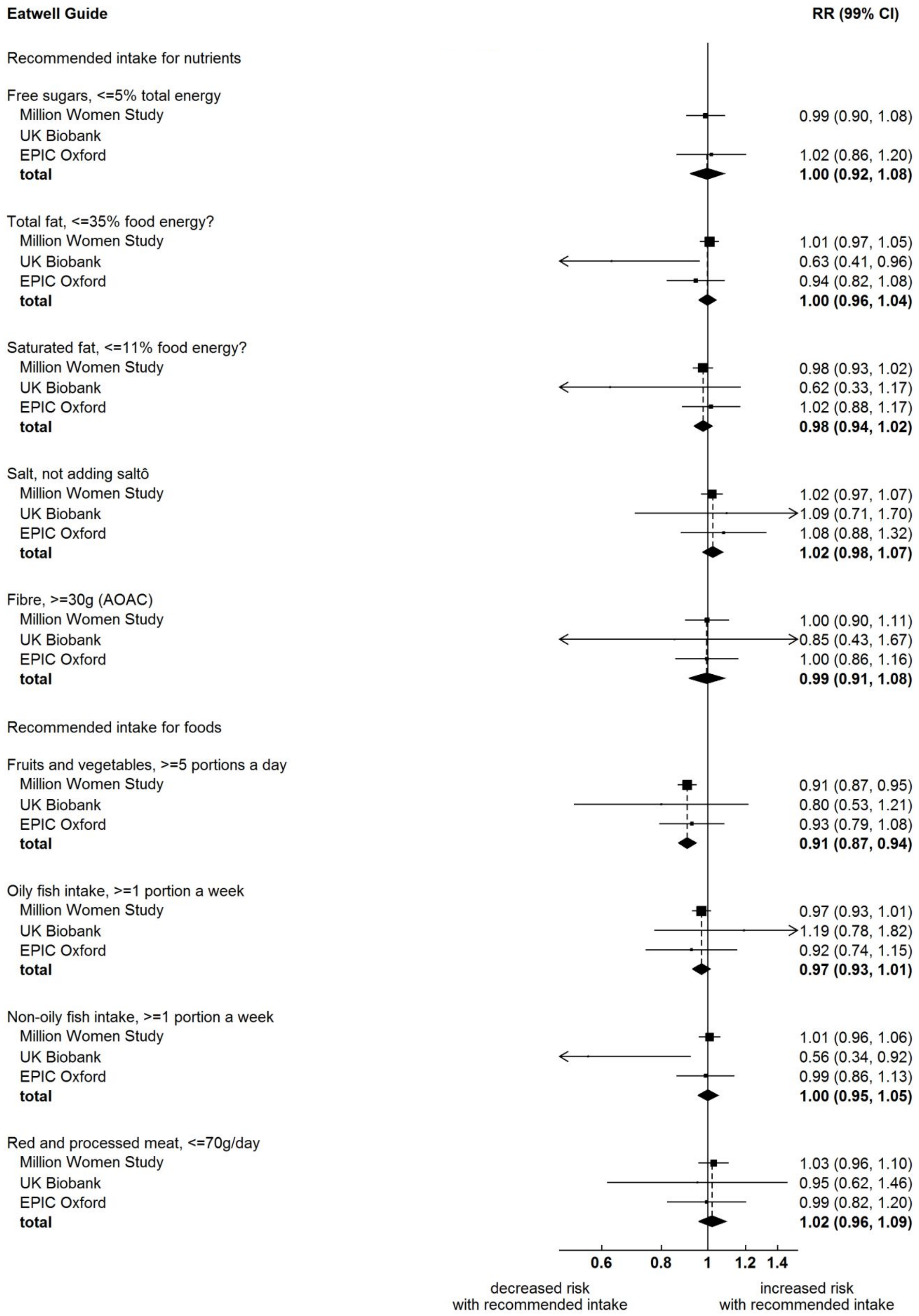

Figure S 2 Risk ratios for the association between some recommendations of the Eatwell Guide and total mortality in never smokers

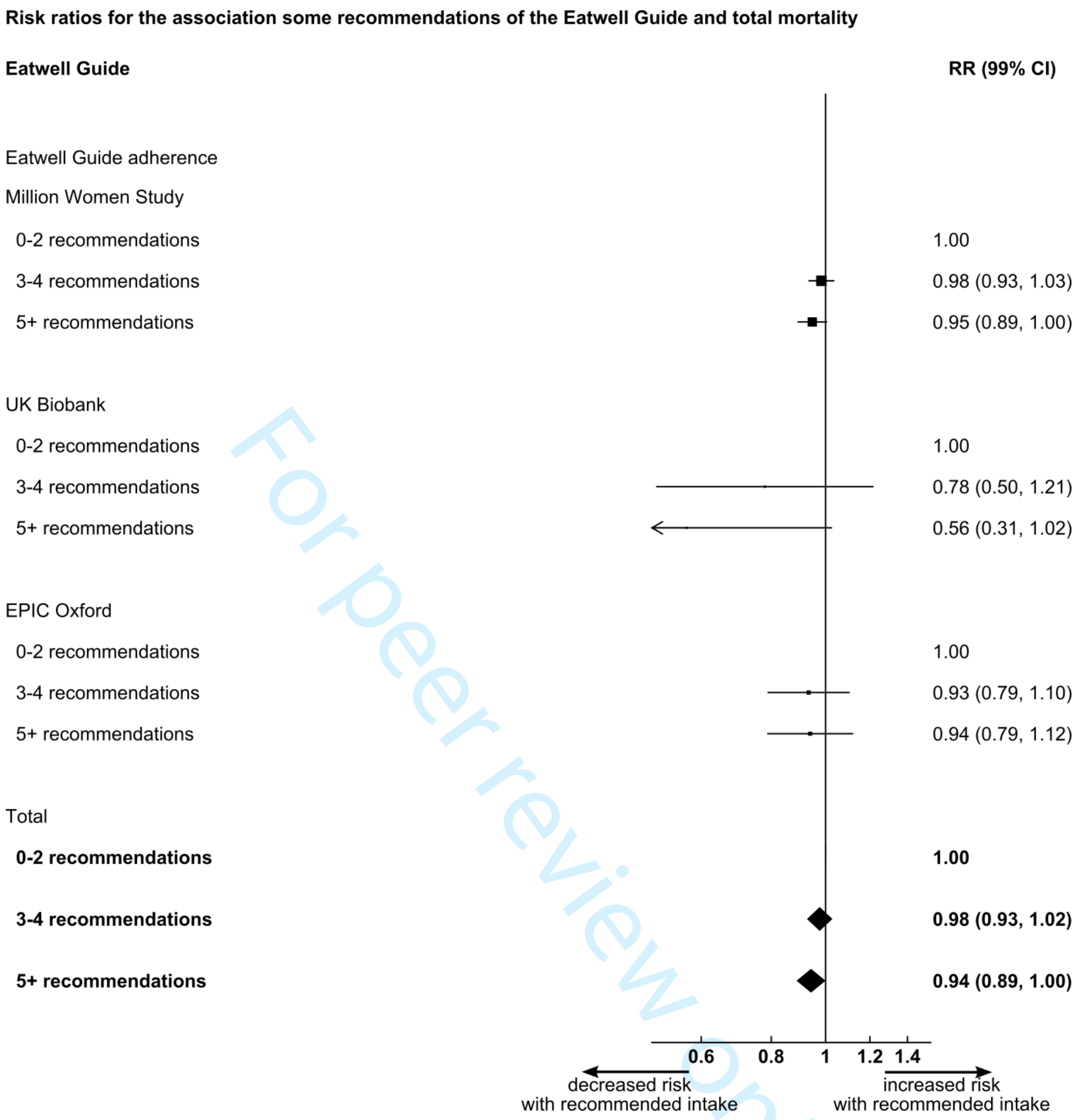

Figure S 1 Risk ratios for the association between the Eatwell Guide three level score and total mortality in never smokers

For peer review only

Appendix 5 – Detailed GHG emission and WFP calculations

Data from NDNS were matched to food-specific GHG emissions and blue water footprint estimates from previous studies using 173 SubFoodGroups available in the NDNS dataset. Where SubFoodGroups contained a number of foods with differing environmental footprints, weighted means according to consumption were used, and similarly where SubFoodGroups were composite foods including many ingredients (e.g. meat pies) recipe data were used to estimate the mean proportions of ingredients contained within the composite food. Recipe data were obtained from Sainsbury's (www.sainsbury.co.uk). Composite food and weighting data are available on request.

A number of additional SubFoodGroup categories were created in order to distinguish between categories where environmental footprints would be expected to differ greatly. These were:

- SubFoodGroup **6A** (wheat based breakfast cereals), **6B** (chocolate breakfast cereals), **6C** (oat based breakfast cereals), **6D** (maize based breakfast cereals), and **6E** (rice based breakfast cereals) were added in MainFoodGroup 6 (other breakfast cereals)
- SubFoodGroup **5A** (wheat based high fibre breakfast cereals), **5B** (chocolate high fibre breakfast cereals), **5C** (oat based high fibre breakfast cereals), and **5D** (rice based high fibre breakfast cereals) were added in MainFoodGroup 5 (high fibre breakfast cereals) and SubFoodGroup 5R was renamed (other high fibre breakfast cereals)
- SubFoodGroup **7C** (biscuits chocolate retail) was added in MainFoodGroup 7 (biscuits).
- SubFoodGroup **8F** (cakes chocolate) was added in MainFoodGroup 8 (buns cakes pastries and fruit pies)
- SubFoodGroups **13C** (non-dairy cream), **13D** (almond milk), **13E** (soya milk), **13F** (other non-dairy milk) and **13G** (chocolate milk) were added in MainFoodGroup 13 (other milk and cream) – Elmlea is not included in non dairy cream as it contains buttermilk
- SubFoodGroup **14C** (dairy free cheese) was added in MainFoodGroup 14 (cheese)
- SubFoodGroup **15E** (non dairy desserts) was added in MainFoodGroup 15 (yoghurt fromage frais and dairy desserts)
- SubFoodGroup **19B** (less than 1% dairy low fat spread) was added in MainFoodGroup 19 (low fat spread)
- SubFoodGroup **20A** (block margarine) was included in the dairy-free categories
- SubFoodGroup **20C** (other cooking fats and oils not PUFA) was renamed (other vegetable fats and oils) and a new SubFoodGroup **20B** (animal fats) was added in MainFoodGroup 20 (other margarine fats and oils)
- SubFoodGroup **21C** (less than 1% dairy reduced fat spread) was added in MainFoodGroup 21 (reduced fat spread)
- SubFoodGroup 37C (baked beans with sausages), moved to SubFoodGroup **25A** (manufactured pork products including ready meals) and categorised into MainFoodGroup 25 (pork products).
- SubFoodGroup **53A** (non dairy ice cream) was added in MainFoodGroup 53 (ice cream)

For peer review only

Codes 13A (infant formula), 54B (evening primrose oil and other plant oils), 54D (folic acid), 54E (iron only or with vitamin C), 54F (calcium only or with vitamin D), 54G (vitamins (two or more including multivitamins), no minerals), 54H (minerals (two or more including multimins), no vitamins), 54I (vitamins and minerals (including multivits and minerals)), 54J (non-nutrient supplements including herbal), 54K (other nutrient supplements), 54L (vitamin C only), 54M (other single vitamins or minerals), 54M (cod liver oil and other fish oils), 54P (multivitamins and/or minerals with omega 3),

SubFood GroupCode	Food Group Name	Mean Greenhouse Gas emissions (kg) per kg food	Mean blue water footprint (litres) per kg food
1C	Pizza	3.51	369
1D	Pasta manufactured products and ready meals	1.00	0
1E	Other pasta including homemade dishes	1.00	0
1F	Rice manufactured products and ready meals	3.13	1071
1G	Other rice including homemade dishes	3.13	1071
1R	Other cereals	1.18	103
2R	White bread (not high fibre, not multiseed)	0.97	0
3R	Wholemeal bread	0.97	0
4R	Other bread	0.97	0
5A	Wheat based high fibre breakfast cereals	1.40	229
5B	Chocolate high fibre breakfast cereals	2.68	62
5C	Oat based high fibre breakfast cereals	1.41	109
5R	Other high fibre breakfast cereals	1.27	2
6A	Wheat based breakfast cereals	1.40	0
6B	Chocolate breakfast cereals	3.03	779
6C	Oat based breakfast cereals	1.41	109
6D	Maize based breakfast cereals	2.64	124
6E	Rice based breakfast cereals	2.85	1009
6R	Other breakfast cereals (not high fibre)	1.27	2
7A	Biscuits manufactured / retail	1.80	143
7B	Biscuits homemade	2.30	201
7C	Biscuits chocolate retail	8.14	135
8B	Fruit pies manufactured	0.95	86
8C	Fruit pies homemade	1.11	91
8D	Buns cakes and pastries manufactured	1.08	66
8E	Buns cakes and pastries homemade	3.31	34
8F	Cakes chocolate	3.11	126
9C	Cereal based milk puddings manufactured	2.00	170
9D	Cereal based milk puddings homemade	2.00	170
9E	Sponge puddings manufactured	1.79	17
9F	Sponge puddings homemade	1.79	17
9G	Other cereal based puddings manufactured	4.00	26
9H	Other cereal based puddings homemade	0.08	1
10R	Whole milk	1.53	28
11R	Semi skimmed milk	1.53	24

For peer review only

12R	Skimmed milk	1.53	24
13B	Cream (including imitation cream)	4.89	44
13C	Non dairy cream alternative	2.64	6
13D	Almond milk	0.99	73
13E	Soya milk	0.88	2
13F	Other non-dairy milk	2.65	57
13G	Chocolate milk	1.53	24
13R	Other milk	1.80	109
14A	Cottage cheese	15.00	132
14B	Cheddar cheese	8.87	132
14C	Dairy free cheese alternative	1.76	4
14R	Other cheese	8.87	59
15B	Yoghurt	2.00	31
15C	Fromage frais and dairy desserts	2.00	27
15D	Dairy desserts homemade	1.32	81
15E	Non dairy desserts	2.05	94
16C	Manufactured egg products including ready meals	3.51	4
16D	Other eggs and egg dishes including homemade	4.70	39
17R	Butter	9.00	194
18B	Polyunsaturated oils	3.59	235
19A	Polyunsaturated low fat spread	4.19	127
19B	Less than 1% dairy low fat spread	4.19	144
19R	Low fat spread not polyunsaturated	3.95	127
20A	Block margarine	4.19	144
20B	Animal fats	14.31	162
20C	Other vegetable fats and oils	4.65	853
21A	Reduced fat spread (polyunsaturated)	4.35	146
21B	Reduced fat spread (not polyunsaturated)	4.35	146
21C	Less than 1% dairy reduced fat spread	4.19	144
22A	Ready meals / meal centres based on bacon and ham	7.47	304
22B	Other bacon and ham including homemade dishes	10.70	321
23A	Manufactured beef products including ready meals	10.40	127
23B	Other beef and veal including homemade recipe dishes	16.50	205
24A	Manufactured lamb products including ready meals	30.48	275
24B	Other lamb including homemade recipe dishes	50.00	446
25A	Manufactured pork products including ready meals	8.85	321
25B	Other pork including homemade recipe dishes	10.00	293
26A	Manufactured coated chicken / turkey products	3.12	80
27A	Manufactured chicken products including ready meals	3.50	38
27B	Other chicken / turkey including homemade recipe dishes	3.50	38
28R	Liver and dishes	8.85	59

For peer review only

29R	Burgers and kebabs purchased	34.80	252
30A	Ready meals based on sausages	4.80	139
30B	Other sausages including homemade dishes	8.85	321
31A	Manufactured meat pies and pastries	8.63	209
31B	Homemade meat pies and pastries	13.96	251
32A	Other meat products manufactured including ready meals	11.50	594
32B	Other meat including homemade recipe dishes	2.84	37
33R	White fish coated or fried	3.36	0
34C	Manufactured white fish products including ready meals	4.55	0
34D	Other white fish including homemade dishes	4.55	0
34E	Manufactured shellfish products including ready meals	24.00	142
34F	Other shellfish including homemade dishes	24.00	142
34G	Manufactured canned tuna products including ready meals	4.55	0
34H	Other canned tuna including homemade dishes	4.55	0
35A	Manufactured oily fish products including ready meals	4.55	256
35B	Other oily fish including homemade dishes	4.55	256
36A	Carrots raw	1.28	0
36B	Salad and other raw vegetables	0.68	16
36C	Tomatoes raw	0.96	36
37A	Peas not raw	2.55	13
37B	Green beans not raw	0.50	40
37C	Baked beans	2.15	428
37D	Leafy green vegetables not raw	0.73	23
37E	Carrots not raw	1.28	0
37F	Tomatoes not raw	0.96	36
37I	Beans and pulses including ready meals and homemade dishes	1.51	21
37K	Meat alternatives including ready meals and homemade dishes	3.60	200
37L	Other manufactured vegetable products including ready meals	1.60	7
37M	Other vegetables including homemade dishes	0.58	39
38A	Chips purchased including take away	1.45	30
38C	Other manufactured potato products fried / baked	1.46	17
38D	Other fried / roast potatoes including homemade dishes	3.08	17
39A	Other potato products and dishes manufactured	1.20	46
39B	Other potatoes including homemade dishes	1.20	19
40A	Apples and pears not canned	0.70	52
40B	Citrus fruit not canned	0.40	93
40C	Bananas	0.90	49
40D	Canned fruit in juice	1.32	218
40E	Canned fruit in syrup	1.32	218
40R	Other fruit not canned	1.63	82

For peer review only

41A	Sugar	0.32	1
41B	Preserves	2.96	206
41R	Sweet spreads fillings and icing	7.14	269
42R	Crisps and savoury snacks	2.47	92
43R	Sugar confectionery	0.32	1
44R	Chocolate confectionery	1.07	78
45R	Fruit juice	1.01	157
47A	Liqueurs	1.00	1
47B	Spirits	1.00	1
48A	Wine	1.00	1
48B	Fortified wine	1.00	1
48C	Low alcohol and alcohol free wine	1.00	1
49A	Beers and lagers	3.80	14
49B	Low alcohol and alcohol free beer and lager	3.80	14
49C	Cider and perry	0.08	1
49D	Low alcohol and alcohol free cider and perry	3.80	1
49E	Alcoholic soft drinks	0.80	28
50A	Beverages dry weight	1.80	119
50C	Soup manufactured / retail	1.25	27
50D	Soup homemade	0.47	6
50E	Nutrition powders and drinks	0.00	0
50R	Savoury sauces pickles gravies and condiments	1.54	27
51A	Coffee (made up weight)	0.79	1955
51B	Tea (made up weight)	0.33	221
51C	Herbal tea (made up weight)	0.40	1
51D	Bottled water still or carbonated	0.40	1
51R	Tap water only	1.00	1
52A	Commercial toddlers drinks	0.00	0
52R	Commercial toddlers foods	0.00	0
53A	Non dairy ice cream	2.05	94
53R	Ice cream	3.82	44
55R	Artificial sweeteners	3.20	487
56R	Nuts and seeds	1.57	1415
57A	Soft drinks not low calorie concentrated	0.40	1
57B	Soft drinks not low calorie carbonated	0.40	1
57C	Soft drinks not low calorie rtd still	0.40	1
58A	Soft drinks low calorie concentrated	0.40	1
58B	Soft drinks low calorie carbonated	0.40	1
58C	Soft drinks low calorie rtd still	0.40	1
59R	Brown granary and wheatgerm bread	0.97	0
60R	1% fat milk	1.53	23
61R	Smoothies 100% fruit and / or juice	1.05	54

For peer review only

Appendix 6 - References for GHG and Blue WF figures

Amienyo D, Gujba H, Stichnothe H and Azapagic A (2013). Life cycle environmental impacts of carbonated soft drinks. *International Journal of Life Cycle Assessment* 18(1): 77-92.

Azapagic A, Bore J, Cheserek B, Kamunya S and Elbehri A (2016). The global warming potential of production and consumption of Kenyan tea. *Journal of Cleaner Production* 112(5): 4031-4040.

Bartzas G, Vamvuka D and Kommitsas K (2017). Comparative life cycle assessment of pistachio, almond and apple production. *Information Processing in Agriculture* 4(3): 188-198.

Bronmer E, Stratmann B and Quack D (2011). Environmental impacts of different methods of coffee preparation. *International Journal of Consumer Studies* 35(2): 212-220.

Buschspies B, Tolle SJ and Jungbluth N (2011). *Life Cycle Assessment of High-Sea Fish and Salmon Aquaculture.* ESU Services, Uster.

Carbon Trust (2017). *Report to Marlow Foods on Product Carbon Footprint Certification.*

Cichorowski G, Joa B, Hottenroth H and Schmidt M (2015). Scenario analysis of life cycle greenhouse gas emissions of Darjeeling tea. *International Journal of Life Cycle Assessment* 20(4): 426-439.

Clune S, Crossin E and Verghese K (2017). Systematic review of greenhouse gas emissions for different fresh food categories. *Journal of Cleaner Production* 140(2): 766-783.

Djekic I, Miocinovic J, Tomasevic I, Smigic N and Tomic N (2014). Environmental life-cycle assessment of various dairy products. *Journal of Cleaner Production* 68: 64-72.

Doublet G and Jungbluth N (2010). *Life Cycle Assessment of Drinking Darjeeling Tea.* ESU-Services, Uster.

Espinoza-Orias N, Stichnothe H and Azapagic A (2011). The carbon footprint of bread. *International Journal of Life Cycle Assessment* 16(4): 351-365.

Fantin V, Buttol P, Pergreffi R and Masoni P (2012). Life cycle assessment of Italian high quality milk production. A comparison with an EPD study. *Journal of Cleaner Production* 28: 150-159.

Finnegan W, Goggins J, Clifford E and Zhan X (2017). Global warming potential associated with dairy products in the Republic of Ireland. *Journal of Cleaner Production* 163: 262-273.

Frankowska A, Jeswani HK and Azapagic A (2019). Environmental impacts of vegetables consumption in the UK. *Science of the Total Environment* 682: 80-105.

Gonzalez-Garcia S, Gomez-Fernandez Z, Dias AC, Feijoo G, Moreira MT and Arroja L (2014). Life Cycle Assessment of broiler chicken production: a Portuguese case study. *Journal of Cleaner Production* 74: 125-134.

Huerta AR, Guereca LP and Lozano MSR (2016). Environmental impact of beef production in Mexico through life cycle assessment. *Resources, Conservation and Recycling* 109: 44-53.

Humbert S, Loerencik Y, Rossi V, Margni M and Jolliet O (2009). Life cycle assessment of spray dried soluble coffee and comparison with alternatives (drip filter and capsule espresso). *Journal of Cleaner Production* 17(15): 1351-1358.

For peer review only

Iriarte A, Almeida MG and Villalobos P (2014). Carbon footprint of premium quality export bananas: case study in Ecuador, the world's largest exporter. *Science of the Total Environment* 472: 1082-1088.

Jeswani HK, Burkinshaw R and Azapagic A (2015). Environmental sustainability issues in the food-energy-water nexus: breakfast cereals and snacks. *Sustainable Production and Consumption* 2: 17-28.

Jungbluth N (2006). *Comparison of the Environmental Impact of Tap Water vs. Bottled Mineral Water.* Swiss Gas and Water Association, Uster.

Kendall A, Marvinney E, Brodt S and Zhu W (2015). Life cycle-based assessment of energy use and greenhouse gas emissions in almond production, part I: analytical framework and baseline results. *Journal of Industrial Ecology* 19(6): 1008-1018.

Konstantas A, Stamford L and Azapagic A (2019). Economic sustainability of food supply chains: life cycle costs and value added in the confectionery and frozen desserts sectors. *Science of the Total Environment* 670: 902-914.

Konstantas A, Jeswani HK, Stamford L and Azapagic A (2018). Environmental impacts of chocolate production and consumption in the UK. *Food Research International* 106: 1012-1025.

Kristensen T, Soegaard K, Eriksen J and Mogensen L (2015). Carbon footprint of cheese produced on milk from Holstein and Jersey cows fed hay differing in herb content. *Journal of Cleaner Production* 101: 229-237.

Leinonen I, Williams AG, Wiseman J, Guy J and Kyriazakis I (2012). Predicting the environmental impacts of chicken systems in the United Kingdom through a life cycle assessment: Broiler production systems. *Poultry Science* 91(1): 8-25.

McCarty JA, Sandefur HN, Matlock M, Thoma G and Kim D (2014). Life cycle assessment of greenhouse gas emissions associated with production and consumption of peanut butter in the US. *Transactions of the ASABE* 57(6): 1741-1750.

Mekonnen MM and Hoekstra AY (2011). The green, blue and grey water footprint of crops and derived crop products. *Hydrology and Earth System Sciences* 15(5): 1577-1600.

Mekonnen MM and Hoekstra AY (2012). A global assessment of the water footprint of farm animal products. *Ecosystems* 15(3): 401-415.

Nguyen TLT, Hermansen JE and Mogensen E (2010). Environmental consequences of different beef production systems in the EU. *Journal of Cleaner Production* 18(8): 756-766.

Nilsson K, Flysjo A, Davis J, Sim S, Unger N and Bell S (2010). Comparative life cycle assessment of margarine and butter consumed in the UK, Germany and France. *International Journal of Life Cycle Assessment* 15(9): 916-926.

Nilsson K, Sund V and Floren B (2011). *The Environmental Impact of the Consumption of Sweets, Crisps and Soft Drinks.* Nordic Council of Ministers, Copenhagen.

Noya I, Aldea X, Gasol CM, Gonzalez-Garcia S, Amores MJ, Colon J, Ponsa S, Roman I, Rubio MA, Casas E, Moreira MT and Boschmonart-Rives J (2016). Carbon and water footprint of pork supply chain in Catalonia: From feed to final products. *Journal of Environmental Management* 171: 133-143.

For peer review only

Opio C, Gerber P, Mottet A, Falcucci A, Tempio G, MacLeod M, Vellinga T, Henderson B and Steinfeld H (2013). *Greenhouse gas emissions from ruminant supply chains – A global life cycle assessment*. Food and Agriculture Organization of the United Nations (FAO), Rome.

Pahlow M, van Oel PR, Mekonnen MM and Hoekstra AY (2015). Increasing pressure on freshwater resources due to terrestrial feed ingredients for aquaculture production. *Science of the Total Environment* 536: 847-857.

Poore J and Nemecek T (2018). Reducing food's environmental impacts through producers and consumers. *Science* 360: 987-992.

Prudencio da Silva V, van der Werf HMG, Soares SR and Corson MS (2014). Environmental impacts of French and Brazilian broiler chicken scenarios: An LCA approach. *Journal of Environmental Management* 133: 222-231.

Recanti F, Marveggio D and Dotelli G (2018). From beans to bar: a life cycle assessment towards sustainable chocolate supply chain. *Science of the Total Environment* 613-614: 1013-1023.

Recchia L, Cappelli A, Cini E, Pegna FG and Boncinelli P (2019). Environmental sustainability of pasta production chains: an integrated approach for comparing local and global chains. *Resources* 8(1): 56.

Scarborough P, Appleby PN, Mizdrak A, Briggs ADM, Travis RC, Bradbury KE and Key TJ (2014). Dietary greenhouse gas emissions of meat-eaters, fish-eaters, vegetarians and vegans in the UK. *Climatic Change* 125(2): 179-192.

Seabra JEA, Macedo IC, Chum HL, Faroni CE and Sarto CA (2011). Life cycle assessment of Brazilian sugarcane products: GHG emissions and energy use. *Biofuels, Bioproducts and Biorefining* 5(5): 519-532.

Sheane R, Lewis K, Hall P, Holmes-Ling P, Kerr A, Stewart K and Webb D (2011). *Identifying Opportunities to Reduce the Carbon Footprint Associated with the Scottish Dairy Chain – Main Report.* Edinburgh: Scottish Government.

Svanes E and Aronsson AKS (2013). Carbon footprint of a Cavendish banana supply chain. *International Journal of Life Cycle Assessment* 18(8): 1450-1464.

Wiedemann S, McGahan E, Murphy C, Yan M-J, Henry B, Thoma G and Ledgard S (2015). Environmental impacts and resource use of Australian beef and lamb exported to the USA determined using life cycle assessment. *Journal of Cleaner Production* 94: 67-75.

Ziegler F, Winther U, Skontorp Hognes E, Emmanuelsson A, Sund V, Ellingsen H (2012). The carbon footprint of Norwegian seafood products on the global seafood market. *Journal of Industrial Ecology* 17(1): 103-116

For peer review only

Appendix 7A – Calculation of weighted GHG and water footprint of the food group aggregate "fruit and vegetables" – based on proportional supply by crop and country of origin (FAOStat 2013 data)

Crops	% of total supply	GHG per kg	Blue WF per kg
Tomatoes	13.4	0.96	36
Bananas	7.8	1.07	49
Grapes**	6.3	1.34	298
Apples	6.1	0.75	77
Onions, dry	6.0	0.76	36
Carrots and turnips	5.8	1.28	0
Oranges	5.0	0.4	93
Cauliflowers and broccoli	2.5	1.03	53
Cabbages and other brassicas	2.5	0.67	7
Lettuce and chicory	2.3	1.59	41
Tangerines, mandarins, clementines, satsumas	2.0	0.4	93
Mushrooms and truffles	1.6	0.76	2
Chillies and peppers, green	1.6	0.76	2
Cucumbers and gherkins	1.5	0.2	12
Olives**	1.4	1.34	298
Pineapples**	1.4	1.34	298
Maize, green	1.3	0.76	2
Peas, green	1.3	2.55	23
Pears**	1.3	0.75	77
Melons, other (inc.cantaloupes)**	1.1	1.34	298
Strawberries	1.0	1.7	28
Lemons and limes	0.84	1.01	157
Plums and sloes	0.82	1.7	28
Pumpkins, squash and gourds	0.75	0.5	55
Peaches and nectarines**	0.64	1.34	298
Grapefruit (inc. pomelos)	0.60	0.4	93
Watermelons**	0.53	1.34	298
Apricots**	0.46	1.34	298
Sweet potatoes	0.46	0.5	55
Leeks, other alliaceous vegetables	0.42	0.71	15
Mangoes, mangosteens, guavas**	0.41	1.34	298
Beans, green	0.36	0.5	6
Avocados**	0.25	1.34	298
Onions, shallots, green*	0.24	0.76	36

* all other crops contribute less than 0.2% to total UK supply and were disregarded for calculations of weighted GHGe and WF of the fruit and vegetables aggregate.

** Classified as "other fruit" or "other vegetables" - without specific WFs

For peer review only

Appendix 7B - Proportions of main UK imported foods from different countries

For each food group, countries of origin were selected based on a database adapted from FAO Food Balance Sheet data (https://iopscience.iop.org/article/10.1088/1748-9326/9/3/034015/meta). All countries providing at least 10% of the total availability for that food group were included in environmental footprinting, and for the remainder of supply global average figures were applied. Where country-specific footprint data were not available, footprints from the most similar country or a global average were applied.

Food	Country	Proportion of UK consumption
ALMONDS	USA	0.72
	Australia	0.1
	Spain	0.14
	Global	0.04
APPLE JUICE	France	0.14
	South Africa	0.1
	UK	0.36
	Global	0.4
APPLES	France	0.14
	South Africa	0.1
	UK	0.36
	Global	0.4
ASPARAGUS	Mexico	0.11
	Peru	0.46
	Spain	0.12
	UK	0.26
	Global	0.05
AVOCADO	Chile	0.22
	Israel	0.16
	Peru	0.21
	South Africa	0.27
	Global	0.14
BANANAS	Colombia	0.23
	Costa Rica	0.18
	Dominican Republic	0.18
	Ecuador	0.14
	Global	0.27
BARLEY	UK	0.81
	Global	0.19
BEEF	UK	0.76
	Ireland	0.17
	Global	0.07
BLUEBERRIES	Netherlands	0.34
	Poland	0.3

For peer review only

	Global	0.45
CAULIFLOWER/BROCCOLI	UK	0.47
	Spain	0.43
	Global	0.1
CHICKPEAS	Argentina	0.11
	Australia	0.21
	Canada	0.14
	Mexico	0.12
	Global	0.42
COFFEE	Brazil	0.26
	Colombia	0.1
	Indonesia	0.13
	Vietnam	0.25
	Global	0.26
COCOA BUTTER	Ghana	0.27
	Cote d'Ivoire	0.44
	Nigeria	0.1
	Global	0.19
COCOA PASTE	Ghana	0.27
	Cote d'Ivoire	0.44
	Nigeria	0.1
	Global	0.19
COCONUTS	Indonesia	0.24
	Malaysia	0.11
	Philippines	0.48
	Global	0.17
CUCUMBERS	Netherlands	0.34
	Spain	0.33
	UK	0.3
	Global	0.03
GARLIC	China	0.41
	Spain	0.52
	Global	0.07
GRAPES	Chile	0.1
	France	0.1
	Italy	0.15
	Spain	0.1
	Turkey	0.18
	Global	0.37
GREEN BEANS	Egypt	0.15
	Kenya	0.32
	UK	0.32
	Global	0.21
GROUNDNUTS	USA	0.26

For peer review only

	Nicaragua	0.13
	China	0.14
	Argentina	0.33
	Global	0.14
HAZELNUTS	Georgia	0.18
	Italy	0.1
	Turkey	0.69
	Global	0.03
LEMON JUICE	Argentina	0.16
	Brazil	0.13
	Spain	0.47
	Global	0.24
LEMONS	Argentina	0.16
	Brazil	0.13
	Spain	0.47
	Global	0.24
LENTILS	Canada	0.63
	Turkey	0.26
	Global	0.11
LETTUCE	Spain	0.49
	UK	0.4
	Global	0.11
MAIZE	France	0.27
	Ukraine	0.18
	Global	0.55
MILLET	Russia	0.32
	France	0.24
	Ukraine	0.13
	India	0.1
	Global	0.21
NUTS, OTHER	China	0.13
	Turkey	0.3
	USA	0.12
	Global	0.45
OLIVE OIL	Italy	0.18
	Spain	0.59
	Global	0.23
ONIONS	Netherlands	0.23
	Spain	0.16
	UK	0.44
	Global	0.17
ORANGE JUICE	Brazil	0.3
	South Africa	0.13
	Spain	0.28

For peer review only

	USA	0.1
	Global	0.19
ORANGES	Brazil	0.3
	South Africa	0.13
	Spain	0.28
	USA	0.1
	Global	0.19
PALM OIL	Indonesia	0.43
	Malaysia	0.28
	Papua New Guinea	0.2
	Global	0.09
PEACHES	Italy	0.14
	Spain	0.73
	Global	0.13
PEARS	Netherlands	0.32
	South Africa	0.17
	UK	0.13
	Global	0.38
PEPPERS AND CHILLIES	Netherlands	0.38
	Spain	0.34
	UK	0.11
	Global	0.17
PINEAPPLES	Costa Rica	0.7
	Thailand	0.14
	Global	0.16
POTATOES	UK	0.72
	Netherlands	0.11
	Global	0.17
PRUNES (PLUMS)	Chile	0.24
	Spain	0.16
	UK	0.11
	USA	0.14
	Global	0.35
RAISINS	Turkey	0.3
	USA	0.3
	South Africa	0.1
	Chile	0.1
	Global	0.2
RICE (WHITE)	India	0.26
	Spain	0.18
	Italy	0.13
	Pakistan	0.11
	Global	0.32
RICE (BROWN)	India	0.26

For peer review only

	Spain	0.18
	Italy	0.13
	Pakistan	0.11
	Global	0.32
SOYA PROTEIN	Brazil	0.44
	Argentina	0.31
	USA	0.16
	Global	0.09
SOYBEANS	Argentina	0.31
	Brazil	0.44
	USA	0.16
	Global	0.09
SOYBEANS FOR MILK	Brazil	0.44
	Argentina	0.31
	USA	0.16
	Global	0.09
SPINACH	Italy	0.11
	Spain	0.7
	Global	0.19
SUGAR	UK	0.38
	Global	0.62
SUNFLOWER OIL	Argentina	0.12
	France	0.14
	Ukraine	0.31
	Global	0.43
SUNFLOWER SEEDS	Argentina	0.12
	France	0.14
	Ukraine	0.31
	Global	0.43
TEA	India	0.16
	Indonesia	0.15
	Kenya	0.39
	Global	0.3
TOMATO PASTE	Italy	0.39
	Spain	0.2
	Portugal	0.13
	Global	0.28
TOMATOES	Italy	0.39
	Spain	0.2
	Portugal	0.13
	Global	0.28
WHEAT FLOUR	UK	0.67
	Global	0.33

For peer review only

For peer review only

Appendix 8 – Differences in EWG dietary guidelines adherence

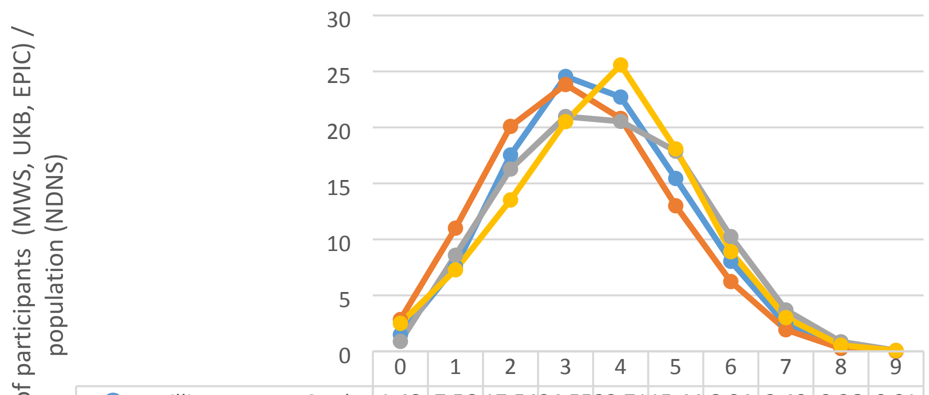

	0	1	2	3	4	5	6	7	8	9
Million Women Study	1.48	7.56	17.54	24.55	22.71	15.44	8.04	2.40	0.26	0.01
UK Biobank	2.84	11.00	20.09	23.83	20.80	13.00	6.23	1.93	0.27	NA
EPIC Oxford	0.89	8.58	16.28	20.97	20.54	17.88	10.25	3.69	0.85	0.08
NDNS	2.50	7.28	13.52	20.52	25.58	18.08	8.90	3.01	0.54	0.08

peer review only

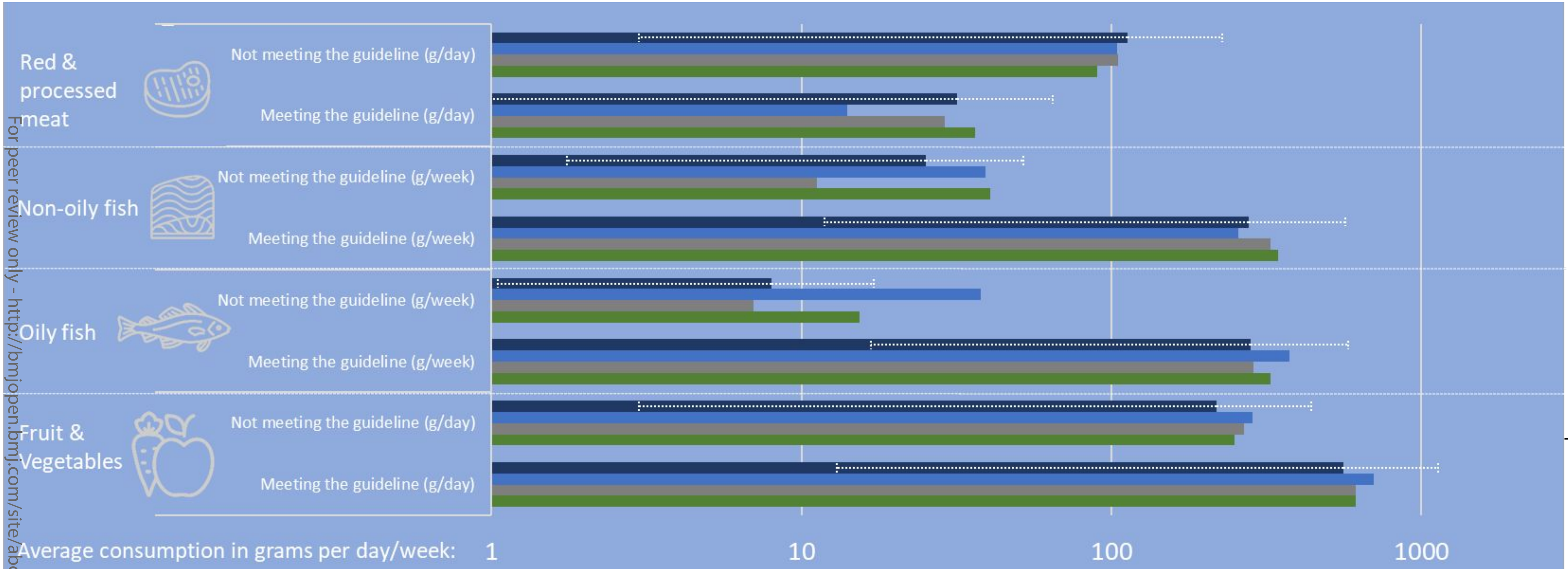

	Fruit and vegetables		Oily fish		Non-oily fish		Red & processed meat	
	Meeting the guideline (g/day)	Not meeting the guideline (g/day)	Meeting the guideline (g/week)	Not meeting the guideline (g/week)	Meeting the guideline (g/week)	Not meeting the guideline (g/week)	Meeting the guideline (g/day)	Not meeting the guideline (g/day)
NDNS	561.0	218.0	282.1	8.0	277.9	25.3	31.8	113.0
EPIC	704.8	286.4	375.2	37.8	257.6	39.2	14.0	104.5
UK Bio	614.4	267.2	287.0	7.0	326.2	11.2	29.0	105.1
MWS	614.4	249.6	327.6	15.4	345.8	40.6	36.3	90.0

For peer review only - http://bmjopen.bmj.com/site/about/guidelines.xhtml

Appendix 9 – Greenhouse Gas Emissions and Water Footprints by adherence group adjusted for energy intake (unweighted regression models)

(Unweighted) regression analysis of greenhouse gas emission by adherence group – 1) crude and 2) adjusted for energy intake.

Greenhouse Gas Emissions (in kg CO_2eq per day) adjusted for total caloric intake				
**Crude model**	Mean	SE	Lower CI	Upper CI
**0-2 guidelines**	4.69	0.054	4.80	4.58
**3-4 guidelines**	3.76	0.039	3.84	3.68
**5-9 guidelines**	3.53	0.053	3.63	3.43
**Adjusted model**	Mean	SE	Lower CI	Upper CI
**0-2 guidelines**	4.69	0.050	4.79	4.59
**3-4 guidelines**	3.76	0.040	3.84	3.68
**5-9 guidelines**	3.53	0.048	3.62	3.44
**Blue Water Footprints (in litres per day) adjusted for total caloric intake**				
**Crude model**	Mean	SE	Lower CI	Upper CI
**0-2 guidelines**	462	15.2	492	432
**3-4 guidelines**	458	11.0	480	436
**5-9 guidelines**	525	15.0	554	496
**Adjusted model**	Mean	SE	Lower CI	Upper CI
**0-2 guidelines**	462	16.8	495	429
**3-4 guidelines**	458	12.6	483	433
**5-9 guidelines**	525	16.3	557	493

For peer review only

BMJ Open

HEALTH IMPACTS AND ENVIRONMENTAL FOOTPRINTS OF DIETS THAT MEET THE EATWELL GUIDE RECOMMENDATIONS: ANALYSES OF MULTIPLE UK STUDIES

Journal:	*BMJ Open*
Manuscript ID	bmjopen-2020-037554.R1
Article Type:	Original research
Date Submitted by the Author:	15-Jun-2020

Complete List of Authors:	Scheelbeek, Pauline; London School of Hygiene and Tropical Medicine Faculty of Epidemiology and Population Health, Population Health<br>Green, Rosemary; London School of Hygiene and Tropical Medicine, Population health<br>Papier, Keren; University of Oxford Nuffield Department of Population Health, Cancer Epidemiology Unit<br>Knuppel, Anika ; University of Oxford Nuffield Department of Population Health, Cancer Epidemiology Unit<br>Alae-Carew, Carmelia; London School of Hygiene and Tropical Medicine, Epidemiology & Biostatistics<br>Balkwill, Angela; Oxford University<br>Key, Timothy; University of Oxford, Cancer Epidemiology Unit, Nuffield Department of Population Health<br>Beral, Valerie; Oxford University, Nuffield Department of Population Health<br>Dangour, Alan; London School of Hygiene & Tropical Medicine, Nutrition and Public Health Intervention Research

**Primary Subject Heading**:	Global health
Secondary Subject Heading:	Epidemiology, Evidence based practice, Health policy, Nutrition and metabolism
Keywords:	EPIDEMIOLOGY, NUTRITION & DIETETICS, PREVENTIVE MEDICINE, PUBLIC HEALTH

SCHOLARONE™
Manuscripts

I, the Submitting Author has the right to grant and does grant on behalf of all authors of the Work (as defined in the below author licence), an exclusive licence and/or a non-exclusive licence for contributions from authors who are: i) UK Crown employees; ii) where BMJ has agreed a CC-BY licence shall apply, and/or iii) in accordance with the terms applicable for US Federal Government officers or employees acting as part of their official duties; on a worldwide, perpetual, irrevocable, royalty-free basis to BMJ Publishing Group Ltd ("BMJ") its licensees and where the relevant Journal is co-owned by BMJ to the co-owners of the Journal, to publish the Work in this journal and any other BMJ products and to exploit all rights, as set out in our licence.

The Submitting Author accepts and understands that any supply made under these terms is made by BMJ to the Submitting Author unless you are acting as an employee on behalf of your employer or a postgraduate student of an affiliated institution which is paying any applicable article publishing charge ("APC") for Open Access articles. Where the Submitting Author wishes to make the Work available on an Open Access basis (and intends to pay the relevant APC), the terms of reuse of such Open Access shall be governed by a Creative Commons licence – details of these licences and which Creative Commons licence will apply to this Work are set out in our licence referred to above.

Other than as permitted in any relevant BMJ Author's Self Archiving Policies, I confirm this Work has not been accepted for publication elsewhere, is not being considered for publication elsewhere and does not duplicate material already published. I confirm all authors consent to publication of this Work and authorise the granting of this licence.

For peer review only

HEALTH IMPACTS AND ENVIRONMENTAL FOOTPRINTS OF DIETS THAT MEET THE EATWELL GUIDE RECOMMENDATIONS: ANALYSES OF MULTIPLE UK STUDIES

*Pauline F.D. Scheelbeek – Centre on Climate Change and Planetary Health, London School of Hygiene & Tropical Medicine, London UK
Rosemary Green – Centre on Climate Change and Planetary Health, London School of Hygiene & Tropical Medicine, London UK
Keren Papier –Nuffield Department of Population Health, University of Oxford, Oxford, UK
Anika Knuppel – Nuffield Department of Population Health, University of Oxford, Oxford, UK
Carmelia Alae-Carew – Department of Population Health, London School of Hygiene & Tropical Medicine, London UK
Angela Balkwill - Nuffield Department of Population Health, University of Oxford, Oxford, UK
Timothy Key - Nuffield Department of Population Health, University of Oxford, Oxford, UK
Valerie Beral - Nuffield Department of Population Health, University of Oxford, Oxford, UK
Alan D. Dangour – Centre on Climate Change and Planetary Health, London School of Hygiene & Tropical Medicine, London UK

*Corresponding author – Keppel Street, London WC1E 7HD, United Kingdom - pauline.scheelbeek@lshtm.ac.uk - +44.207.612.7914

Keywords: sustainable food systems; Eatwell Guide; nutritional health; dietary footprint; environmental footprint

Word count (excluding title page, abstract, references, figures): 3400

For peer review only

Abstract

Objectives – To assess the health impacts and environmental consequences of adherence to national dietary recommendations (the Eatwell Guide) in the UK.

Design and Setting – A secondary analysis of multiple observational studies in the UK

Participants – Adults from the EPIC-Oxford, UK Biobank and Million Women Study, and adults and children aged 5 and over from the National Diet and Nutrition Survey (NDNS)

Primary and Secondary Outcome Measures – risk of total mortality from Cox proportional hazards regression models, total greenhouse gas (GHG) emissions and blue water footprint associated with "very low" (0-2 recommendations), "low" (3-4 recommendations) or "intermediate to high" (5-9 recommendations) adherence to Eatwell Guide recommendations

Results – Less than 0.1% of the NDNS sample adhere to all nine EWG recommendations and 30.6% adhere to at least five recommendations. Compared to "very low" adherence to EWG recommendations, "intermediate to high adherence" was associated with a reduced risk of mortality (Risk ratio (RR): 0.93; 99% confidence interval (CI): 0.90 to 0.97) and -1.6kg CO_2eq/day (95% CI -1.5 to -1.8), or 30% lower dietary GHG emissions. Dietary water footprints were similar across EWG adherence groups. Of the individual Eatwell guidelines, adherence to the recommendation on fruit and vegetable consumption was associated with the largest reduction in total mortality risk: a RR of 0.90 (99% CI 0.88, 0.93). Increased adherence to the recommendation on red and processed meat consumption was associated with the largest decrease in environmental footprints (-1.48kg CO_2eq/day, 95% CI -1.79, -1.18 for GHG emissions and -22.5 litres/day, 95% CI -22.7, -22.3 for blue water footprint).

Conclusions – The health and environmental benefits of greater adherence to EWG recommendations support increased government efforts to encourage improved diets in the UK that are essential for the health of people and the planet in the Anthropocene.

Strengths and limitations of this study

- This is the first study (in a UK context) using empirical data to study health impacts and multiple environmental consequences of sustainable diets
- The study uses multiple high-quality datasets with a total of 557,722 participants for health outcomes and 5,747 participants for environmental footprints
- The provided methods can be replicated in other settings and the Eatwell Guide dietary recommendations share many features of healthy lower environmental impact diets
- Despite several sensitivity analyses, there might be residual confounding (i.e. unmeasured differences between people who eat different diets) distorting our findings
- Although the evidence base and quality of methods and metrics for environmental footprints are rapidly improving, uncertainty about the exact measurements of water footprints and greenhouse gas emissions of food items and diets in general remain

For peer review only

Background

Diets are likely to play a crucial role in the Anthropocene in supporting population health and safeguarding environmental sustainability for future generations. Current diets are associated with a high burden of disease: globally ~1.9 billion adults are overweight or obese, 462 million are underweight [1], and over 30% of the world's population suffers from deficiencies of essential nutrients [2]. The food system that produces these diets is also responsible for 21-37% of global greenhouse emissions (GHGe) [3] and agriculture alone accounts for ~70% of fresh water withdrawal [4]. Whilst food system GHGe contribute to global climate change problems regardless of location of production, food system water use is highly location specific: with approximately half of all countries classified as "water scarce" - and a number of water safe countries projected to become water scarce by 2040 [5] - origin of food supply is a crucial consideration when considering the sustainability of food system water use.

There is an urgent need for significant transformations of the food system to produce diets that address both health and environmental concerns, and evidence on the recommended composition of these diets is expanding rapidly [6, 7]. While the specific composition of such diets has been shown to vary considerably culturally and regionally compared to existing consumption patterns, these diets typically have substantially greater plant-based food content as well as no more than moderate content of animal sourced foods (e.g. meat and dairy) (e.g. [6, 8, 9]).

The UK food system is no exception to these concerns for sustainability, and many transformative changes need to be made to make it more sustainable, resilient and healthy. Currently 64% of the adult population in the UK are overweight or obese [10], and only 29% of adults and 18% of children between 5 and 15 years of age meet the recommended fruit and vegetable intake of "5-a-day" [10] . At the same time, water use of UK diets is on average 2,757 litre per capita per day, which is below the global average of 3,167 litre [11], but half of the national blue (surface and ground) water footprint - 15.0 million m^3 per day - is imported (i.e. embedded in imported foods from elsewhere) from countries with water scarcity [12, 13]. Furthermore, GHGe of average UK diets were found to be 1210 kg CO_2eq per capita per year as compared to a EU average of 1070 kg CO_2eq per capita per year [14]. Evidence suggests that 17% of emissions could be avoided when the population were to shift to WHO dietary guidelines[15].

Governments are increasingly including both health and environmental considerations in their recommended dietary guidelines. In the UK, Public Health England produced the "Eatwell Guide" (EWG) as a "policy tool to define government recommendations on eating healthily and achieving a balanced diet" [16]. From a health perspective, the EWG promotes, for example, cereals, potatoes, fruit, vegetable and fibre consumption, whilst recommending a limited consumption of sugar and processed meats [17]. Adhering to these individual guidelines has been associated with several health benefits including improved cardiovascular health [18] and reduced cancer risk [19, 20]. From an environmental perspective the EWG mentions the importance of a "balance of healthier and more sustainable food", while providing information about protein alternatives, such as beans, peas and lentils that typically have a lower environmental footprint than animal source food protein sources [20-23]. Compared to current diets, the EWG recommendations are, therefore, expected to have on average lower environmental footprints (GHG emissions, water and land use requirements) [24]. The guidelines on meat and dairy, that are both set substantially below current average intake, were projected to have the largest impact on reduction of GHG emission, land use and eutrophication [25]. GHGe of meat eaters in the UK was found to be roughly double that of vegans [23].

For peer review only

Whilst modelling studies have estimated the change in GHGe when shifting from current EWG adherent diets, these are subject to many assumptions related to the substitutions between food groups inherent to the dietary change. To date, no study has been conducted using empirical dietary data (of large-scale cohort studies) to assess "real world" composition of diets that are in adherence with the EWG, which could substantially improve the uncertainty of estimation of the associated change in dietary GHGe. Furthermore, to date no analysis of the water footprint of EWG adherence has been published.

In this report we use high-quality data from three large prospective UK cohort studies to assess the health impacts associated with adherence to EWG dietary guidelines and using nationally-representative dietary intake data we estimate the environmental footprints of UK diets with varying degrees of adherence to EGW recommendations.

Methods

Datasets

We used four high-quality data sources in this paper (detailed description of each database provided in Appendix 1). The databases from EPIC-Oxford (EPIC-Ox) [26], UK Biobank (UKB) [27] and the Million Women Study (MWS) [28] contain comprehensive health information, linked death registration data, as well as dietary intake data. These three datasets were used to estimate the associations with health of adherence to EWG recommendations. Details on the specific datasets have been published elsewhere. Briefly, participants in the MWS were recruited from women invited for breast cancer screening in England and Scotland between 1996 and 2001. Dietary intake was collected using semi-quantitative questions and total mortality was determined using death records. We used data from 464,078 participants of the MWS database. In the EPIC-Ox study, which involves both male and female participants, dietary intake was collected using a food-frequency questionnaire, whilst total mortality was ascertained using death record linkage. We used data from 40,030 men and women of the EPIC-Ox database. For the UKB study, middle-aged adults were recruited between 2006-2010. A sub-sample completed a minimum of three 24-hour dietary recall questionnaires. Participant data have been linked to the NHS Central register to obtain mortality information. We used data from 53,614 participants of the UKB study. Finally, the National Diet and Nutrition Survey (NDNS) [29] contains nationally representative detailed dietary intake data that were used to analyse the diet-related environmental footprint of NDNS participants with different levels of EWG adherence. We excluded children <5 years of age from the NDNS data, as the EWG recommendations are not applicable to this age group.

Eatwell Guide dietary recommendations

Dietary intakes reported in each of the four databases were compared to recommended intakes by the EWG and dichotomized (yes/no) to reflect individual adherence to EWG recommendations (recommendations by age and sex provided in Appendix 2). Nine food and nutrient groups with recommended levels of consumption specified in the EWG were considered: fruit and vegetables; oily fish; other fish; red and processed meat; total fibre; total salt; free sugars; saturated fatty acids; and total fat. Two further EWG recommendations on protein and carbohydrates were excluded as significant heterogeneity across foodstuffs included in the questionnaires limit conversion from % of food energy intake to grams per day (see also [30]). Participants were grouped into three categories of adherence based on the

For peer review only - http://bmjopen.bmj.com/site/about/guidelines.xhtml

number of dietary recommendations met (total = 9): very low adherence [score 0-2]; low adherence [score 3-4]; and intermediate to high adherence [score 5-9].

Health impacts

We used multivariable-adjusted Cox proportional hazards regression models to assess associations between adherence to the EWG dietary guidelines and risk of total mortality, ascertained through death registries using participant data of EPIC-Oxford (EPIC-Ox), the Million Women Study (MWS), and a subset of UK Biobank with detailed dietary data (UKB). These estimates were combined using meta-analytical methods to provide pooled risk ratios (RR). The mean follow-up time was 21.0 years in EPIC Ox, 10.5 years in MWS and 3.9 years in UKB.

Participants in each database were excluded from the analysis sample if: (1) they had prevalent and/or unknown status of malignant cancer, diabetes or cardiovascular disease (data based on self-report and health record data) or rated their overall health as either poor or fair at recruitment; (2) they had energy intakes outside the ranges 2,093-14,654 kJ for women and 3,349-16,747 kJ for men, and did not report: a change in diet because of illness (MWS), not eating or drinking normally because of illness or fasting (UKB), because of stomach problems, bowel problems or diabetes (EPIC-Ox) and in UKB had not completed a minimum of three WebQ questionnaires; (3) they were lost to follow-up during the first 5 years of follow-up (MWS and EPIC-Ox only), and; (4) their smoking status was unknown.

Associations were stratified by sex, region and method of recruitment (in addition to the general recruitment strategy, specific underrepresented groups were targeted for recruitment by leaflets – which could have introduced selection bias), where appropriate. All analyses were adjusted for smoking, deprivation, alcohol consumption, height, body mass index (BMI), exercise levels, hormone replacement therapy use, education, high blood pressure or hypertension and energy intake (Appendix 3 for details). We performed a set of seven sensitivity analyses, comparing the above model with a) an unadjusted model, models without adjustment for b) energy, c) height, d) BMI or e) smoking, f) a model mutually adjusting for all other eight food groups, and g) a model excluding smokers (Appendix 4).

For peer review only

Environmental footprints

We used data from NDNS waves 5-9 (2012-2017) to map the environmental footprints of diets in the UK. The database comprises detailed dietary data for 5,747 individuals aged five years and over, grouped into 158 distinct food group aggregates. Data collection methods are described in detail elsewhere [31]. We used the Food and Agriculture Organization (FAO) bilateral trade database to estimate the mean proportion of each food group imported from outside the UK [32]. The trade database includes bilateral data on exports and imports of all food and agricultural products reported by all the countries in the world.

Greenhouse Gas Emissions - Emissions of GHGs across the life cycle (kg CO2e/kg food) for the 158 distinct food group aggregates were derived from published data (Appendix 5&6). A weighted average of GHG emissions was calculated based on consumption of individual foods within each food group and proportion of supply from different countries. For foods entirely or more than 90% produced in the UK, UK-specific data were used. A weighted average for GHG emissions was applied for imported foods based on the proportion of total supply from various countries (Appendix 7).

Water footprints (WFs) - The blue (ground and surface water) WF (L/g food) of crop and livestock products were derived from published data for 1996-2005 from the Water Footprint Network (WFN, [33]) (Appendix 5&6). For foods entirely or more than 90% produced in the UK, UK-specific WFN values were used. Imported food groups were assigned weights proportional to percentage of overall supply of each major exporting country to the UK, multiplied by WFN estimates for that particular country and food group (Appendix 7).

The estimated GHG emissions and WFs associated with each food group were used to quantify total environmental footprints associated with the daily diet of each participant in the NDNS database. We compared GHG emissions and WFs of diets of those adhering and those not adhering to each EWG dietary guidelines, and estimated the mean change in environmental footprint that would occur if individuals shifted from low to intermediate/high adherence to the EWG guidelines.

Results

EWG-adherence

Less than 0.1% of the NDNS sample (0.078%) adhered to all nine EWG recommendations (Figure 1A), with the largest proportion of the population (44%) adhering to 3-4 guidelines. The most commonly unmet recommendations included those on consumption of dietary fibre and oily fish (7.2% and 16.8% adherence respectively), while more than 50% of the population met total and saturated fat, salt and red and processed meat recommendations (Figure 1B). Adherence to the EWG recommendations in EPIC-Ox, MWS and UKB showed a similar pattern to that in the NDNS data set (Appendix 8).

Figure 1: Adherence to the Eatwell Guide recommendations by the UK population - based on data from wave 5-9 of the National Dietary and Nutrition Survey (NDNS): A) total number of recommendations met by % of UK population; B) adherence to specific recommendations

Health effects of adherence to EWG recommendations

Compared with those who had a very low adherence to the EWG, individuals with intermediate to high adherence had a 7% [99% CI: 3 to 10%] reduced risk of total mortality (Figure 2). Sensitivity analysis identified smoking as an important confounder and hence the main analysis was adjusted for smoking. Other potential confounders showed to only marginally affect associations detected in the main model.

Figure 2: Forest plot showing the study specific (MWS, UKB & EPIC-Ox) and pooled mortality risk ratios comparing very poor adherence to EWG rocmmendations (score 0-2) with poor adherence (score 3-4) and intermediate to high adherence (score 5-9).

Adherence to the recommendation on fruit and vegetable consumption was independently associated with the largest reduction in total mortality risk: a reduction of 10% [RR: 0.90; 99% CI: 0.88 – 0.93] (Figure 3; attenuated to 9% in models adjusting for all other EWG recommendations see Appendix 4). Meeting the recommendations on saturated fat and oily fish consumption showed smaller associations with health benefits, with 5% and 3% reductions in mortality respectively (both attenuated to 3% in models adjusting for all other

For peer review only

EWG recommendations see [Appendix 4]). There was no consistent evidence of an association with mortality risk for adherence to other EWG recommendations (Figure 3 & Appendix 4 – with recommendation based on dietary reference values for food energy and nutrients for the United Kingdom [34]).

*Figure 3: Mortality risk ratios for the association between adhering to specific EWG recommendations and total mortality. * Recommendation was based on food energy and was therefore adapted to ≥47% of total energy. *Adapted to <=33% of total energy. †Adapted to <=10% of total energy. ‡Information on salt intake was ascertained from the variable 'Never adding salt to food at the table or cooking' in the MWS and in the EPIC-Oxf study; and from the variable 'Not reporting having added salt to food (excluding during cooking)' in any of the WEBQs included in the UKB. §Fibre intake in the study was determined using the Englyst method and the recommendation was therefore adapted to >=22.6g/d of Englyst fibre.*

Environmental footprints of diets

Individuals with intermediate to high adherence to EWG recommendations showed a reduction in average dietary GHG footprints - compared to those with low and very low EWG-adherence - of 12% and 30% respectively: an average of 3.8kg CO_2eq/day (95% CI: 3.7 to 3.9kg CO_2eq /day) , (4.3kg CO_2eq/day [95% CI: 4.1 to 4.4kg CO_2eq /day] and 5.4 kg CO_2eq/day [95% CI: 5.2 to 5.6kg CO_2eq /day] for intermediate to high (score 5-9), low (score 3-4) and very low (score 0-2) EWG adherence respectively) Dietary blue WFs were similar across adherence groups (Figure 4): 637kg CO2eq /day [95% CI 590 to 683], 590kg CO2eq /day [95% CI 558 to 622] and 612kg CO2eq /day [95% CI 571 to 654] respectively for very low, low and intermediate to high adherence to the EWG recommendations. GHG emissions and WFs changed marginally when adjusting for dietary energy intake (Appendix 9).

Figure 4: Average daily greenhouse gas emissions in kg CO_2eq and average daily dietary water footprints comparing diets with very low (score 0-2), low (score 3-4) and intermediate to high adherence (score 5-9) to the Eatwell Guide dietary guidelines.

Mean difference in consumption (in g per day) of foods between EWG adherent and non-adherent individuals was large (Table 1). Associated differences in dietary GHG emissions were small for fruit and vegetables, oily fish and non-oily fish consumption, and adherence to the recommendation on red and processed meat was associated with lower GHG emissions (-1.48kg CO2eq/day; 95% CI -1.79 to -1.18) (Table 1). Differences in blue water footprints were small for oily fish and non-oily fish consumption, and adherence to the fruit and vegetable recommendation was associated with a larger blue water footprint (+28.5 litres per person per day; 95% CI: 17.4 to 39.8) while adherence to the red and processed meat recommendation was associated with a lower blue water footprint (-22.5 litres per person per day; 95% CI: -22.7 to -22.3).

	**Dietary Recommendation**			
	Fruit & vegetables	Oily fish	Non-oily fish	Red & processed meat

For peer review only - http://bmjopen.bmj.com/site/about/guidelines.xhtml

Metric	Unit	Meeting recommendation	not meeting recommendation	Meeting recommendation	not meeting recommendation	Meeting recommendation	not meeting recommendation	Meeting recommendation	not meeting recommendation
**Weighted average consumption**	***g/day (SE)***	561 (6.47)	218 (2.00)	40.3 (1.23)	1.14 (0.08)	39.7 (0.85)	3.61 (0.13)	31.8 (0.50)	113 (1.30)
**Difference in average consumption**	**g/day**	**+343**		**+39.2**		**+36.1**		**-81.2**	
**Mean difference in GHGe achieved by switching to meeting guideline**	**kg CO_2eq/day (95% CI)**	0.34 (0.29 to 0.38)		0.18 (0.04 to 0.31)		0.34 (0.23 to 0.45)		-1.48 (-1.79 to -1.18)	
**Mean difference in blue WF achieved by switching to meeting guideline**	**l/day (95% CI)**	28.5 (17.4 to 39.8)		10.0 (9.37 to 10.7)		8.23 (7.69 to 8.77)		-22.5 (-22.7 to -22.3)	

Table 1: Mean per-capita change in environmental footprints from switching from non-adherence to adherence to food-based EWG recommendations (*from current level of adherence to adherence by all)*

Discussion

Adherence to the Eatwell Guide is currently low among the UK population. Our analysis of three large UK cohort studies suggests that greater adherence is associated with population health benefits, and using data from the nationally-representative National Diet and Nutrition Survey data, we demonstrate that increased EWG adherence is associated with a lower environmental footprint in terms of GHG emissions, although not water use. Adherence to some EWG recommendations would increase environmental footprints in some instances. Taken together these findings suggest broad benefits to public health and the environment of adherence to the EWG and provide evidence to support strengthened national action to improve diets in the UK for the benefit of people and the planet.

Our findings support earlier analyses [24] showing that UK diets fully compliant with the EWG have lower environmental footprints. Previous studies of the sustainability of UK diets have found that considerable co-benefits to environment and health could be achieved by meeting WHO dietary guidelines [15, 35], increasing adherence to the EAT Lancet diet [36], and following a predominantly plant-based diet [20, 23, 37, 38]. While our analysis confirms that reducing consumption of red and processed meat is paramount for lowering environmental footprints of diets, the analysis suggested that population health benefits would be mainly associated with the recommended consumption of fruit and vegetables.

The estimated 7% reduction in mortality and 30% reduction in emissions (or an average absolute reduction of 0.58 tonne GHGe per person per year) through better adherence to the EWG-guidelines is similar in magnitude as compared to other population-level interventions aiming multiple benefits for health and the environment. For example, a study evaluating a

For peer review only - http://bmjopen.bmj.com/site/about/guidelines.xhtml

future scenario of increased active travel and lower-emission motor vehicles in London estimated a 0.72 tonne reduction in per person GHGe as compared to the business-as-usual scenario, as well as a 10-19% reduction in years of life lost from ischaemic heart disease[39]. A dietary modelling study from the Netherlands estimated impact on GHGe (4-11%) from substituting 35g/d of meat with vegetables, fruit, nuts, seeds, pasta, rice, couscous or fish [40].

A major strength of this study is its use of four large, high-quality data sources for the UK. A number of sensitivity analyses were conducted to test the robustness of the findings to different assumptions about the causal relationships between variables, and ranges of environmental footprints were used to construct confidence intervals for those relationships. A further strength is the use of empirical rather than modelled diets for the study. Nevertheless, the analyses also have potential weaknesses, among these was the simplification that all diets that met a certain number of recommendations were equally healthy (or unhealthy) regardless of which recommendations were being met, and the assumption that lower consumption of one food group or nutrient could not be compensated by higher consumption of other foods. Low inter-individual variance in diets associated with high adherence to some recommendations combined with relatively low overall intake (for example of red and processed meat) may also have resulted in low power to detect diet-health associations [41]. As for all studies measuring dietary intake, assessment is subject to measurement error. However, in the three datasets considered in this study, dietary intake data were collected using different methods, reducing the likelihood of type I errors across all included studies. Data on greenhouse gas emissions were obtained from diverse sources which used different methods and time periods. Data on water footprints were obtained from a single source, but this source used average crop water requirements and yields from the years 1996-2005, and these values may therefore have changed by the time of the UK dietary survey ~15 years later, resulting in some inaccuracies of food water footprints. We attempted to select data on greenhouse gas emissions from surveys with years corresponding to the years of the NDNS, but this was not always possible and therefore the same inaccuracies may affect the greenhouse gas footprints of the diets. Finally, due to data limitations it was not possible to assess both health and environmental footprints of diets within single datasets.

The EWG dietary recommendations are associated with better health outcomes and lower GHG emissions but are substantially different from the "planetary health diet" recently recommended [6], particularly in terms of red and processed meat consumption – with a much lower amount maximum amount of meat recommended in the latter. Our analysis suggests that considerable dietary shifts are required in UK dietary habits to meet the EWG recommendations, and that additional substantial changes would be needed to meet the more stringent planetary health diet recommendations. A major determinant of such shifts will be food prices [42, 43] and recent analysis has demonstrated that affordability of such diets may vary substantially [44]. Furthermore, it should be noted that an increasing proportion of plant-based foods for human consumption in the UK is imported from abroad [45]. Therefore, shifts in diets towards such foods, and no change in trading strategy, would further increase reliance on foreign production for resilient supply of plant-based foods. Moreover, an increasingly large proportion of these plant-based food imports originates from countries that are highly vulnerable to climate change (e.g. countries that are predicted to be highly water deficient by 2030). [32]. Care should be taken to avoid that dietary shifts towards EWG-adherence (and hence more plant-based diets) would result in substantial virtual water trade – away from water scarce countries - to supply the UK markets.

A fast-tracked nationwide shift towards adherence to the EWG will provide an essential step towards sustainable and healthy diets in the UK, to be followed by careful considerations on how to further improve sustainability beyond EWG adherence. Health services including family

For peer review only - http://bmjopen.bmj.com/site/about/guidelines.xhtml

doctors must play an active role in promoting adherence to the EWG recommendations to their patients [46] and thereby contribute directly to population health and environmental sustainability.

Funding & Role of Funder

This work was supported by the Wellcome Trust, Our Planet Our Health Programme (Sustainable and Health Food Systems - SHEFS [grant number 205200/Z/16/Z] & Livestock, Environment and People - LEAP [grant number 205212/Z/16/Z]); Cancer Research UK [grant numbers C8211/A19170; C570/A11692] and the UK Medical Research Council [grant numbers MR/M012190/1; MR/K02700X/1].

The views expressed in this publication are those of the authors and not necessarily those of the Public Health England or the Wellcome Trust. The final version of the report and ultimate decision to submit for publication was determined by the authors.

Conflict of Interest Declaration

The authors declare no conflict of interest

Authors' contributions:

PS - literature search, study design, data analysis, data interpretation, manuscript writing

RG - study design, data analysis, data interpretation, manuscript writing

KP - study design, data analysis, data interpretation, manuscript writing

AK - study design, data analysis, data interpretation, manuscript writing

CA - data analysis, commenting on manuscript

AB - data analysis, commenting on manuscript

TK - study design, commenting on manuscript

VB - study design, commenting on manuscript

AD – study design, commenting on manuscript

Patients and Public Involvement

This study is a secondary analysis of previously collected data. There was no involvement of patients or the public.

Transparency declaration

The lead author affirms that this manuscript is an honest, accurate, and transparent account of the study being reported; that no important aspects of the study have been omitted; and

For peer review only

that any discrepancies from the study as planned (and, if relevant, registered) have been explained.

Acknowledgements

The authors thank the women who have participated in the Million Women Study as well as the staff from the participating NHS breast screening centres. We also thank NHS Digital in England and the Information Services Division, NHS Scotland for linkage to data on cancers and deaths, and Public Health England for data based on information collected and quality assured by the Public Health England National Cancer Registration and Analysis Service, for which access was facilitated by the Office for Data Release. Data for this study include information collected and provided by the Office for National Statistics. Those who carried out the original collection and analysis of the data bear no responsibility for their further analysis or interpretation.

Data access policies for the Million Women Study are available via the study website [http://www.millionwomenstudy.org/]

We thank all participants in the EPIC-Oxford cohort for their invaluable contribution.

This research has been conducted using the UK Biobank Resource under application number 24494. All bona fide researchers can apply to use the UK Biobank resource for health related research that is in the public interest [https://www.ukbiobank.ac.uk/register-apply/]. We thank all participants, researchers and support staff who make the study possible.

We thank Paul Appleby and Aurora Perez-Cornago (Cancer Epidemiology Unit, Nuffield Department of Population Health, University of Oxford) for their valuable contribution to data preparation.

For peer review only

Data Availability Statement

All relevant data to the study are included in the article or uploaded as supplementary information.

Raw data from NDNS are available (upon request) from UK Data Service https://beta.ukdataservice.ac.uk/datacatalogue/series/series?id=2000033.

Raw data from UK Biobank, Million Women Study and EPIC Oxford are made available for selected research requests only. (UKB: https://www.ukbiobank.ac.uk/principles-of-access/; MWS: http://www.millionwomenstudy.org/files/07112018Datasharingpolicy.pdf; EPIC-Oxf: http://www.epic-oxford.org/data-access-sharing-and-collaboration/)

References

1. World Health Organization. *Malnutrition Fact Sheet - Key Facts*. 2018 February 2018 [cited 2019 November]; Available from: https://www.who.int/news-room/fact-sheets/detail/malnutrition.
2. World Health Organization. *WHO Nutrition Topics - Micronutrient Deficiencies*. 2019 [cited 2019 November]; Available from: https://www.who.int/nutrition/topics/ida/en/.
3. Mbow, H.O.P., et al., *Special Report on climate change, desertification, land degradation, sustainable land management, food security, and greenhouse gas fluxes in terrestrial ecosystems (SR2).* 2017.
4. Food and Agricultural Organization of the United Nations, *Water for Sustainable Food and Agriculture - A report produced for the G20 Presidency of Germany*. 2017, FAO: Rome.
5. Luo, T., R. Young, and P. Reig, *Aqueduct projected water stress country rankings.* Technical Note, 2015.
6. Willett, W., et al., *Food in the Anthropocene: the EAT–Lancet Commission on healthy diets from sustainable food systems.* The Lancet, 2019. **393**(10170): p. 447-492.
7. Knuppel, A., et al., *EAT-Lancet score and major health outcomes: the EPIC-Oxford study.* The Lancet, 2019. **394**(10194): p. 213-214.
8. Ministry of health of Brazil, *Dietary guidelines for the Brazilian population*. 2014, Ministry of Health of Brazil Brasília-DF (Brazil).
9. Fischer, C.G. and T. Garnett, *Plates, pyramids, and planets: developments in national healthy and sustainable dietary guidelines: a state of play assessment*. 2016: Food and Agriculture Organization of the United Nations.
10. National Health Services, *Health and Social Care Information Centre. Statistics on Obesity, Physical Activity and Diet, England, 2020*. 2020.
11. Hoekstra, A. and M. Mekonnen, *The water footprint of humanity, P. Natl. Acad. Sci., 109, 3232–3237*. 2012.
12. Hoekstra, A.Y. and M.M. Mekonnen, *Imported water risk: the case of the UK.* Environmental research letters, 2016. **11**(5): p. 055002.
13. Vanham, D., et al., *The water footprint of different diets within European sub-national geographical entities.* Nature Sustainability, 2018. **1**(9): p. 518-525.
14. Sandström, V., et al., *The role of trade in the greenhouse gas footprints of EU diets.* Global food security, 2018. **19**: p. 48-55.
15. Green, R., et al., *The potential to reduce greenhouse gas emissions in the UK through healthy and realistic dietary change.* Climatic Change, 2015. **129**(1-2): p. 253-265.
16. Public Health England. *The Eatwell Guide - Helping you eat a healthy, balanced diet*. 2018.

For peer review only

17. Aune, D., et al., *Fruit and vegetable intake and the risk of cardiovascular disease, total cancer and all-cause mortality—a systematic review and dose-response meta-analysis of prospective studies.* International journal of epidemiology, 2017. **46**(3): p. 1029-1056.
18. Levy, L. and A. Tedstone. *UK dietary policy for the prevention of cardiovascular disease*. in *Healthcare*. 2017. Multidisciplinary Digital Publishing Institute.
19. Cobiac, L.J., et al., *The Eatwell guide: modelling the health implications of incorporating new sugar and fibre guidelines.* PLoS One, 2016. **11**(12).
20. Aston, L.M., J.N. Smith, and J.W. Powles, *Impact of a reduced red and processed meat dietary pattern on disease risks and greenhouse gas emissions in the UK: a modelling study.* BMJ open, 2012. **2**(5): p. e001072.
21. Harris, F., et al., *The Water Footprint of Diets: A Global Systematic Review and Meta-analysis.* Advances in Nutrition, 2019.
22. Poore, J. and T. Nemecek, *Reducing food's environmental impacts through producers and consumers.* Science, 2018. **360**(6392): p. 987-992.
23. Scarborough, P., et al., *Dietary greenhouse gas emissions of meat-eaters, fish-eaters, vegetarians and vegans in the UK.* Climatic change, 2014. **125**(2): p. 179-192.
24. Carbon Trust, *The Eatwell Guide: A More Sustainable Diet*. 2016, The Carbon Trust London, UK.
25. Behrens, P., et al., *Evaluating the environmental impacts of dietary recommendations.* Proceedings of the National Academy of Sciences, 2017. **114**(51): p. 13412-13417.
26. Davey, G.K., et al., *EPIC–Oxford:lifestyle characteristics and nutrient intakes in a cohort of 33 883 meat-eaters and 31 546 non meat-eaters in the UK.* Public Health Nutrition, 2007. **6**(3): p. 259-268.
27. Collins, R., *What makes UK Biobank special?* The Lancet, 2012. **379**(9822): p. 1173-1174.
28. Green, J., et al., *Cohort profile: the million women study.* International journal of epidemiology, 2018. **48**(1): p. 28-29e.
29. Public Health England & the UK Food Standards Agency, *National Diet and Nutrition Survey (NDNS) wave 5-9 (2012-2017)*. 2019.
30. Scarborough, P., et al., *Eatwell Guide: modelling the dietary and cost implications of incorporating new sugar and fibre guidelines.* BMJ open, 2016. **6**(12): p. e013182.
31. Public Health England & the UK Food Standards Agency, *Appendix A: Dietary data collection and editing. In: Results from Years 7-8 (combined) of the Rolling Programme (2014/15 – 2015/16)*. 2018.
32. Food and Agricultural Organization of the United Nations, *FAOSTAT Statistical Database.* 2019: Rome.
33. Mekonnen, M.M. and A.Y. Hoekstra, *The green, blue and grey water footprint of crops and derived crop products.* Hydrology and Earth System Sciences, 2011. **15**(5): p. 1577-1600.
34. Department of Health (GB), *Dietary reference values for food energy and nutrients for the United Kingdom : Report of the Panel on Dietary Reference Values of the Committee on Medical Aspects of Food Policy*. 1991, London: H.M.S.O.
35. Milner, J., et al., *Health effects of adopting low greenhouse gas emission diets in the UK.* BMJ open, 2015. **5**(4): p. e007364.
36. Knuppel, A., et al., *EAT-Lancet score and major health outcomes: the EPIC-Oxford study.* The Lancet, 2019.
37. Cobiac, L.J. and P. Scarborough, *Modelling the health co-benefits of sustainable diets in the UK, France, Finland, Italy and Sweden.* European journal of clinical nutrition, 2019. **73**(4): p. 624.
38. Scarborough, P., et al., *Modelling the health impact of environmentally sustainable dietary scenarios in the UK.* European journal of clinical nutrition, 2012. **66**(6): p. 710.
39. Woodcock, J., et al., *Public health benefits of strategies to reduce greenhouse-gas emissions: urban land transport.* The Lancet, 2009. **374**(9705): p. 1930-1943.

For peer review only

40. Biesbroek, S., et al., *Reducing our environmental footprint and improving our health: greenhouse gas emission and land use of usual diet and mortality in EPIC-NL: a prospective cohort study.* Environmental Health, 2014. **13**(1): p. 27.

41. Schwingshackl, L., et al., *Food groups and risk of all-cause mortality: a systematic review and meta-analysis of prospective studies.* The American Journal of Clinical Nutrition, 2017. **105**(6): p. 1462-1473.

42. Green, R., et al., *The effect of rising food prices on food consumption: systematic review with meta-regression.* Bmj, 2013. **346**: p. f3703.

43. Scheelbeek, P.F., et al., *Potential impact on prevalence of obesity in the UK of a 20% price increase in high sugar snacks: modelling study.* bmj, 2019. **366**: p. l4786.

44. Hirvonen, K., et al., *Affordability of the EAT–Lancet reference diet: a global analysis.* The Lancet Global Health, 2019.

45. Scheelbeek, P., et al., *Resilience of UK fruit and vegetable supply: environmental threats to the supply of "5-a-day". Forthcoming*, 2020.

46. WONCA Working Party on the Environment, Planetary Health Alliance, and Clinicians for Planetary Health Working Group, *Declaration Calling for Family Doctors of the World to Act on Planetary Health*. 2019.

For peer review only

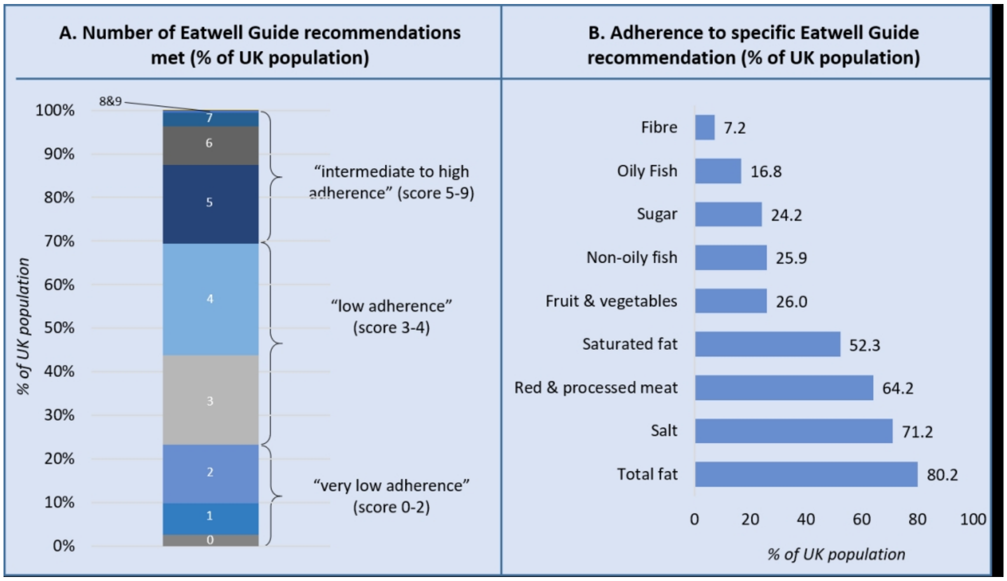

Figure 1: Adherence to the Eatwell Guide recommendations by the UK population - based on data from wave 5-9 of the National Dietary and Nutrition Survey (NDNS): A) total number of recommendations met by % of UK population; B) adherence to specific recommendations

236x135mm (150 x 150 DPI)

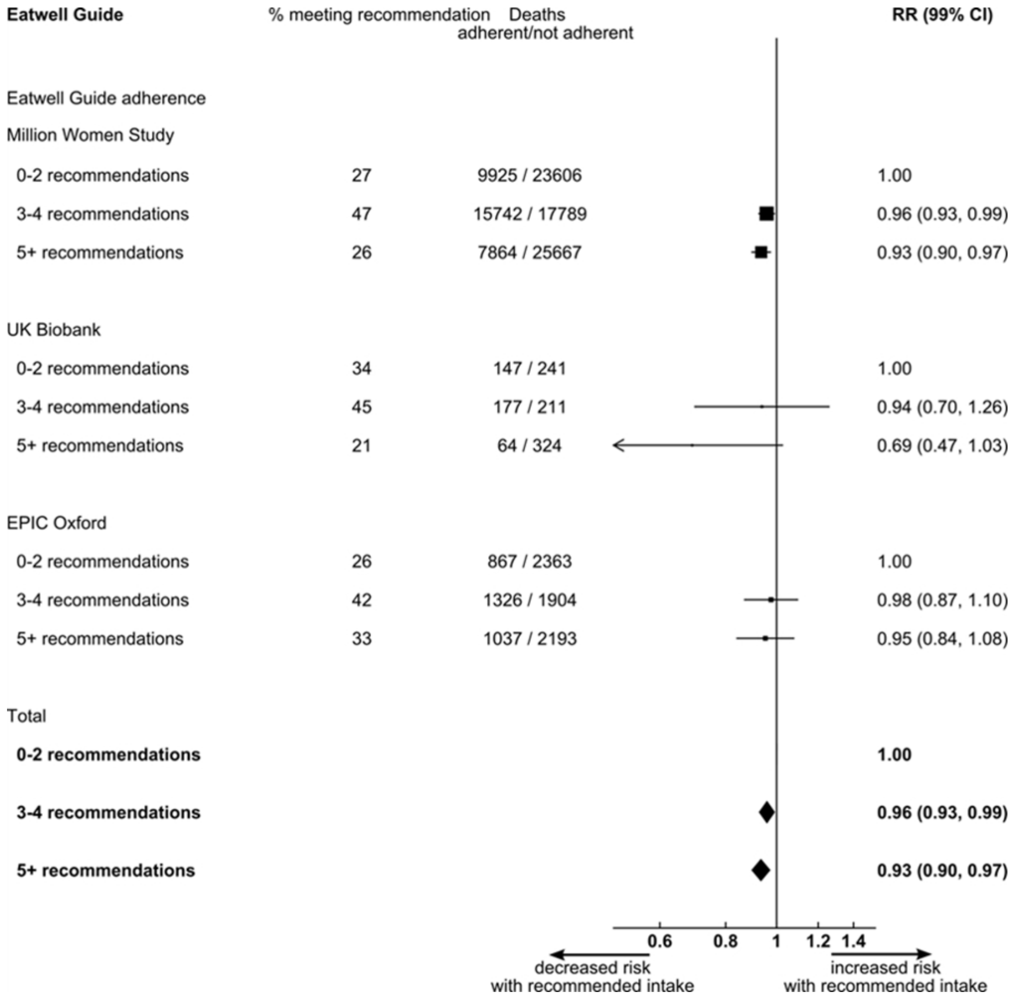

Figure 2: Forest plot showing the study specific (MWS, UKB & EPIC-Ox) and pooled mortality risk ratios comparing very poor adherence to EWG rocmmendations (score 0-2) with poor adherence (score 3-4) and intermediate to high adherence (score 5-9).

153x151mm (149 x 149 DPI)

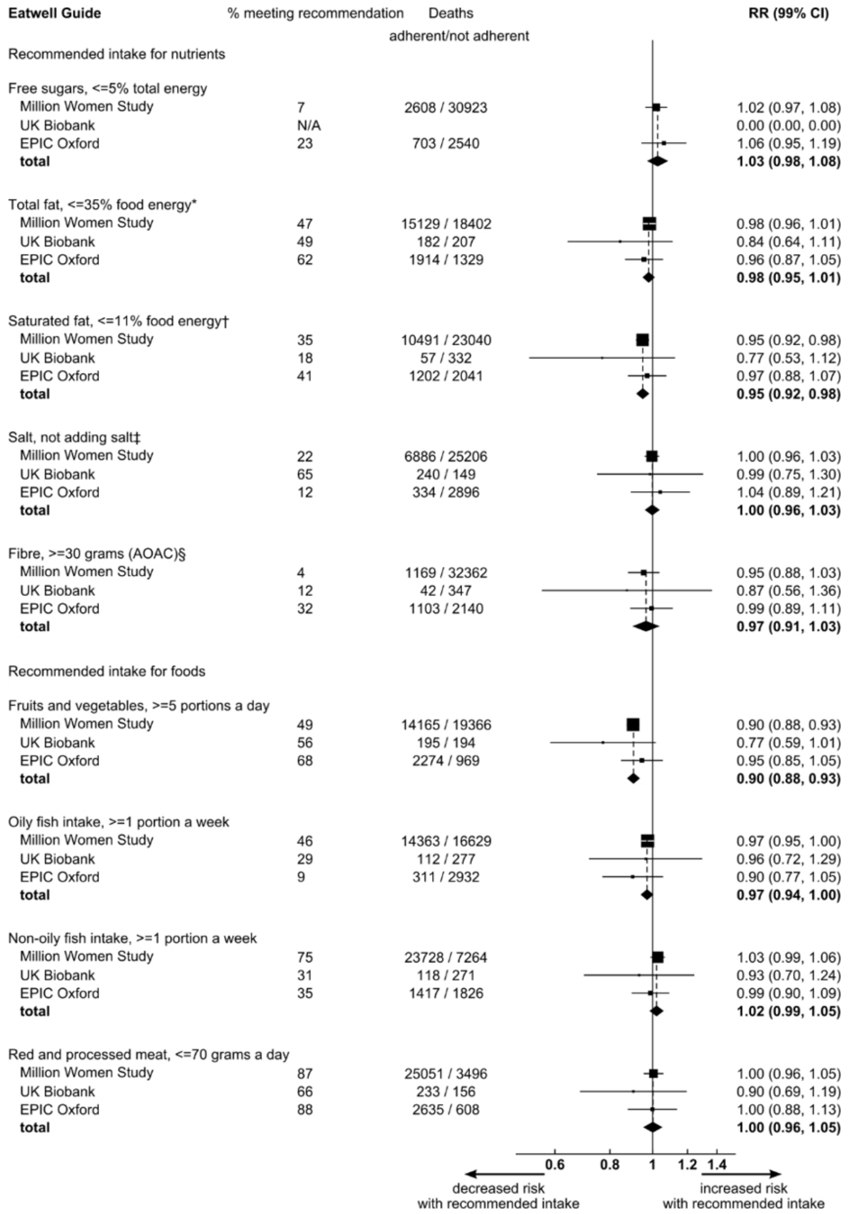

Figure 3: Mortality risk ratios for the association between adhering to specific EWG recommendations and total mortality. * Recommendation was based on food energy and was therefore adapted to ≥47% of total energy. *Adapted to <=33% of total energy. †Adapted to <=10% of total energy. ‡Information on salt intake was ascertained from the variable 'Never adding salt to food at the table or cooking' in the MWS and in the EPIC-Oxf study; and from the variable 'Not reporting having added salt to food (excluding during cooking)' in any of the WEBQs included in the UKB. §Fibre intake in the study was determined using the Englyst method and the recommendation was therefore adapted to >=22.6g/d of Englyst fibre.

143x206mm (150 x 150 DPI)

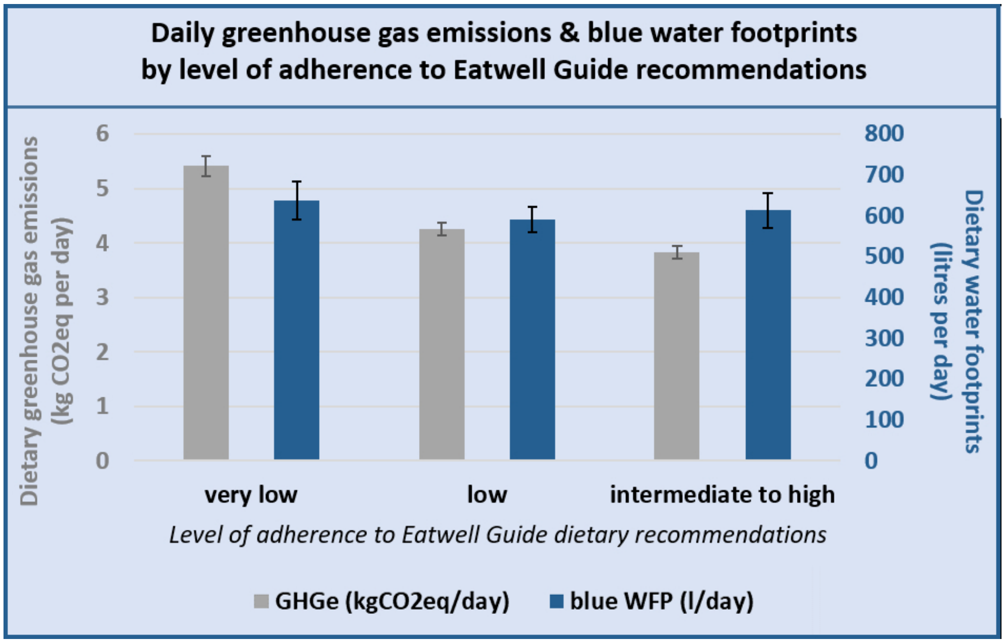

Figure 4: Average daily greenhouse gas emissions in kg CO2eq and average daily dietary water footprints comparing diets with very low (score 0-2), low (score 3-4) and intermediate to high adherence (score 5-9) to the Eatwell Guide dietary guidelines.

160x101mm (150 x 150 DPI)

Appendix

To: ***HEALTH IMPACTS AND ENVIRONMENTAL FOOTPRINTS OF DIETS THAT MEET THE EATWELL GUIDE RECOMMENDATIONS: ANALYSES OF MULTIPLE UK STUDIES***

For peer review only

Appendix 1 - Basic study descriptions for each cohort

Million Women Study

We used data from 464,078 participants of the Million Women Study. Participants were recruited from women (mean age 56 years) invited to the National Health service (NHS) breast cancer screening programme in England and Scotland between 1996 and 2001 [1]. Dietary intake was collected after an average of 3.3 years post recruitment using 130 semi-quantitative questions that were validated against a 7-day diet diary [2]. Nutrients were estimated by multiplying the frequency of consumption by portion size and nutrient composition of that item [3].

Total mortality was determined using death records obtained through linkage to centrally held NHS records. The Million Women Study protocol was approved by the Oxford and Anglia Multi-Centre Research Ethics committee. All participants provided written informed consent.

EPIC Oxford

We used data from 40,030 men and women (mean age 43 years) recruited throughout the UK (between 1993-2001) into the European Prospective Investigation into Cancer and Nutrition (EPIC)-Oxford cohort [4]. Dietary intake was collected using a validated 130-item semi-quantitative Food frequency questionnaire [5]. To estimate nutrient intakes, we multiplied frequencies of consumption by portion size and nutrient composition.

Total mortality was ascertained using death record linkage with the NHS Central register.

The EPIC-Oxford study protocol was approved by a Multi-Centre Research Ethics Committee (Scotland A Research Ethics Committee). All participants provided written informed consent.

UK Biobank

We used data from 53,614 middle-aged adults (mean age 56 years) participating in the UK Biobank who were recruited across the UK between 2006-2010 [6]. These were a subsample, that had completed a minimum of three 24-hour dietary recall questionnaires (the Oxford WebQ) [7]. Food and beverage frequency data, standard portion sizes and nutrient composition by item were multiplied to obtain nutrient intakes per day [8].

We linked participant data to the NHS Central register to obtain mortality information.

The UK Biobank was approved by the National Information Governance Board for Health and Social Care and the NHS North West Multi-Centre Research Ethics Committee. All participants provided written informed consent.

For peer review only

References

1. Green, J., et al., *Cohort profile: the million women study.* International journal of epidemiology, 2018. **48**(1): p. 28-29e.
2. Roddam, A.W., et al., *Reproducibility of a short semi-quantitative food group questionnaire and its performance in estimating nutrient intake compared with a 7-day diet diary in the Million Women Study.* Public Health Nutr, 2005. **8**(2): p. 201-13.
3. Key, T.J., et al., *Foods, macronutrients and breast cancer risk in postmenopausal women: a large UK cohort.* Int J Epidemiol, 2018: p. dyy238-dyy238.
4. Davey, G.K., et al., *EPIC–Oxford:lifestyle characteristics and nutrient intakes in a cohort of 33 883 meat-eaters and 31 546 non meat-eaters in the UK.* Public Health Nutrition, 2007. **6**(3): p. 259-268.
5. Bingham, S.A., et al., *Validation of dietary assessment methods in the UK arm of EPIC using weighed records, and 24-hour urinary nitrogen and potassium and serum vitamin C and carotenoids as biomarkers.* Int J Epidemiol, 1997. **26 Suppl 1**: p. S137-51.
6. Collins, R., *What makes UK Biobank special?* Lancet, 2012. **379**(9822): p. 1173-1174.
7. Greenwood, D.C., et al., *Validation of the Oxford WebQ online 24-hour dietary questionnaire using biomarkers.* Am J Epidemiol, 2019.
8. Bradbury, K.E., et al., *Dietary assessment in UK Biobank: an evaluation of the performance of the touchscreen dietary questionnaire.* J Nutr Sci, 2018. **7**: p. e6.

For peer review only

Appendix 2 – Eatwell Guide recommendations by age and sex

	5-6 years		7-10 years		11-14 years		15-18 years		19-64 years		65-74 years		75+ years	
Food group	Male	Female	Male	Female	Male	Female	Male	Female	Male	Female	Male	Female	Male	Female
Fruit & Vegetables (g/day)	400*	400g*	400g*	400g*	400g*	400g*	400g*	400g*	400g*	400g*	400g*	400g*	400g*	400g*
Oily Fish (g/day)	20g	20g	20g	20g	20g	20g	20g	20g	20g	20g	20g	20g	20g	20g
Other Fish (g/day)	20g	20g	20g	20g	20g	20g	20g	20g	20g	20g	20g	20g	20g	20g
Red and processed meat (g/day)	70	70	70	70	70	70	70	70	70	70	70	70	70	70
Fibre (g/day)	20**	20**	20**	20**	25**	25**	30**	30**	30**	30**	30**	30**	30**	30**
Salt (g/day)	3**	3**	5**	5**	6	6	6	6	6	6	6	6	6	6
Free Sugar (g/day)	19	19	24	24	30	30	30	30	30	30	30	30	30	30
Saturated fat (g/day)	18**	17 **	22**	20	30	20	30	20	30	20	30	20	30	20
Total fat (g/day)	58**	54**	71**	66**	97**	78**	97**	78**	97**	78**	91**	74**	89**	72**

**30g of dried fruit, max 150ml fruit juice or smoothie, and max 80g beans considered as one portion*

***Figures from PHE Government Dietary Recommendations document, derived from SACN. All other figures from the UK Eatwell Guide.*

Dietary recommendations of the Eatwell Guide

	Dietary recommendation and constraints
**Nutrients**	
Energy	2250 kcal (9414 MJ)*
Carbohydrates	≥50% of food energy
Free sugars	≤5% food energy
Fat	≤35% food energy
Saturated fat	≤11% food energy
Protein	≥14.5 & ≤15.5% of energy
Salt	≤ 6g/2363 mg/d sodium
Fibre (AOAC)	≥30g/d
**Foods**	

For peer review only

Fruits and vegetables†	≥5 portions a day
Fish	≥ 2 portions (2*140g) a week, one of which should be oily
Red and processed meat	≤70g/day

Table adapted from Public Health England (2016), Table 2.
**Energy intake recommendation assumes mixed population average.*
†Fruit and vegetable intake includes a maximum of: 1 portion of juice (from fruit / vegetable juice or that in a smoothie); 1 portion of beans; (portion sizes: 30g dried fruit; combined total of 150ml of fruit and / or vegetable juice and / or smoothie; 80g all other fruits & vegetables).

For peer review only

Appendix 3 – Cohort specific adjustments and cut-off values

Million Women Study

Associations were stratified by region and adjusted for smoking (never smoked, past smoker, current smoker <10 cigarettes smoked per day, current smoker 10-19 cigarettes smoked per day, current smoker ≥20 cigarettes smoked per day, current smoker and unknown number of cigarettes smoked per day), deprivation (tertiles), alcohol (0, 1-6, 7-14, ≥15 drinks per week), height cm (<155 ,155-164, ≥165), BMI kg/m^2 (<20, 20-24, 25-29, ≥30), strenuous exercise (<1 per week,≥1 per week), Hormone replacement therapy (HRT) use (never, past, current), educational attainment (none, technical/secondary/tertiary), self-reported hypertension (yes, no), and energy intake (quintiles).

EPIC Oxford

Associations were stratified by sex, region, and method of recruitment and adjusted for smoking (never smoked, past smoker, current smoker <10 cigarettes smoked per day, current smoker 10-19 cigarettes smoked per day, current smoker ≥20 cigarettes smoked per day, current smoker and unknown number of cigarettes smoked per day), deprivation (tertiles, unknown), alcohol intake (<1, 1-7 (ref.), 8-15, ≥16 grams per day, unknown), height cm (sex-specific tertiles, unknown), BMI kg/m^2(<20, 20-24, 25-29, ≥30), physical activity (inactive, low, moderate, high activity, unknown), HRT use ever (yes, no, unknown), educational attainment (national examination at age 16, national examination at ages 17-18, vocational qualification, , degree, unknown), self-reported high blood pressure (no, yes, unknown), and energy intake (sex-specific quintiles)

UK Biobank

Associations were stratified by sex and region and adjusted for smoking (never smoked, past smoker, current smoker <10 cigarettes smoked per day, current smoker 10-19 cigarettes smoked per day, current smoker ≥20 cigarettes smoked per day, current smoker and unknown number of cigarettes smoked per day), deprivation (tertiles, unknown), alcohol intake (none, <1, 1-<10, 10-<20, ≥20 grams per day, unknown), height cm (sex-specific tertiles, unknown), BMI kg/m^2 (<20, 20-24, 25-29, ≥30), physical activity (<10 excess METs, 10-<50 excess METs, ≥50 excess METs, unknown), qualification (national examination at age 16, national examination at ages 17-18, vocational qualification, college or university degree, other, unknown), HRT (never, past, current, unknown), self-reported high blood pressure (no, yes, unknown), and energy intake (sex-specific quintiles)

For peer review only

Appendix 4 – Results sensitivity analysis mortality RR

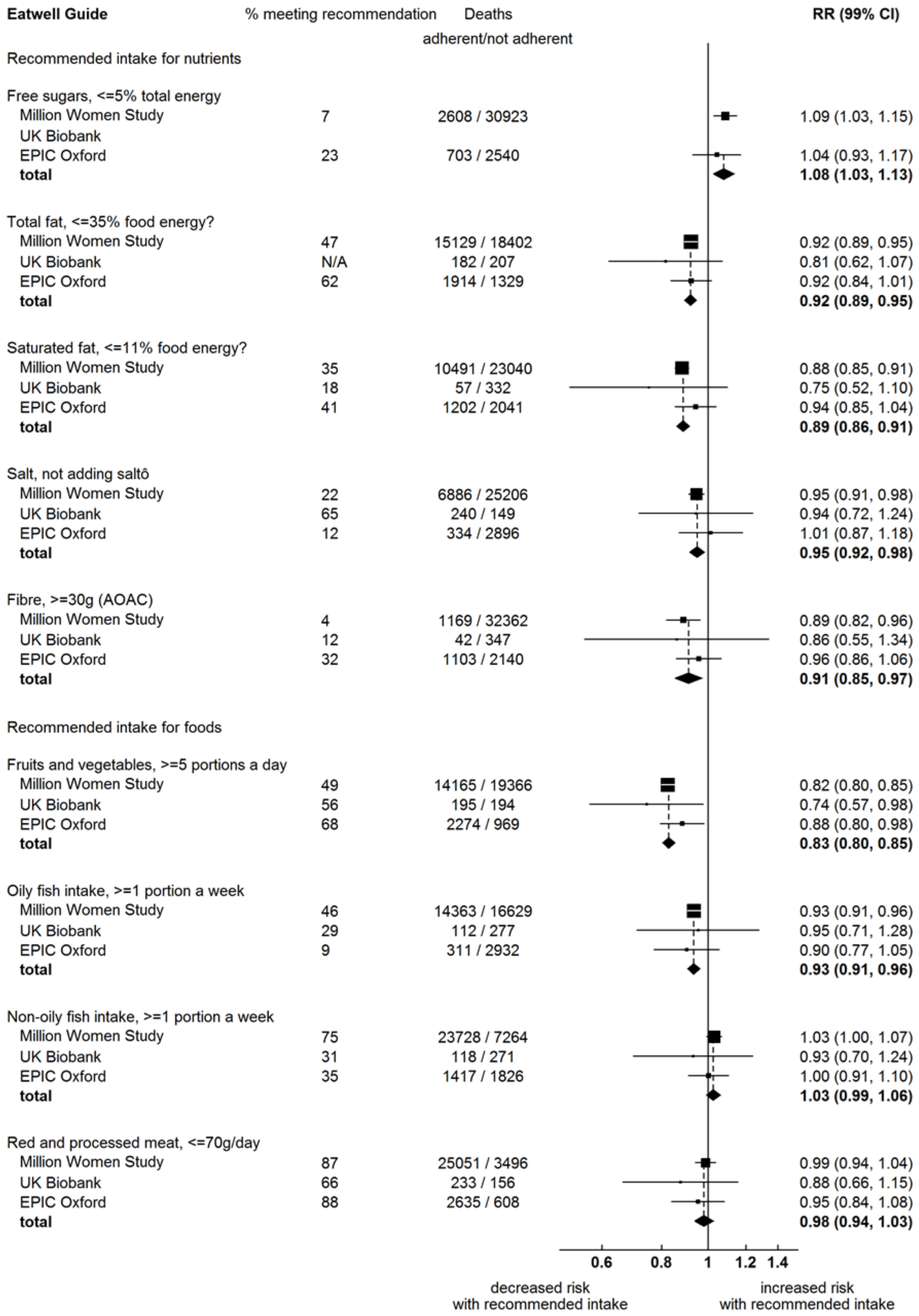

Figure S 1 Risk ratios for the association between some recommendations of the Eatwell Guide and total mortality <u>without adjustment for smoking</u>

Eatwell Guide	% meeting recommendation	Deaths adherent/not adherent	RR (99% CI)
Recommended intake for nutrients			
Free sugars, <=5% total energy			
Million Women Study	7	2608 / 30923	0.99 (0.90, 1.08)
UK Biobank			
EPIC Oxford	23	703 / 2540	1.02 (0.86, 1.20)
**total**			**1.00 (0.92, 1.08)**
Total fat, <=35% food energy?			
Million Women Study	47	15129 / 18402	1.01 (0.97, 1.05)
UK Biobank	N/A	182 / 207	0.63 (0.41, 0.96)
EPIC Oxford	62	1914 / 1329	0.94 (0.82, 1.08)
**total**			**1.00 (0.96, 1.04)**
Saturated fat, <=11% food energy?			
Million Women Study	35	10491 / 23040	0.98 (0.93, 1.02)
UK Biobank	18	57 / 332	0.62 (0.33, 1.17)
EPIC Oxford	41	1202 / 2041	1.02 (0.88, 1.17)
**total**			**0.98 (0.94, 1.02)**
Salt, not adding saltô			
Million Women Study	22	6886 / 25206	1.02 (0.97, 1.07)
UK Biobank	65	240 / 149	1.09 (0.71, 1.70)
EPIC Oxford	12	334 / 2896	1.08 (0.88, 1.32)
**total**			**1.02 (0.98, 1.07)**
Fibre, >=30g (AOAC)			
Million Women Study	4	1169 / 32362	1.00 (0.90, 1.11)
UK Biobank	12	42 / 347	0.85 (0.43, 1.67)
EPIC Oxford	32	1103 / 2140	1.00 (0.86, 1.16)
**total**			**0.99 (0.91, 1.08)**
Recommended intake for foods			
Fruits and vegetables, >=5 portions a day			
Million Women Study	49	14165 / 19366	0.91 (0.87, 0.95)
UK Biobank	56	195 / 194	0.80 (0.53, 1.21)
EPIC Oxford	68	2274 / 969	0.93 (0.79, 1.08)
**total**			**0.91 (0.87, 0.94)**
Oily fish intake, >=1 portion a week			
Million Women Study	46	14363 / 16629	0.97 (0.93, 1.01)
UK Biobank	29	112 / 277	1.19 (0.78, 1.82)
EPIC Oxford	9	311 / 2932	0.92 (0.74, 1.15)
**total**			**0.97 (0.93, 1.01)**
Non-oily fish intake, >=1 portion a week			
Million Women Study	75	23728 / 7264	1.01 (0.96, 1.06)
UK Biobank	31	118 / 271	0.56 (0.34, 0.92)
EPIC Oxford	35	1417 / 1826	0.99 (0.86, 1.13)
**total**			**1.00 (0.95, 1.05)**
Red and processed meat, <=70g/day			
Million Women Study	87	25051 / 3496	1.03 (0.96, 1.10)
UK Biobank	66	233 / 156	0.95 (0.62, 1.46)
EPIC Oxford	88	2635 / 608	0.99 (0.82, 1.20)
**total**			**1.02 (0.96, 1.09)**

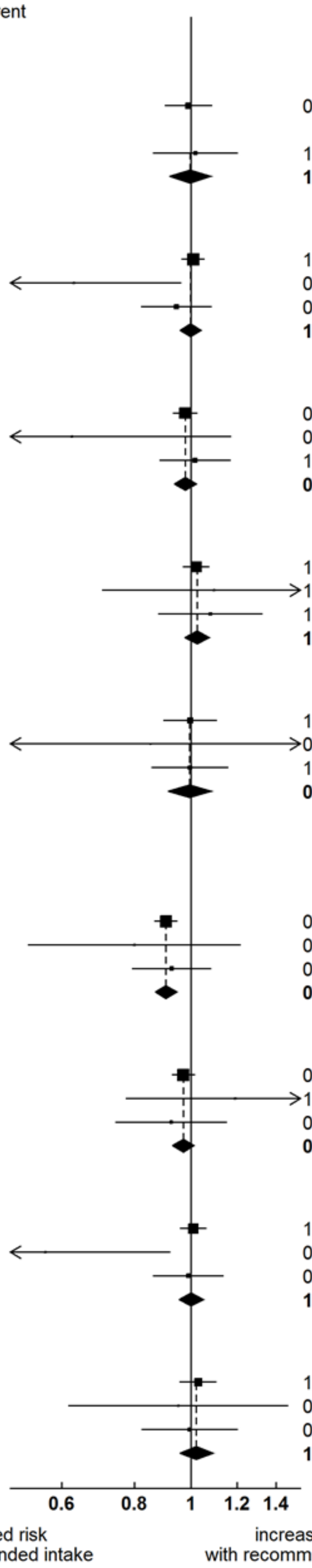

Figure S 2 Risk ratios for the association between some recommendations of the Eatwell Guide and total mortality <u>in never smokers</u>

Risk ratios for the association some recommendations of the Eatwell Guide and total mortality

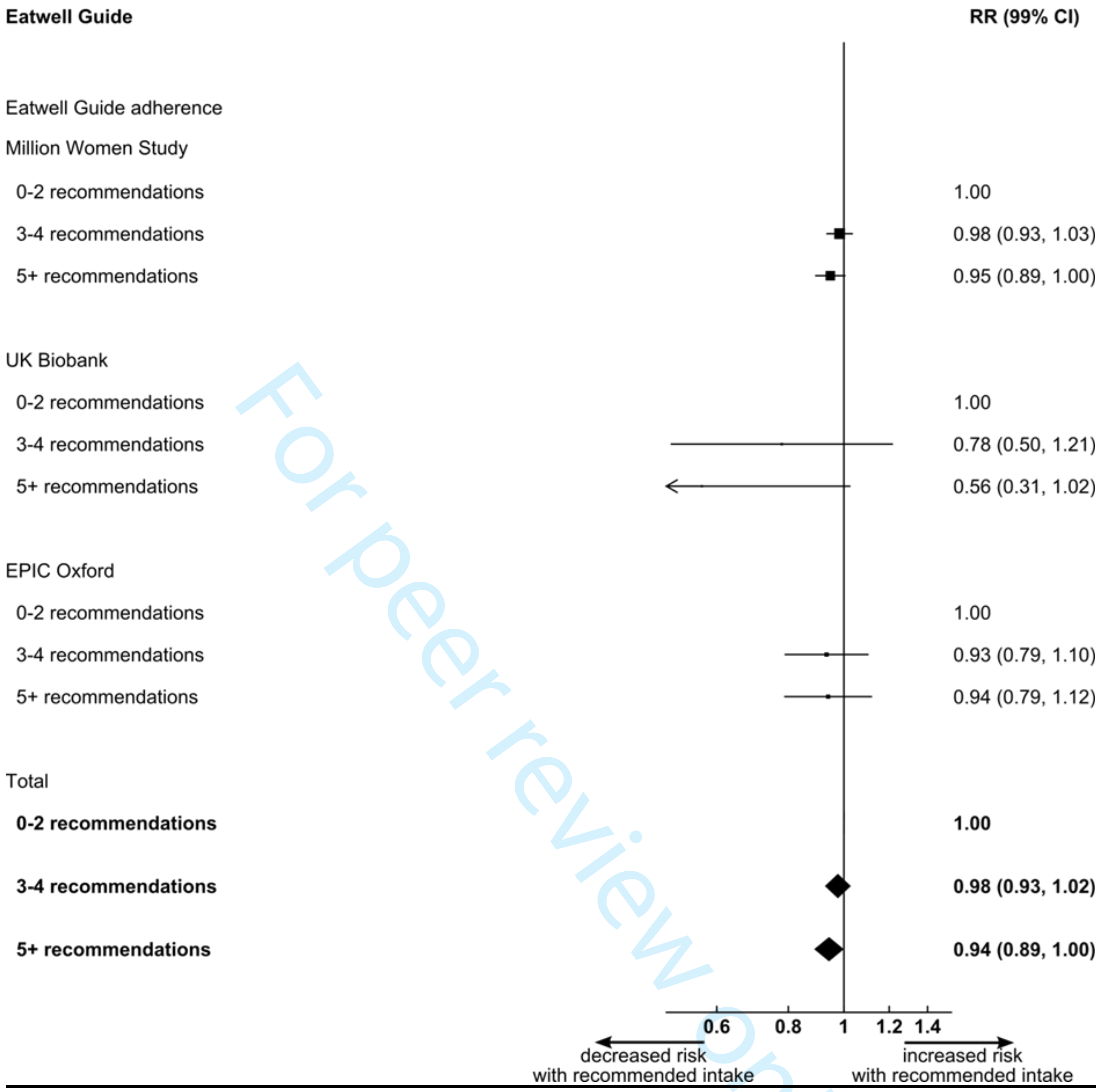

Figure S 3 Risk ratios for the association between the Eatwell Guide three level score and total mortality in never smokers

For peer review only

Appendix 5 – Detailed GHG emission and WFP calculations

Data from NDNS were matched to food-specific GHG emissions and blue water footprint estimates from previous studies using 173 SubFoodGroups available in the NDNS dataset. Where SubFoodGroups contained a number of foods with differing environmental footprints, weighted means according to consumption were used, and similarly where SubFoodGroups were composite foods including many ingredients (e.g. meat pies) recipe data were used to estimate the mean proportions of ingredients contained within the composite food. Recipe data were obtained from Sainsbury's (www.sainsbury.co.uk). Composite food and weighting data are available on request.

A number of additional SubFoodGroup categories were created in order to distinguish between categories where environmental footprints would be expected to differ greatly. These were:

- SubFoodGroup **6A** (wheat based breakfast cereals), **6B** (chocolate breakfast cereals), **6C** (oat based breakfast cereals), **6D** (maize based breakfast cereals), and **6E** (rice based breakfast cereals) were added in MainFoodGroup 6 (other breakfast cereals)
- SubFoodGroup **5A** (wheat based high fibre breakfast cereals), **5B** (chocolate high fibre breakfast cereals), **5C** (oat based high fibre breakfast cereals), and **5D** (rice based high fibre breakfast cereals) were added in MainFoodGroup 5 (high fibre breakfast cereals) and SubFoodGroup 5R was renamed (other high fibre breakfast cereals)
- SubFoodGroup **7C** (biscuits chocolate retail) was added in MainFoodGroup 7 (biscuits).
- SubFoodGroup **8F** (cakes chocolate) was added in MainFoodGroup 8 (buns cakes pastries and fruit pies)
- SubFoodGroups **13C** (non-dairy cream), **13D** (almond milk), **13E** (soya milk), **13F** (other non-dairy milk) and **13G** (chocolate milk) were added in MainFoodGroup 13 (other milk and cream) – Elmlea is not included in non dairy cream as it contains buttermilk
- SubFoodGroup **14C** (dairy free cheese) was added in MainFoodGroup 14 (cheese)
- SubFoodGroup **15E** (non dairy desserts) was added in MainFoodGroup 15 (yoghurt fromage frais and dairy desserts)
- SubFoodGroup **19B** (less than 1% dairy low fat spread) was added in MainFoodGroup 19 (low fat spread)
- SubFoodGroup **20A** (block margarine) was included in the dairy-free categories
- SubFoodGroup **20C** (other cooking fats and oils not PUFA) was renamed (other vegetable fats and oils) and a new SubFoodGroup **20B** (animal fats) was added in MainFoodGroup 20 (other margarine fats and oils)
- SubFoodGroup **21C** (less than 1% dairy reduced fat spread) was added in MainFoodGroup 21 (reduced fat spread)
- SubFoodGroup 37C (baked beans with sausages), moved to SubFoodGroup **25A** (manufactured pork products including ready meals) and categorised into MainFoodGroup 25 (pork products).
- SubFoodGroup **53A** (non dairy ice cream) was added in MainFoodGroup 53 (ice cream)

Codes 13A (infant formula), 54B (evening primrose oil and other plant oils), 54D (folic acid), 54E (iron only or with vitamin C), 54F (calcium only or with vitamin D), 54G (vitamins (two or

For peer review only

more including multivitamins), no minerals), 54H (minerals (two or more including multimins), no vitamins), 54I (vitamins and minerals (including multivits and minerals)), 54J (non-nutrient supplements including herbal), 54K (other nutrient supplements), 54L (vitamin C only), 54M (other single vitamins or minerals), 54M (cod liver oil and other fish oils), 54P (multivitamins and/or minerals with omega 3),

SubFood GroupCode	Food Group Name	Mean Greenhouse Gas emissions (kg) per kg food	Mean blue water footprint (litres) per kg food
1C	Pizza	3.51	369
1D	Pasta manufactured products and ready meals	1.00	0
1E	Other pasta including homemade dishes	1.00	0
1F	Rice manufactured products and ready meals	3.13	1071
1G	Other rice including homemade dishes	3.13	1071
1R	Other cereals	1.18	103
2R	White bread (not high fibre, not multiseed)	0.97	0
3R	Wholemeal bread	0.97	0
4R	Other bread	0.97	0
5A	Wheat based high fibre breakfast cereals	1.40	229
5B	Chocolate high fibre breakfast cereals	2.68	62
5C	Oat based high fibre breakfast cereals	1.41	109
5R	Other high fibre breakfast cereals	1.27	2
6A	Wheat based breakfast cereals	1.40	0
6B	Chocolate breakfast cereals	3.03	779
6C	Oat based breakfast cereals	1.41	109
6D	Maize based breakfast cereals	2.64	124
6E	Rice based breakfast cereals	2.85	1009
6R	Other breakfast cereals (not high fibre)	1.27	2
7A	Biscuits manufactured / retail	1.80	143
7B	Biscuits homemade	2.30	201
7C	Biscuits chocolate retail	8.14	135
8B	Fruit pies manufactured	0.95	86
8C	Fruit pies homemade	1.11	91
8D	Buns cakes and pastries manufactured	1.08	66
8E	Buns cakes and pastries homemade	3.31	34
8F	Cakes chocolate	3.11	126
9C	Cereal based milk puddings manufactured	2.00	170
9D	Cereal based milk puddings homemade	2.00	170

For peer review only

9E	Sponge puddings manufactured	1.79	17
9F	Sponge puddings homemade	1.79	17
9G	Other cereal based puddings manufactured	4.00	26
9H	Other cereal based puddings homemade	0.08	1
10R	Whole milk	1.53	28
11R	Semi skimmed milk	1.53	24
12R	Skimmed milk	1.53	24
13B	Cream (including imitation cream)	4.89	44
13C	Non dairy cream alternative	2.64	6
13D	Almond milk	0.99	73
13E	Soya milk	0.88	2
13F	Other non-dairy milk	2.65	57
13G	Chocolate milk	1.53	24
13R	Other milk	1.80	109
14A	Cottage cheese	15.00	132
14B	Cheddar cheese	8.87	132
14C	Dairy free cheese alternative	1.76	4
14R	Other cheese	8.87	59
15B	Yoghurt	2.00	31
15C	Fromage frais and dairy desserts	2.00	27
15D	Dairy desserts homemade	1.32	81
15E	Non dairy desserts	2.05	94
16C	Manufactured egg products including ready meals	3.51	4
16D	Other eggs and egg dishes including homemade	4.70	39
17R	Butter	9.00	194
18B	Polyunsaturated oils	3.59	235
19A	Polyunsaturated low fat spread	4.19	127
19B	Less than 1% dairy low fat spread	4.19	144
19R	Low fat spread not polyunsaturated	3.95	127
20A	Block margarine	4.19	144
20B	Animal fats	14.31	162
20C	Other vegetable fats and oils	4.65	853
21A	Reduced fat spread (polyunsaturated)	4.35	146
21B	Reduced fat spread (not polyunsaturated)	4.35	146
21C	Less than 1% dairy reduced fat spread	4.19	144

For peer review only

22A	Ready meals / meal centres based on bacon and ham	7.47	304
22B	Other bacon and ham including homemade dishes	10.70	321
23A	Manufactured beef products including ready meals	10.40	127
23B	Other beef and veal including homemade recipe dishes	16.50	205
24A	Manufactured lamb products including ready meals	30.48	275
24B	Other lamb including homemade recipe dishes	50.00	446
25A	Manufactured pork products including ready meals	8.85	321
25B	Other pork including homemade recipe dishes	10.00	293
26A	Manufactured coated chicken / turkey products	3.12	80
27A	Manufactured chicken products including ready meals	3.50	38
27B	Other chicken / turkey including homemade recipe dishes	3.50	38
28R	Liver and dishes	8.85	59
29R	Burgers and kebabs purchased	34.80	252
30A	Ready meals based on sausages	4.80	139
30B	Other sausages including homemade dishes	8.85	321
31A	Manufactured meat pies and pastries	8.63	209
31B	Homemade meat pies and pastries	13.96	251
32A	Other meat products manufactured including ready meals	11.50	594
32B	Other meat including homemade recipe dishes	2.84	37
33R	White fish coated or fried	3.36	0
34C	Manufactured white fish products including ready meals	4.55	0
34D	Other white fish including homemade dishes	4.55	0
34E	Manufactured shellfish products including ready meals	24.00	142
34F	Other shellfish including homemade dishes	24.00	142
34G	Manufactured canned tuna products including ready meals	4.55	0
34H	Other canned tuna including homemade dishes	4.55	0

For peer review only

35A	Manufactured oily fish products including ready meals	4.55	256
35B	Other oily fish including homemade dishes	4.55	256
36A	Carrots raw	1.28	0
36B	Salad and other raw vegetables	0.68	16
36C	Tomatoes raw	0.96	36
37A	Peas not raw	2.55	13
37B	Green beans not raw	0.50	40
37C	Baked beans	2.15	428
37D	Leafy green vegetables not raw	0.73	23
37E	Carrots not raw	1.28	0
37F	Tomatoes not raw	0.96	36
37I	Beans and pulses including ready meals and homemade dishes	1.51	21
37K	Meat alternatives including ready meals and homemade dishes	3.60	200
37L	Other manufactured vegetable products including ready meals	1.60	7
37M	Other vegetables including homemade dishes	0.58	39
38A	Chips purchased including take away	1.45	30
38C	Other manufactured potato products fried / baked	1.46	17
38D	Other fried / roast potatoes including homemade dishes	3.08	17
39A	Other potato products and dishes manufactured	1.20	46
39B	Other potatoes including homemade dishes	1.20	19
40A	Apples and pears not canned	0.70	52
40B	Citrus fruit not canned	0.40	93
40C	Bananas	0.90	49
40D	Canned fruit in juice	1.32	218
40E	Canned fruit in syrup	1.32	218
40R	Other fruit not canned	1.63	82
41A	Sugar	0.32	1
41B	Preserves	2.96	206
41R	Sweet spreads fillings and icing	7.14	269
42R	Crisps and savoury snacks	2.47	92
43R	Sugar confectionery	0.32	1
44R	Chocolate confectionery	1.07	78

For peer review only

45R	Fruit juice	1.01	157
47A	Liqueurs	1.00	1
47B	Spirits	1.00	1
48A	Wine	1.00	1
48B	Fortified wine	1.00	1
48C	Low alcohol and alcohol free wine	1.00	1
49A	Beers and lagers	3.80	14
49B	Low alcohol and alcohol free beer and lager	3.80	14
49C	Cider and perry	0.08	1
49D	Low alcohol and alcohol free cider and perry	3.80	1
49E	Alcoholic soft drinks	0.80	28
50A	Beverages dry weight	1.80	119
50C	Soup manufactured / retail	1.25	27
50D	Soup homemade	0.47	6
50E	Nutrition powders and drinks	0.00	0
50R	Savoury sauces pickles gravies and condiments	1.54	27
51A	Coffee (made up weight)	0.79	1955
51B	Tea (made up weight)	0.33	221
51C	Herbal tea (made up weight)	0.40	1
51D	Bottled water still or carbonated	0.40	1
51R	Tap water only	1.00	1
52A	Commercial toddlers drinks	0.00	0
52R	Commercial toddlers foods	0.00	0
53A	Non dairy ice cream	2.05	94
53R	Ice cream	3.82	44
55R	Artificial sweeteners	3.20	487
56R	Nuts and seeds	1.57	1415
57A	Soft drinks not low calorie concentrated	0.40	1
57B	Soft drinks not low calorie carbonated	0.40	1
57C	Soft drinks not low calorie rtd still	0.40	1
58A	Soft drinks low calorie concentrated	0.40	1
58B	Soft drinks low calorie carbonated	0.40	1
58C	Soft drinks low calorie rtd still	0.40	1
59R	Brown granary and wheatgerm bread	0.97	0
60R	1% fat milk	1.53	23
61R	Smoothies 100% fruit and / or juice	1.05	54

For peer review only

For peer review only

Appendix 6 - References for GHG and Blue WF figures

Amienyo D, Gujba H, Stichnothe H and Azapagic A (2013). Life cycle environmental impacts of carbonated soft drinks. *International Journal of Life Cycle Assessment* 18(1): 77-92.

Azapagic A, Bore J, Cheserek B, Kamunya S and Elbehri A (2016). The global warming potential of production and consumption of Kenyan tea. *Journal of Cleaner Production* 112(5): 4031-4040.

Bartzas G, Vamvuka D and Kommitsas K (2017). Comparative life cycle assessment of pistachio, almond and apple production. *Information Processing in Agriculture* 4(3): 188-198.

Bronmer E, Stratmann B and Quack D (2011). Environmental impacts of different methods of coffee preparation. *International Journal of Consumer Studies* 35(2): 212-220.

Buschspies B, Tolle SJ and Jungbluth N (2011). *Life Cycle Assessment of High-Sea Fish and Salmon Aquaculture.* ESU Services, Uster.

Carbon Trust (2017). *Report to Marlow Foods on Product Carbon Footprint Certification.*

Cichorowski G, Joa B, Hottenroth H and Schmidt M (2015). Scenario analysis of life cycle greenhouse gas emissions of Darjeeling tea. *International Journal of Life Cycle Assessment* 20(4): 426-439.

Clune S, Crossin E and Verghese K (2017). Systematic review of greenhouse gas emissions for different fresh food categories. *Journal of Cleaner Production* 140(2): 766-783.

Djekic I, Miocinovic J, Tomasevic I, Smigic N and Tomic N (2014). Environmental life-cycle assessment of various dairy products. *Journal of Cleaner Production* 68: 64-72.

Doublet G and Jungbluth N (2010). *Life Cycle Assessment of Drinking Darjeeling Tea.* ESU-Services, Uster.

Espinoza-Orias N, Stichnothe H and Azapagic A (2011). The carbon footprint of bread. *International Journal of Life Cycle Assessment* 16(4): 351-365.

Fantin V, Buttol P, Pergreffi R and Masoni P (2012). Life cycle assessment of Italian high quality milk production. A comparison with an EPD study. *Journal of Cleaner Production* 28: 150-159.

Finnegan W, Goggins J, Clifford E and Zhan X (2017). Global warming potential associated with dairy products in the Republic of Ireland. *Journal of Cleaner Production* 163: 262-273.

Frankowska A, Jeswani HK and Azapagic A (2019). Environmental impacts of vegetables consumption in the UK. *Science of the Total Environment* 682: 80-105.

Gonzalez-Garcia S, Gomez-Fernandez Z, Dias AC, Feijoo G, Moreira MT and Arroja L (2014). Life Cycle Assessment of broiler chicken production: a Portuguese case study. *Journal of Cleaner Production* 74: 125-134.

Huerta AR, Guereca LP and Lozano MSR (2016). Environmental impact of beef production in Mexico through life cycle assessment. *Resources, Conservation and Recycling* 109: 44-53.

Humbert S, Loerencik Y, Rossi V, Margni M and Jolliet O (2009). Life cycle assessment of spray dried soluble coffee and comparison with alternatives (drip filter and capsule espresso). *Journal of Cleaner Production* 17(15): 1351-1358.

For peer review only

Iriarte A, Almeida MG and Villalobos P (2014). Carbon footprint of premium quality export bananas: case study in Ecuador, the world's largest exporter. *Science of the Total Environment* 472: 1082-1088.

Jeswani HK, Burkinshaw R and Azapagic A (2015). Environmental sustainability issues in the food-energy-water nexus: breakfast cereals and snacks. *Sustainable Production and Consumption* 2: 17-28.

Jungbluth N (2006). *Comparison of the Environmental Impact of Tap Water vs. Bottled Mineral Water.* Swiss Gas and Water Association, Uster.

Kendall A, Marvinney E, Brodt S and Zhu W (2015). Life cycle-based assessment of energy use and greenhouse gas emissions in almond production, part I: analytical framework and baseline results. *Journal of Industrial Ecology* 19(6): 1008-1018.

Konstantas A, Stamford L and Azapagic A (2019). Economic sustainability of food supply chains: life cycle costs and value added in the confectionery and frozen desserts sectors. *Science of the Total Environment* 670: 902-914.

Konstantas A, Jeswani HK, Stamford L and Azapagic A (2018). Environmental impacts of chocolate production and consumption in the UK. *Food Research International* 106: 1012-1025.

Kristensen T, Soegaard K, Eriksen J and Mogensen L (2015). Carbon footprint of cheese produced on milk from Holstein and Jersey cows fed hay differing in herb content. *Journal of Cleaner Production* 101: 229-237.

Leinonen I, Williams AG, Wiseman J, Guy J and Kyriazakis I (2012). Predicting the environmental impacts of chicken systems in the United Kingdom through a life cycle assessment: Broiler production systems. *Poultry Science* 91(1): 8-25.

McCarty JA, Sandefur HN, Matlock M, Thoma G and Kim D (2014). Life cycle assessment of greenhouse gas emissions associated with production and consumption of peanut butter in the US. *Transactions of the ASABE* 57(6): 1741-1750.

Mekonnen MM and Hoekstra AY (2011). The green, blue and grey water footprint of crops and derived crop products. *Hydrology and Earth System Sciences* 15(5): 1577-1600.

Mekonnen MM and Hoekstra AY (2012). A global assessment of the water footprint of farm animal products. *Ecosystems* 15(3): 401-415.

Nguyen TLT, Hermansen JE and Mogensen E (2010). Environmental consequences of different beef production systems in the EU. *Journal of Cleaner Production* 18(8): 756-766.

Nilsson K, Flysjo A, Davis J, Sim S, Unger N and Bell S (2010). Comparative life cycle assessment of margarine and butter consumed in the UK, Germany and France. *International Journal of Life Cycle Assessment* 15(9): 916-926.

Nilsson K, Sund V and Floren B (2011). *The Environmental Impact of the Consumption of Sweets, Crisps and Soft Drinks.* Nordic Council of Ministers, Copenhagen.

Noya I, Aldea X, Gasol CM, Gonzalez-Garcia S, Amores MJ, Colon J, Ponsa S, Roman I, Rubio MA, Casas E, Moreira MT and Boschmonart-Rives J (2016). Carbon and water footprint of pork supply chain in Catalonia: From feed to final products. *Journal of Environmental Management* 171: 133-143.

For peer review only

Opio C, Gerber P, Mottet A, Falcucci A, Tempio G, MacLeod M, Vellinga T, Henderson B and Steinfeld H (2013). *Greenhouse gas emissions from ruminant supply chains – A global life cycle assessment*. Food and Agriculture Organization of the United Nations (FAO), Rome.

Pahlow M, van Oel PR, Mekonnen MM and Hoekstra AY (2015). Increasing pressure on freshwater resources due to terrestrial feed ingredients for aquaculture production. *Science of the Total Environment* 536: 847-857.

Poore J and Nemecek T (2018). Reducing food's environmental impacts through producers and consumers. *Science* 360: 987-992.

Prudencio da Silva V, van der Werf HMG, Soares SR and Corson MS (2014). Environmental impacts of French and Brazilian broiler chicken scenarios: An LCA approach. *Journal of Environmental Management* 133: 222-231.

Recanti F, Marveggio D and Dotelli G (2018). From beans to bar: a life cycle assessment towards sustainable chocolate supply chain. *Science of the Total Environment* 613-614: 1013-1023.

Recchia L, Cappelli A, Cini E, Pegna FG and Boncinelli P (2019). Environmental sustainability of pasta production chains: an integrated approach for comparing local and global chains. *Resources* 8(1): 56.

Scarborough P, Appleby PN, Mizdrak A, Briggs ADM, Travis RC, Bradbury KE and Key TJ (2014). Dietary greenhouse gas emissions of meat-eaters, fish-eaters, vegetarians and vegans in the UK. *Climatic Change* 125(2): 179-192.

Seabra JEA, Macedo IC, Chum HL, Faroni CE and Sarto CA (2011). Life cycle assessment of Brazilian sugarcane products: GHG emissions and energy use. *Biofuels, Bioproducts and Biorefining* 5(5): 519-532.

Sheane R, Lewis K, Hall P, Holmes-Ling P, Kerr A, Stewart K and Webb D (2011). *Identifying Opportunities to Reduce the Carbon Footprint Associated with the Scottish Dairy Chain – Main Report.* Edinburgh: Scottish Government.

Svanes E and Aronsson AKS (2013). Carbon footprint of a Cavendish banana supply chain. *International Journal of Life Cycle Assessment* 18(8): 1450-1464.

Wiedemann S, McGahan E, Murphy C, Yan M-J, Henry B, Thoma G and Ledgard S (2015). Environmental impacts and resource use of Australian beef and lamb exported to the USA determined using life cycle assessment. *Journal of Cleaner Production* 94: 67-75.

Ziegler F, Winther U, Skontorp Hognes E, Emmanuelsson A, Sund V, Ellingsen H (2012). The carbon footprint of Norwegian seafood products on the global seafood market. *Journal of Industrial Ecology* 17(1): 103-116

For peer review only

Appendix 7A – Calculation of weighted GHG and water footprint of the food group aggregate "fruit and vegetables" – based on proportional supply by crop and country of origin (FAOStat 2013 data)

Crops	% of total supply	GHG per kg	Blue WF per kg
Tomatoes	13.4	0.96	36
Bananas	7.8	1.07	49
Grapes**	6.3	1.34	298
Apples	6.1	0.75	77
Onions, dry	6.0	0.76	36
Carrots and turnips	5.8	1.28	0
Oranges	5.0	0.4	93
Cauliflowers and broccoli	2.5	1.03	53
Cabbages and other brassicas	2.5	0.67	7
Lettuce and chicory	2.3	1.59	41
Tangerines, mandarins, clementines, satsumas	2.0	0.4	93
Mushrooms and truffles	1.6	0.76	2
Chillies and peppers, green	1.6	0.76	2
Cucumbers and gherkins	1.5	0.2	12
Olives**	1.4	1.34	298
Pineapples**	1.4	1.34	298
Maize, green	1.3	0.76	2
Peas, green	1.3	2.55	23
Pears**	1.3	0.75	77
Melons, other (inc.cantaloupes)**	1.1	1.34	298
Strawberries	1.0	1.7	28
Lemons and limes	0.84	1.01	157
Plums and sloes	0.82	1.7	28
Pumpkins, squash and gourds	0.75	0.5	55
Peaches and nectarines**	0.64	1.34	298
Grapefruit (inc. pomelos)	0.60	0.4	93
Watermelons**	0.53	1.34	298
Apricots**	0.46	1.34	298
Sweet potatoes	0.46	0.5	55
Leeks, other alliaceous vegetables	0.42	0.71	15
Mangoes, mangosteens, guavas**	0.41	1.34	298
Beans, green	0.36	0.5	6
Avocados**	0.25	1.34	298
Onions, shallots, green*	0.24	0.76	36

* all other crops contribute less than 0.2% to total UK supply and were disregarded for calculations of weighted GHGe and WF of the fruit and vegetables aggregate.

** Classified as "other fruit" or "other vegetables" - without specific WFs

For peer review only

Appendix 7B - Proportions of main UK imported foods from different countries

For each food group, countries of origin were selected based on a database adapted from FAO Food Balance Sheet data (https://iopscience.iop.org/article/10.1088/1748-9326/9/3/034015/meta). All countries providing at least 10% of the total availability for that food group were included in environmental footprinting, and for the remainder of supply global average figures were applied. Where country-specific footprint data were not available, footprints from the most similar country or a global average were applied.

Food	Country	Proportion of UK consumption
ALMONDS	USA	0.72
	Australia	0.1
	Spain	0.14
	Global	0.04
APPLE JUICE	France	0.14
	South Africa	0.1
	UK	0.36
	Global	0.4
APPLES	France	0.14
	South Africa	0.1
	UK	0.36
	Global	0.4
ASPARAGUS	Mexico	0.11
	Peru	0.46
	Spain	0.12
	UK	0.26
	Global	0.05
AVOCADO	Chile	0.22
	Israel	0.16
	Peru	0.21
	South Africa	0.27
	Global	0.14
BANANAS	Colombia	0.23

For peer review only

	Costa Rica	0.18
	Dominican Republic	0.18
	Ecuador	0.14
	Global	0.27
BARLEY	UK	0.81
	Global	0.19
BEEF	UK	0.76
	Ireland	0.17
	Global	0.07
BLUEBERRIES	Netherlands	0.34
	Poland	0.3
	Global	0.45
CAULIFLOWER/BROCCOLI	UK	0.47
	Spain	0.43
	Global	0.1
CHICKPEAS	Argentina	0.11
	Australia	0.21
	Canada	0.14
	Mexico	0.12
	Global	0.42
COFFEE	Brazil	0.26
	Colombia	0.1
	Indonesia	0.13
	Vietnam	0.25
	Global	0.26
COCOA BUTTER	Ghana	0.27
	Cote d'Ivoire	0.44
	Nigeria	0.1
	Global	0.19

For peer review only

COCOA PASTE	Ghana	0.27
	Cote d'Ivoire	0.44
	Nigeria	0.1
	Global	0.19
COCONUTS	Indonesia	0.24
	Malaysia	0.11
	Philippines	0.48
	Global	0.17
CUCUMBERS	Netherlands	0.34
	Spain	0.33
	UK	0.3
	Global	0.03
GARLIC	China	0.41
	Spain	0.52
	Global	0.07
GRAPES	Chile	0.1
	France	0.1
	Italy	0.15
	Spain	0.1
	Turkey	0.18
	Global	0.37
GREEN BEANS	Egypt	0.15
	Kenya	0.32
	UK	0.32
	Global	0.21
GROUNDNUTS	USA	0.26
	Nicaragua	0.13
	China	0.14
	Argentina	0.33
	Global	0.14

For peer review only

HAZELNUTS	Georgia	0.18
	Italy	0.1
	Turkey	0.69
	Global	0.03
LEMON JUICE	Argentina	0.16
	Brazil	0.13
	Spain	0.47
	Global	0.24
LEMONS	Argentina	0.16
	Brazil	0.13
	Spain	0.47
	Global	0.24
LENTILS	Canada	0.63
	Turkey	0.26
	Global	0.11
LETTUCE	Spain	0.49
	UK	0.4
	Global	0.11
MAIZE	France	0.27
	Ukraine	0.18
	Global	0.55
MILLET	Russia	0.32
	France	0.24
	Ukraine	0.13
	India	0.1
	Global	0.21
NUTS, OTHER	China	0.13
	Turkey	0.3
	USA	0.12
	Global	0.45

For peer review only

OLIVE OIL	Italy	0.18
	Spain	0.59
	Global	0.23
ONIONS	Netherlands	0.23
	Spain	0.16
	UK	0.44
	Global	0.17
ORANGE JUICE	Brazil	0.3
	South Africa	0.13
	Spain	0.28
	USA	0.1
	Global	0.19
ORANGES	Brazil	0.3
	South Africa	0.13
	Spain	0.28
	USA	0.1
	Global	0.19
PALM OIL	Indonesia	0.43
	Malaysia	0.28
	Papua New Guinea	0.2
	Global	0.09
PEACHES	Italy	0.14
	Spain	0.73
	Global	0.13
PEARS	Netherlands	0.32
	South Africa	0.17
	UK	0.13
	Global	0.38
PEPPERS AND CHILLIES	Netherlands	0.38
	Spain	0.34

For peer review only

	UK	0.11
	Global	0.17
PINEAPPLES	Costa Rica	0.7
	Thailand	0.14
	Global	0.16
POTATOES	UK	0.72
	Netherlands	0.11
	Global	0.17
PRUNES (PLUMS)	Chile	0.24
	Spain	0.16
	UK	0.11
	USA	0.14
	Global	0.35
RAISINS	Turkey	0.3
	USA	0.3
	South Africa	0.1
	Chile	0.1
	Global	0.2
RICE (WHITE)	India	0.26
	Spain	0.18
	Italy	0.13
	Pakistan	0.11
	Global	0.32
RICE (BROWN)	India	0.26
	Spain	0.18
	Italy	0.13
	Pakistan	0.11
	Global	0.32
SOYA PROTEIN	Brazil	0.44
	Argentina	0.31

For peer review only

	USA	0.16
	Global	0.09
SOYBEANS	Argentina	0.31
	Brazil	0.44
	USA	0.16
	Global	0.09
SOYBEANS FOR MILK	Brazil	0.44
	Argentina	0.31
	USA	0.16
	Global	0.09
SPINACH	Italy	0.11
	Spain	0.7
	Global	0.19
SUGAR	UK	0.38
	Global	0.62
SUNFLOWER OIL	Argentina	0.12
	France	0.14
	Ukraine	0.31
	Global	0.43
SUNFLOWER SEEDS	Argentina	0.12
	France	0.14
	Ukraine	0.31
	Global	0.43
TEA	India	0.16
	Indonesia	0.15
	Kenya	0.39
	Global	0.3
TOMATO PASTE	Italy	0.39
	Spain	0.2
	Portugal	0.13

For peer review only

	Global	0.28
TOMATOES	Italy	0.39
	Spain	0.2
	Portugal	0.13
	Global	0.28
WHEAT FLOUR	UK	0.67
	Global	0.33

Appendix 8 – Differences in EWG dietary guidelines adherence

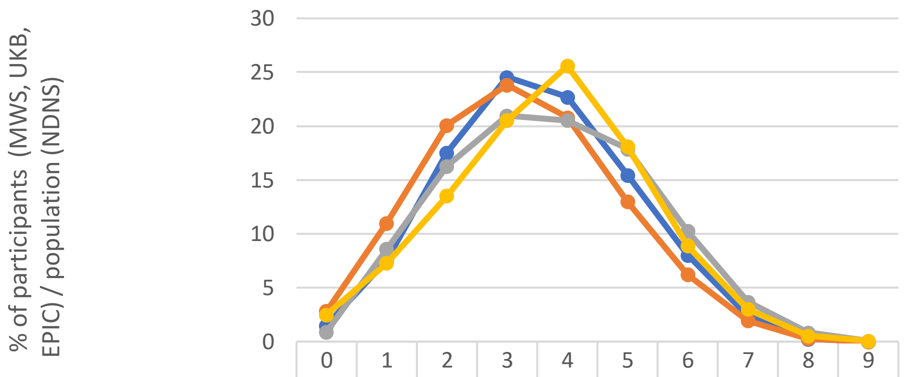

	0	1	2	3	4	5	6	7	8	9
Million Women Study	1.48	7.56	17.54	24.55	22.71	15.44	8.04	2.40	0.26	0.01
UK Biobank	2.84	11.00	20.09	23.83	20.80	13.00	6.23	1.93	0.27	NA
EPIC Oxford	0.89	8.58	16.28	20.97	20.54	17.88	10.25	3.69	0.85	0.08
NDNS	2.50	7.28	13.52	20.52	25.58	18.08	8.90	3.01	0.54	0.08
